# PTMViz: a tool for analyzing and visualizing histone post translational modification data

**DOI:** 10.1186/s12859-021-04166-9

**Published:** 2021-05-26

**Authors:** Kevin Chappell, Stefan Graw, Charity L. Washam, Aaron J. Storey, Chris Bolden, Eric C. Peterson, Stephanie D. Byrum

**Affiliations:** 1 Department of Biochemistry and Molecular Biology, University of Arkansas for Medical Sciences, 4301 W Markham St. slot 516, Little Rock, AR 72205 USA; 2 Arkansas Children’s Research Institute, Little Rock, AR 72202 USA; 3 Department of Pharmacology and Toxicology, University of Arkansas for Medical Sciences, Little Rock, AR 72205 USA

**Keywords:** Proteomics, Histone post-translational modifications, Differential abundance, Shiny

## Abstract

**Background:**

Histone post-translational modifications (PTMs) play an important role in our system by regulating the structure of chromatin and therefore contribute to the regulation of gene and protein expression. Irregularities in histone PTMs can lead to a variety of different diseases including various forms of cancer. Histone modifications are analyzed using high resolution mass spectrometry, which generate large amounts of data that requires sophisticated bioinformatics tools for analysis and visualization. PTMViz is designed for downstream differential abundance analysis and visualization of both protein and/or histone modifications.

**Results:**

PTMViz provides users with data tables and visualization plots of significantly differentiated proteins and histone PTMs between two sample groups. All the data is packaged into interactive data tables and graphs using the Shiny platform to help the user explore the results in a fast and efficient manner to assess if changes in the system are due to protein abundance changes or epigenetic changes. In the example data provided, we identified several proteins differentially regulated in the dopaminergic pathway between mice treated with methamphetamine compared to a saline control. We also identified histone post-translational modifications including histone H3K9me, H3K27me3, H4K16ac, and that were regulated due to drug exposure.

**Conclusions:**

Histone modifications play an integral role in the regulation of gene expression. PTMViz provides an interactive platform for analyzing proteins and histone post-translational modifications from mass spectrometry data in order to quickly identify differentially expressed proteins and PTMs.

**Supplementary Information:**

The online version contains supplementary material available at 10.1186/s12859-021-04166-9.

## Background

The field of epigenetics has developed significantly in recent years, leading to a more nuanced understanding of how chromatin interactions and gene accessibility affect protein networks [1, 2]. With the advancement of mass spectrometers and laboratory techniques, we can obtain deeper sequencing coverage of proteins and histone post-translational modifications, leading to thousands of peptides for analysis In order to make biological knowledge from all of the data, we must develop methods of quickly processing and analyzing epigenomic data in an efficient and accurate way.

Histone post translational modifications (PTMs) are a prominent epigenetic factor that affects nearly all genetic pathways within the nucleus. The histones within cells are responsible for binding DNA into tight coils known as chromatin. The core histone proteins (H2A, H2B, H3, and H4) are responsible for forming an octamer complex, known as a nucleosome, with the ability to bind ~ 147 bp of DNA around it at a time [3]. The extent to which a nucleosome can bind the DNA to itself is dependent on the charge of the post translational modifications, which occur on the N-terminal tail of each histone within the complex. Several different combinations of modifications can be integrated into the histone and change its binding properties. Some examples of modifications include phosphorylation, ubiquitination, methylation, and acetylation. When modifications such as methylation are added to the histone N-terminal tails, it changes the charge of the histone to become more positive, leading to tighter DNA binding to the histone, which blocks access of transcription factors and down-regulates gene expression. Acetylation often has the opposite effect of adding a negative charge and thereby loosening the DNA around the nucleosome leading to increased gene expression [4, 5]. A popular method of analyzing histone PTMs is through a variety of high throughput mass spectrometry techniques, such as bottom-up, middle-down, and top-down approaches. A common bottom-up technique involves purifying histones using acid extraction. Histones contain many lysine and arginine residues in the protein sequence, which are the cut sites for a commonly used trypsin digestion. Instead of a typical trypsin digestion, the histones are first treated with deuterated (d6-) acetic anhydride, which converts all unmodified lysines into d6-acetyl-lysines. This modified lysine residue prevents trypsin from cleaving at lysines and generates longer tryptic peptides that are within the detection limits of the mass spectrometer [6]. A middle-down approach involves using the digestion enzyme GluC as opposed to trypsin [7, 8]. GluC cleaves at the C-terminal of the glutamic acid residue, which for histone H3 isotypes the first glutamic acid residue is in position 50. This generates a polypeptide of 40–50 aa residues (5–6 kDa) that contain the majority of histone PTMs and is beneficial for analyzing combinatorial histone modifications. The top-down mass spectrometry approach analyzes intact proteins. These techniques result in complex data that often requires specialized informatics approach to analyze [8, 9].

### Implementation

#### PTMViz overview and data requirements

The PTMViz Visualization tool was constructed within the R (version 3.5.1) programming environment [10]. To create the specific graphical user interface, the packages *Shiny* (1.4.0.2), *Shiny Dashboard* (0.7.1) and *shinyWidgets* (0.5.1) were utilized [11]. Interactive volcano plots were generated using *plotly* (4.9.2) [12]. The *ggplot* (3.3.0) library was used for parallel construction of graphics, and *theRColorBrewer* (1.1) library was used for color themes [13, 14]. The *limma* (3.38.3) package was used for the differential abundance analysis. To address the need for differential abundance analysis and visualization of histone PTMs between sample conditions, we developed PTMViz. Data frame manipulation was performed using the *dplyr* (0.8.5) library, *reshape* (0.8.8), *crosstalk* (1.1.0.1), and *tidyverse* (1.3.0) [15–18].

**Fig. 1:**
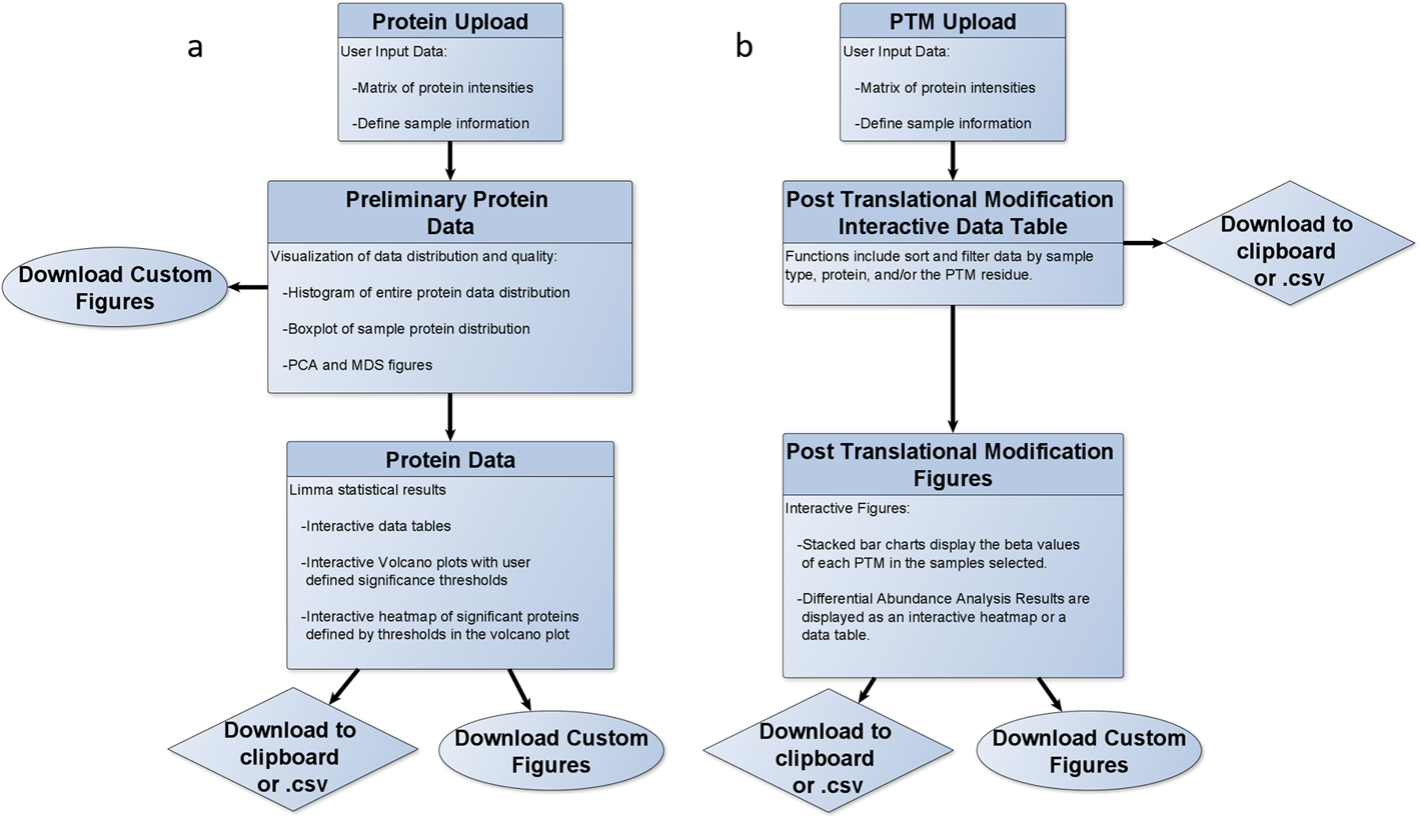
Flowchart that outlines the workflow of PTMViz. The tool has two separate sections that work independently from one another. **a** Protein data analysis includes the user loading in protein intensity data matrix and defining the sample metadata used for the analysis. The data distribution is visualized by various plots including histogram, boxplot, PCA, and MDS presented in the Protein Preliminary Shiny dashboard tab. Limma is used for the differential analysis and results are displayed as interactive data tables and plots in the Protein Data Shiny dashboard tab. **b** PTM data analysis begins with user loaded peptide intensity data and the defining of sample metadata. The data is then organized into interactive data tables and plots, which are displayed in the Post Translational Modification tab of the Shiny dashboard. The significance thresholds for the plots can be modified and the tables can be filtered to search for data of interest interactively

PTMViz not only analyzes histone PTMs but also allows for the analysis of total protein levels in order to investigate changes at both the protein and histone PTM level (regulation of gene expression) between sample conditions. This feature is enhanced through the incorporation of the WERAM database of reader, writer and eraser proteins for different organisms. When a dataset is loaded into PTMViz it will be compared against the database and any common proteins will be pulled into a tab for further investigation [19]. PTMViz is built to incorporate histone post-translational modification peptide abundances, as well as protein abundances obtained through mass spectrometry in the form a comma delimited.csv file within an R Shiny Dashboard environment (Fig. 1). Figure 1 shows the workflow for both the protein analysis (Fig. 1a) and the histone PTM analysis (Fig. 1b). The protein and histone PTM workflows are run independently in PTMViz. Therefore it is not required to have both types of data to utilize the tool. PTMViz allows the user to define the sample and group names, performs differential abundance analysis using *limma*, and displays the results as interactive volcano, stack bar charts, heatmaps, and data tables. This graphical user interface provides a unique and powerful way for a user to have the ability to scrutinize data through searches, sample group comparisons, and interactive visualizations. PTMViz data and R scripts are available in Additional file 1.

#### Comparison to other software

Two of the more prominent software packages available to analyze complex histone PTM relative abundances are Epiprofile2.0 and Skyline [1, 20]. These tools were developed to perform peak area integration. EpiProfile2 is optimized for histone peptides due to the fact it uses the retention time knowledge of chromatographic elution to perform a more reliable peak area extraction of known histone peptides. Alternatively, Skyline can also be used to extract peak area information [7]. The output data generated by Epiprofile and Skyline can be used as the input data for PTMViz, which will then perform differential abundance analysis of the histone PTMs as well as any protein data that is available. This allows PTMViz to function as a downstream tool that is capable of integrating the results from other analysis software such as Epiprofile2.0 and Skyline. Often the biological question is not only how abundant is the histone PTM within a sample but is this modification significantly changed due to an experimental condition?

A current workflow and guidelines for analyzing histone post-translational modifications is described in Thomas et al. The workflow includes normalizing modifications using the total intensity method, where each modification is divided by the sum of all modifications in the sample, not just those in the peptide family. This allows for the detection of protein abundance differences between samples while being robust to peptides with any number of modifications. Once the data is normalized, the guidelines state the current standard for statistical analysis of PTM data is to calculate the fold change for each modification and perform a classical Student’s t-test. Thomas et al. added a workflow to include the analysis of variance (ANOVA) method, which selects modifications that are significant in at least one condition, and then calculates Tukey’s HSD to generate *p* values when several conditions are being evaluated.

In contrast to previous analyses, PTMViz performs a moderated t-test statistical analysis by incorporating the variance in the dataset using limma. We provide an additional script outside of the PTMViz tool that allows for the transformation of relative abundance values to logit transformed M-values for statistical analysis. PTMViz provides flexibility in the upstream normalization process by allowing various normalized values to be imported into the tool. As long as the data is in a form acceptable for limma, the modifications can be normalized using the total intensity method (as described in Thomas et al.) or the percent of total peptide family method [15, 21, 22].

## Results

To illustrate the use of PTMViz, we demonstrate the tool features on a drug abuse study in which mice were given acute injections of either a saline control or methamphetamine. The details of the animal treatments and protein and histone sample preparation are described in Graw et al. 2020 and in Additional file 2 [23]. The reward circuitry nucleus accumbens and dorsal striatum brain regions were harvested to test for protein and histone PTM changes due to methamphetamine drug exposure. Proteins from the whole cell lysate as well as acid extracted histones were sequenced by high resolution mass spectrometry and analyzed by PTMViz. We identified 15 out of 3,163 proteins and 3 out of 580 histone PTMs with significantly differentiated changes due to drug exposure in the nucleus accumbens. These proteins and PTMs have also been identified by other studies and validate the functionality of PTMViz [24–27].

Within the tool the user has the option to analyze either the protein and/or PTM data using the tabs on the left hand side of the graphical user interface. The protein input and results are visualized using the “protein” tab and the histone PTMs input and results are located on the “PTMs” tab. Both tabs work independently and do not require input from one another to operate. Both analysis tabs provide interactive plots and tables to explore the analytical results.

### Protein data analysis

The protein analysis work flow is graphically displayed in Fig. 1a. There are three sub tabs included for the “protein” analysis tab on the Shiny Dashboard including 1. Protein upload, 2. Preliminary, and 3. Protein Data. To begin the protein analysis, the user is required to upload the “protein data” and input the “metadata” information in the protein upload tab. The protein data consists of a comma delimited file containing a matrix of MS1 protein abundances for each sample. The user is then required to define the sample metadata, which will define the sample names and groups to be compared in the differential abundance analysis (Fig. 1a). The required metadata includes a label for each sample group (ex: tissue region, cell type, etc.), the biological or technical replicate number of the sample, the experimental group to be analyzed for differential abundance analysis (ex: treatment vs control), and an optional custom identification name (unique and will be displayed on the plots). An example metadata table for the example data set is provided in Table 1. By providing these metadata labels, the user will have control over how the figures display information and what sample group comparisons are being performed. Once the metadata has been completed, the analysis will automatically run and display the results in the analysis tab of the GUI.

**Table 1:** Sample metadata table that accompanies the input of the protein data

File name	Sample group	Replicate	Experimental group	Custom ID
Reporter.intensity.corrected.0	Nucleus Accumbens	1	Treatment	NA_1_T
Reporter.intensity.corrected.1	Nucleus Accumbens	2	Treatment	NA_2_T
Reporter.intensity.corrected.2	Nucleus Accumbens	3	Treatment	NA_3_T
Reporter.intensity.corrected.3	Nucleus Accumbens	1	Control	NA_1
Reporter.intensity.corrected.4	Nucleus Accumbens	2	Treatment	NA_2
Reporter.intensity.corrected.5	Nucleus Accumbens	3	Treatment	NA_3

This table allows the user to define the sample groups, each samples replicate number, the experimental group for the differential analysis, and a unique custom id that is displayed in the figures

Next, the protein data characteristics can be visualized using the “Preliminary” tab, which includes a histogram of the entire protein abundance distribution, boxplots displaying the log2 abundances for each sample, and principal component analysis (PCA) plot of the first two PC components. Next, PTMViz generates a “Protein Data” tab which displays the results from a differential abundance analysis by the limma R package. The obtained fold change, *p* value, and false discovery rate (FDR) adjusted *p* value information is then utilized to create interactive figures such as a volcano plot and heatmap. The interactive plots allow for easy exploration of significant proteins and protein patterns.

**Fig. 2:**
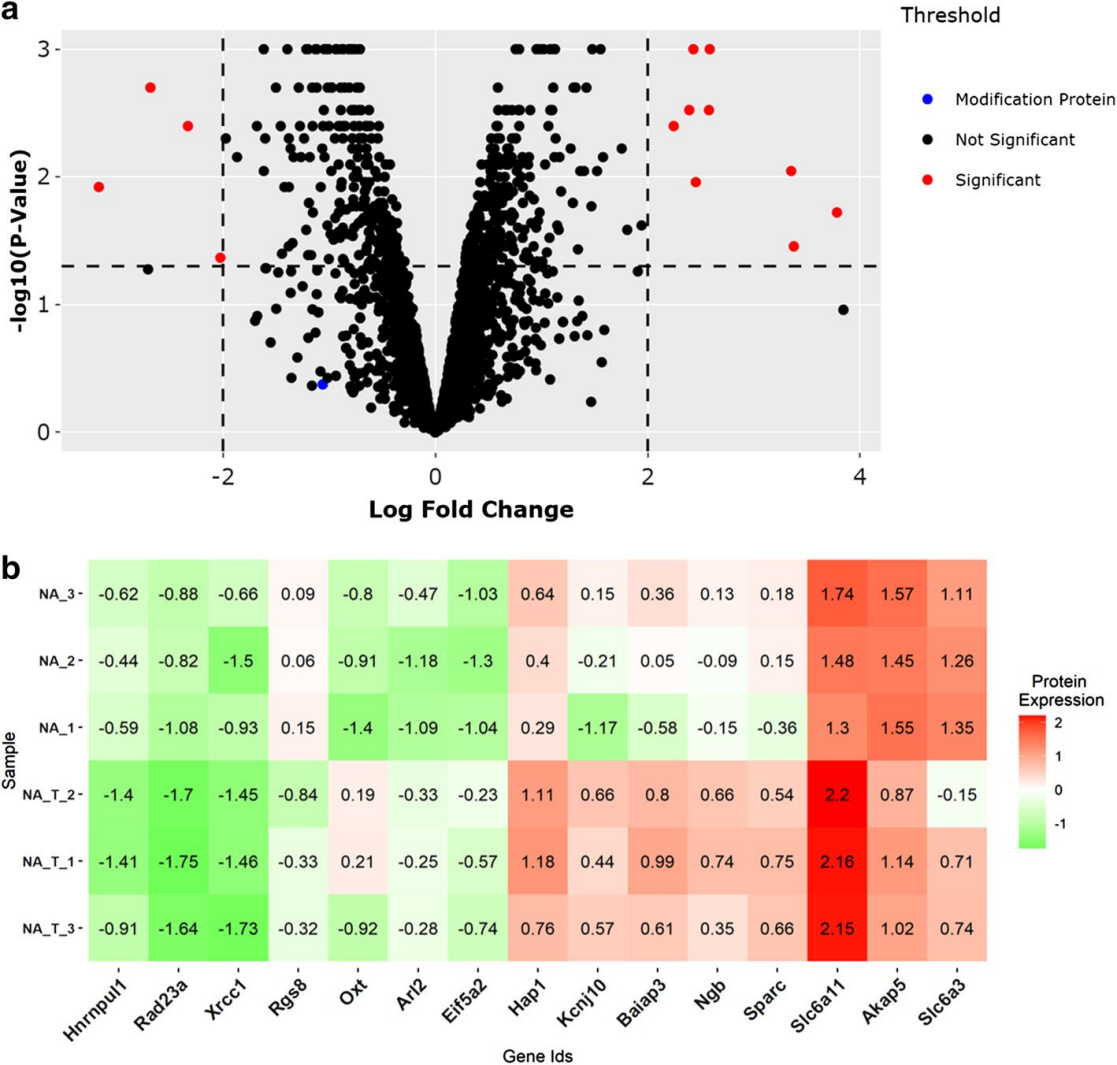
Displays the primary figures obtained from the protein section of PTMViz. **a** The top figure displays a volcano plot with the log2 fold change on the x-axis and either the *p* value or the FDR adjusted *p* value on the y-axis depending on the options selected. The points on the plot are interactive such that hovering the cursor over each individual point shows the protein Uniprot id, gene id, description, log2 fold change, and *p* value. Proteins that are identified as significant based on user defined thresholds (dashed lines) are highlighted in red. Modifying proteins that match to the WERAM database are highlighted in blue. Multiple points on the graph can be selected and viewed in the primary table to allow for the export of points of interest. **b** A heatmap of the z-score scaled log2 normalized protein intensities for the proteins identified as significant in the Volcano plot

The interactive volcano plot is generated by the R package *plotly*. Figure 2a represents an interactive volcano plot displaying the log2 fold change values comparing methamphetamine treatment versus saline controls in the nucleus accumbens on the x-axis and the FDR adjusted *p* values on the y-axis (Fig. 2a). Within the volcano plot the user can hover the cursor over the individual data points to identify specific proteins, log_2_ fold change, and *p* values. This helps to identify which proteins are up- or down-regulated and pass statistical significance. Additionally, the volcano plot highlights proteins identified in the Writers, Erasers and Readers of Acetylation and Methylation (WERAM) database [19]. The threshold labels on the side of the volcano plot can be selected to show only the WERAM proteins on the volcano plot in order to visualize clearly if these modifying proteins are significant in the protein analysis (Additional file 2: Fig. S1). One such modifying protein, Prmt5, was identified as slightly down-regulated in mice treated with methamphetamine. It has been shown the global level of Histone H3K27me3 increases with decreasing Prmt5 [28]. We identified 15 significant proteins between drug treated and saline controls, including Oxytocin (Oxt) and sodium- and chloride-dependent GABA transporter 3 (Slc6a11). Thresholds for significance can be modified using the “settings” tab on the volcano plot, which are visualized through dashed lines on the graph. These thresholds represent the *p* value and log2 fold change and are used to identify statistically significant proteins.

Another popular method of showcasing the protein expression differences between sample groups is through a clustered heatmap. PTMViz utilizes an interactive heatmap produced by the R package *plotly*. The interactive plot allows the user to hover over individual tiles of the heatmap to view the gene id and the z-score intensity value of the particular sample that is being viewed. Figure 2b displays the z-score scaled log2 normalized protein abundances of those proteins that are differentially expressed based on the same thresholds set for the volcano plot.

Additionally, data tables are provided, which include the limma results for all proteins, the proteins that are significant with an FDR *p* value < 0.05 and fold change > 2, the normalized protein abundances for all proteins and samples, and the normalized abundances for each of the significant proteins. A common feature that is ubiquitous throughout the tool are interactive data tables. The purpose of the data tables in our tool is to provide the user with organized data that is easy to search. Some of the data tables have filters and/or are linked to graphs that allow the user to exclude undesired data points and identify results of interest. All the tables come with the option to download the full table as either a csv, copy the data to clipboard, or create a pdf of the table. This allows for additional figures to be created in other software.

### Post translational modification analysis

Under the PTM tab on the left hand side of the Shiny Dashboard, the tool is designed to clearly demonstrate the types of modifications within each sample and identify significantly differentiating modifications between two groups. The workflow for the PTM analysis is displayed in Fig. 1b. Similar to the protein analysis, the user is required to upload a comma delimited file in the “PTM upload” tab which contains the post-translational modifications as rows and sample name, intensities, relative abundance values calculated as beta-values and m-values as columns. Beta-values are commonly used in DNA methylation data analyses. In the context of histone PTMs, a beta-value can be interpreted as the intensity percentage of a specific modification out of total measured intensities of a given PTM site. Analogously to DNA methylation, an offset of 100 is added to the denominator to regularize beta values for low intensities. As the beta-value is bounded by 0 and 1, it violates Gaussian distribution assumption of many statistical methods [29].

The m-value is a logit-transformation of the beta value. The m-value can be appropriately analyzed by such statistical methods [29], such as limma. Please note the variance correction in limma becomes imprecise for low number of features. An R script, “Histone PTM relative abundance.R”, is included with the PTMViz application in Github to calculate PTM beta and m-values from a matrix of peptide intensities as additional support. Similar to the protein data tab, the user will need to provide the sample metadata to define the samples and the group comparisons. The analysis results will then be displayed in the Post Translational Modifications tab of the Shiny Dashboard.

**Fig. 3:**
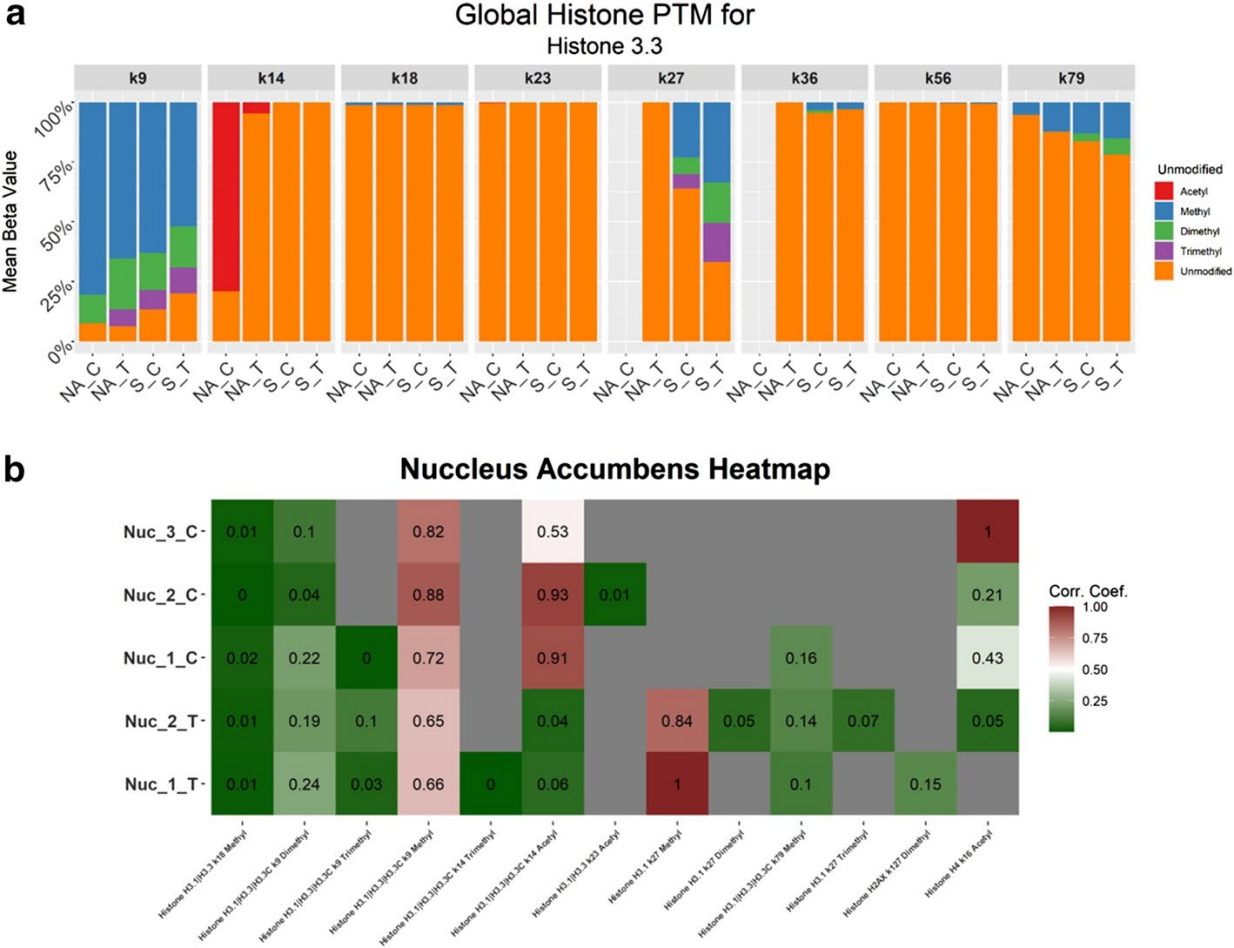
PTM analysis interactive figures. **a** Stacked bar chart demonstrating the global PTMs identified for Histone H3.3. The mean of all sample replicates beta values for each group and PTM are displayed. **b** Heatmap of significant PTMs identified from the limma differential abundance analysis. The M-value of each histone PTM is displayed for each sample

The first feature of the PTM tab is an interactive data table, displaying all of the modifications included in the analysis, where the user can filter the data to observe specific samples, histone proteins, histone amino acid positions, or modification type. The histone PTM analysis is summarized within a stacked bar plot, showcasing the different modification types by displaying the beta values, percentage of a given modification out of the total signal in that peptide, at each amino acid position within a histone. In the sample data provided, we identified significantly differentiating histone PTMs including histone H3K9me, H3K27me3, and H4K16ac, that were regulated due to drug exposure (Fig. 3, Additional file 2: Fig. S2) and are visualized in the stacked bar chart and heatmap. These modifications have been identified in previous experiments [25–27]. Visualization options include but are not limited to: toggling between the sample groups, viewing either the average or individual sample replicates, and selecting specific amino acid locations on a histone protein (Fig. 3a).

Next, the tool features a differential analysis table, including the limma results, where the user can visualize the significant modifications. Within this section the user can select the two groups of data they wish to compare and visualize as a differential analysis and in an interactive heat map (Fig. 3b) similar to the heatmap created in the protein section of the tool. All these features give the user the control to view their data in many ways to achieve a maximum understanding of the results.

## Conclusion

As mass spectrometry has advanced and allows for the detection of thousands of proteins and histone post-translational modifications, there is a growing need for differential analysis of PTMs in biological conditions. Tools such as Epiprofile 2.0 and Skyline are valuable methods to extract peak areas from mass spectrometry data. The data can then be analyzed further using PTMViz to perform differential analysis between sample conditions and interactive plots can be used to explore the results.

We applied the PTMViz to explore the effects histone post-translational modifications of drug exposure to the nucleus accumbens in the mouse brain and identify potential modifying proteins that write, erase, or read the significant modifications. We were able to identify known protein and histone PTMs that change due to drug exposure to provide positive controls for the analysis. Through the use of PTMViz, we were able to identify 15 significant proteins and 3 significant PTMs that are a result of acute methamphetamine drug exposure in the nucleus accumbens in mice. Some of the more biologically significant results include proteins that belong to the SLC6 neurotransmitter transporters and oxytocin proteins. In previous studies it has been shown that methamphetamine and other psychostimulants can cause lasting changes to these pathways. For example, *Cadet, Jean Lud *et al*.* showed a similar fold change increase to *Oxt* mRNA in the nucleus accumbens of male rats when dosed with methamphetamine. Additionally, the *SLC6* proteins are shown to be common targets of psychostimulants and are linked to drug abuse [24]. Within the PTM analysis, we also found H4K16ac and H3K9me regulations in the methamphetamine exposed group, agreeing with another study showing that (1) H4K16 experiences a global decrease in acetylation and (2) that H3K9 is methylated as a result of methamphetamine exposure within mouse brain [30]. For greater integration of the two datasets we identified a writer protein known as prmt5 which is slightly downregulated in treatment group. Previous studies show this downregulation can lead to an increase in H3K27me3, which we identified as significant in the modification analysis [28]. This demonstrates that other potential reader, writer and eraser proteins can be potentially identified and investigated using the PTMViz tool through the use of both histone and protein datasets.

In conclusion, the PTMViz tool was designed using R Shiny to assist users in downstream analysis of proteomic histone post-translational modifications between sample conditions. Through the use of this tool users can obtain publication quality figures, explore interactive plots, and data tables.

## Supplementary Information

**Additional file 1.** The data and PTMviz scripts.

**Additional file 2.** Description of mass spectrometry methods.

## Data Availability

Project Name: PTMViz. Project home page: https://github.com/ByrumLab/PTMViz Operating System: Linux, MacOS, Windows. Programming Language: R License: Apache. Any restrictions to use by non-academics: none.

## Abbreviations

PTM: Post-translational modification
NA: Nucleus accumbens
S: Striatum
