# Supplementary material for: PTMViz: a tool for analyzing and visualizing histone post translational modification data
Source: BMC Bioinformatics. 2021 May 26;22:275. doi: 10.1186/s12859-021-04166-9 (PMC8157737; doi:10.1186/s12859-021-04166-9)

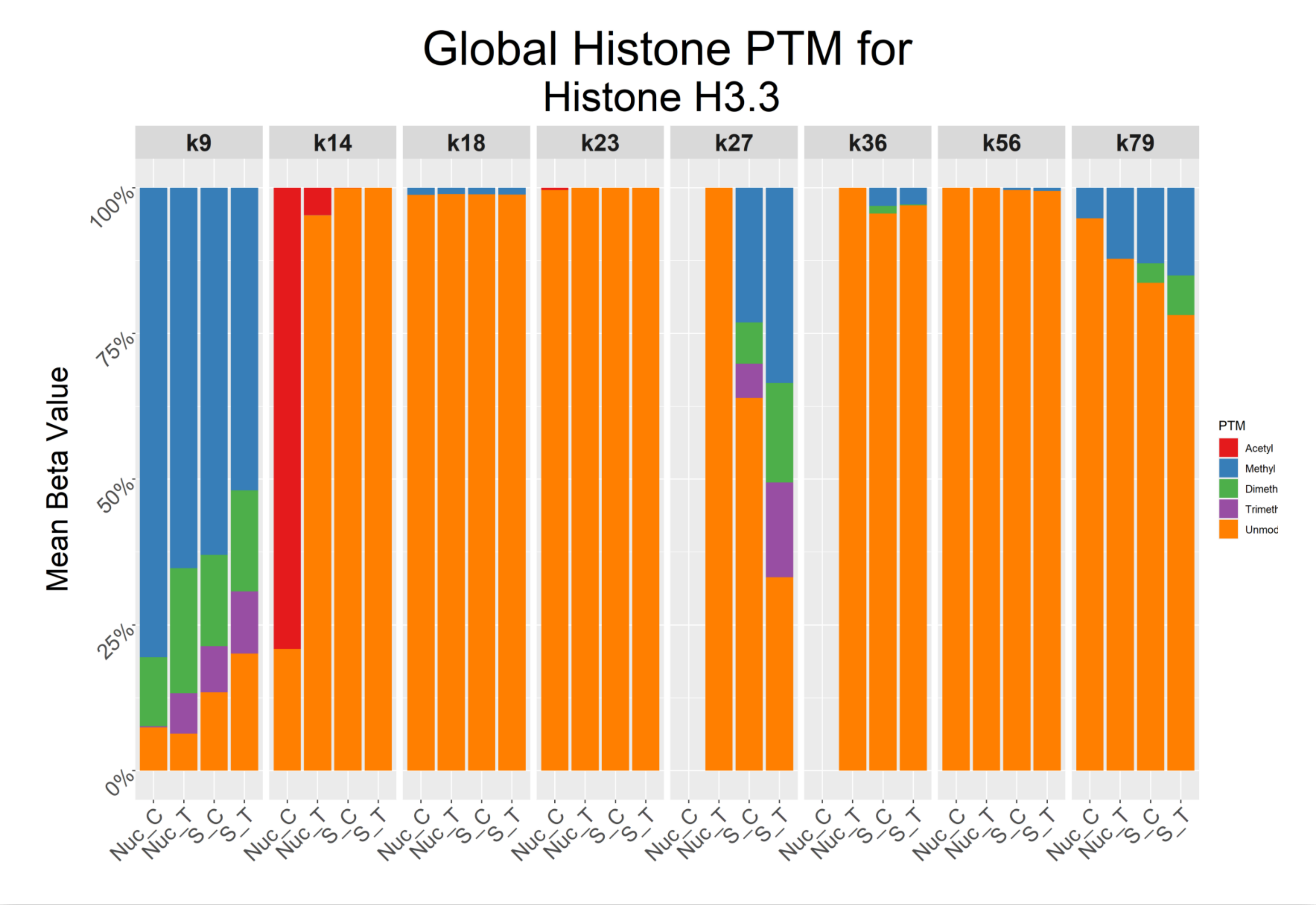
Global Histone PTM for
Histone H3.3
k9
k14
k18
k23
k27
k36
k56
k79
Mean Beta Value
100%
75%
50%
25%
0%
Nuc_C
Nuc_T
S_C
S_T
PTM
Acetyl
Methyl
Dimeth
Trimeth
Unmod

PTMViz

Protein Data Format

- The protein upload section of the tool will need the first column to consist of the fasta header. This is followed by the reporter intensity corrected values.
- All of this data should be available through the protienGroups file of a Mascot Analysis
- Data should be normalized before loading into the PTMViz tool

Fasta headers	Reporter intensity corrected 0	Reporter intensity corrected 1	Reporter intensity corrected 2	Reporter intensity corrected 3	Reporter intensity corrected 4	Reporter intensity corrected 5
tr|A0A0R4IZX5|A0A0R4IZX5_MOUSE Neurocan core protein OS=Mus musculus OX=10090 GN=Ncan PE=1 SV=1;sp|P55066|NCAN_MOUSE Neurocan core protein OS=Mus musculus OX=10090 GN=Ncan PE=1 SV=1	23.12505722	23.57345963	23.00469	24.32194	24.511	23.87233
tr|Q8BGR3|Q8BGR3_MOUSE Calcium/calmodulin-dependent protein kinase IV OS=Mus musculus OX=10090 GN=Camk4 PE=1 SV=1;sp|P08414|KCC4_MOUSE Calcium/calmodulin-dependent protein kinase type IV OS=Mus musculus OX=10090 GN=Camk4 PE=1 SV=2	20.91867065	19.90232468	21.50282	21.934	21.45201	21.94652

Histone PTM Data Format

- For the histone data, you will need to have all of the groups listed in the order of the table below
- One option is to take the S1 file from your run and use the code in the S1 to S3 folder to convert your data into the right format

MS/MS_sample_name	Protein_name	PTM_residues	PTM_corrected	Intensity	Total_intensity	Abundance	betaValue	MValue
Tackett_062118_NA1-T.raw (F050017)	Histone H1.0 OS=Mus musculus GN=H1f0 PE=2 SV=4	k101	k101: Unmodified	81358.32	81358.32	1	0.998772	9.668146
Tackett_062118_NA1-T.raw (F050017)	Histone H2A type 1-F OS=Mus musculus GN=Hist1h2af PE=1 SV=3	k95	k95: Unmodified	93976.24	93976.24	1	0.998937	9.876152

Navigating PTMViz

- Use the Bar on left hand side to navigate the pages of PTMViz

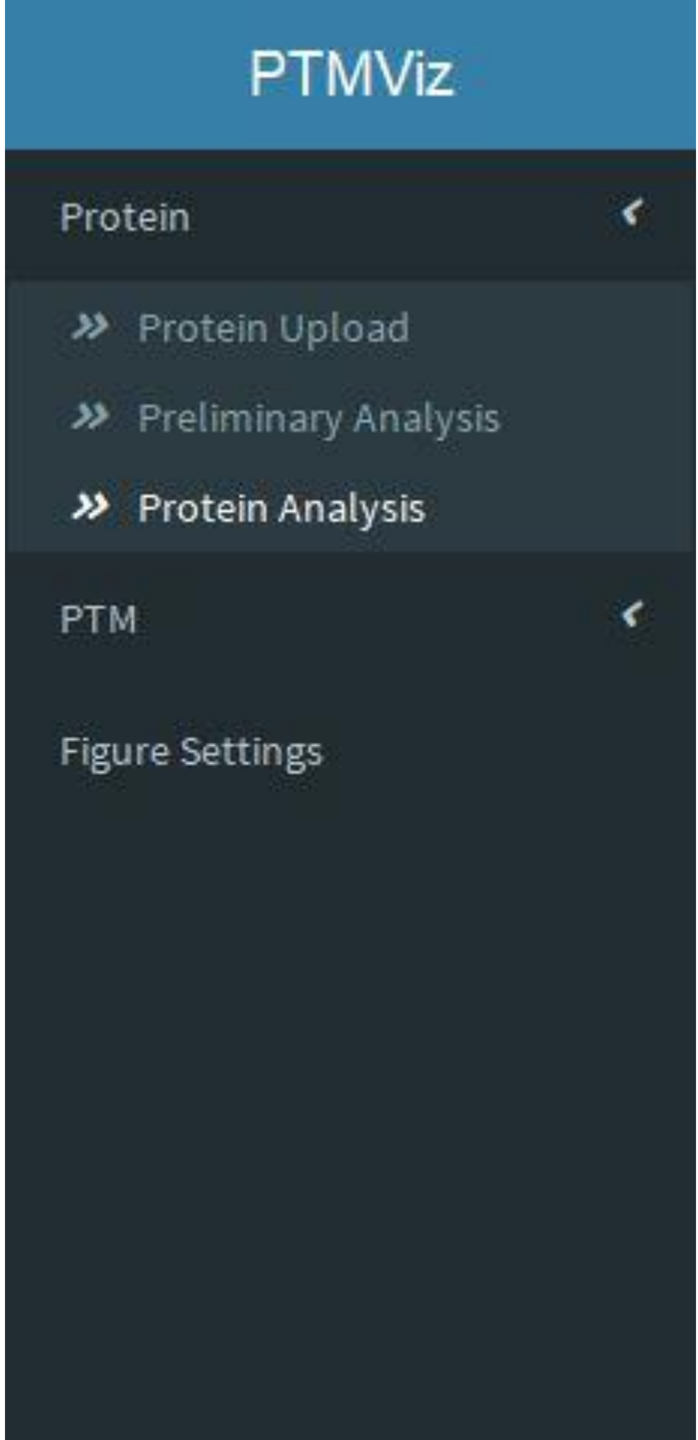

Uploading Data

- Choose Protein Upload or PTM Upload
- Click Browse and navigate to the .csv file that is to uploaded into the tool

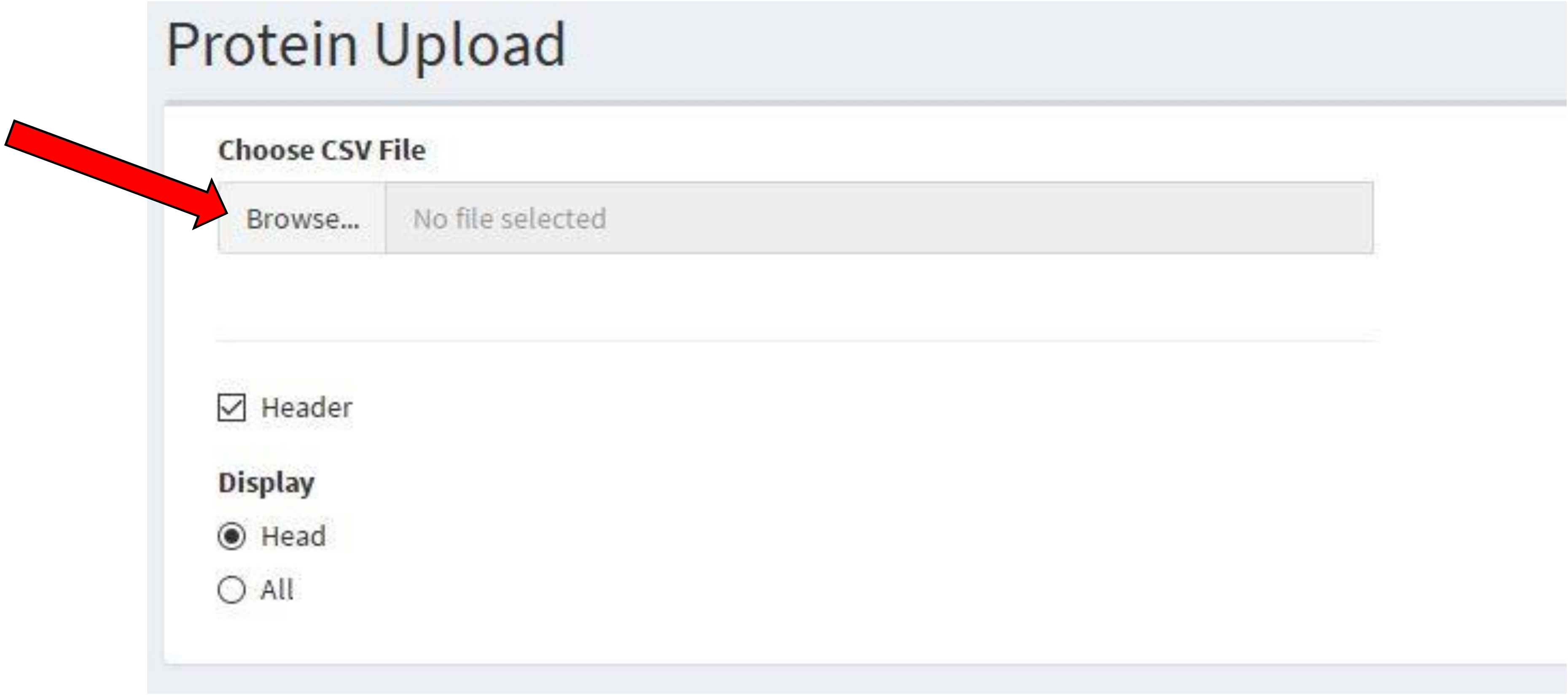

View Uploaded Data

- The window to the right should display the csv file being uploaded into the tool

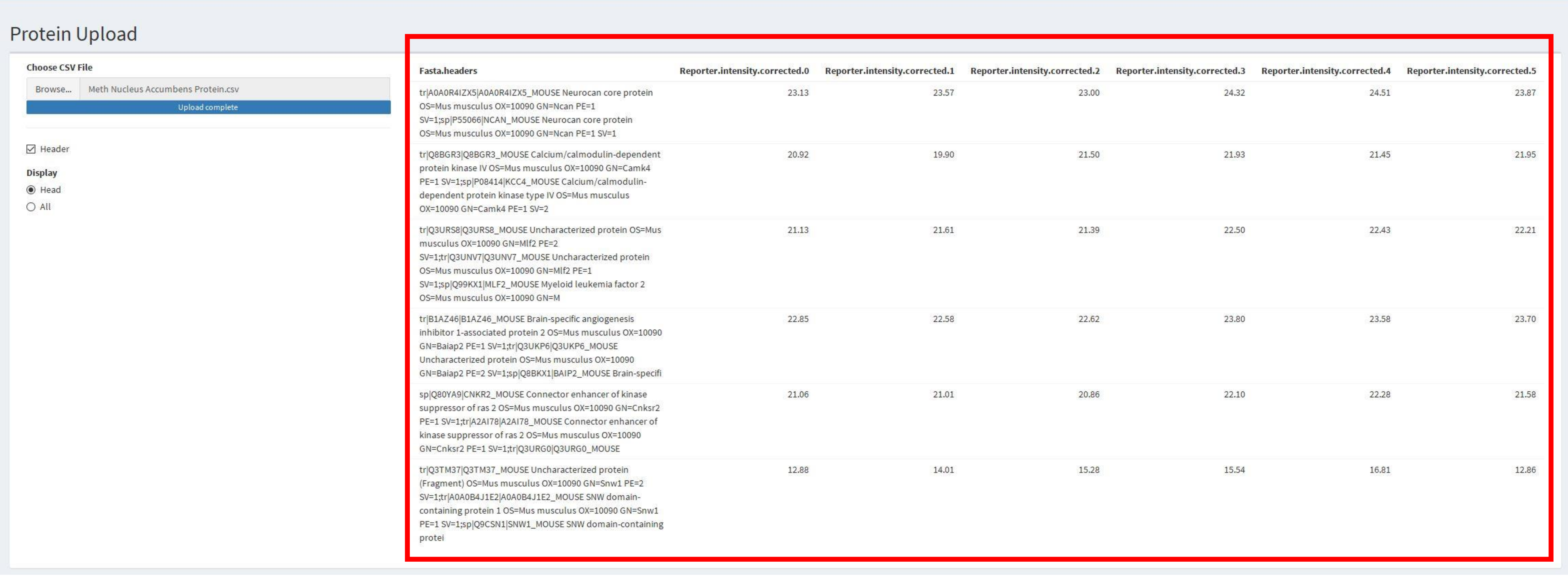

Fasta.headers	Reporter.intensity.corrected.0	Reporter.intensity.corrected.1	Reporter.intensity.corrected.2	Reporter.intensity.corrected.3	Reporter.intensity.corrected.4	Reporter.intensity.corrected.5
tr|A0A0R4IZX5|A0A0R4IZX5_MOUSE Neurocan core protein OS=Mus musculus OX=10090 GN=Ncan PE=1 SV=1;sp|P55066|NCAN_MOUSE Neurocan core protein OS=Mus musculus OX=10090 GN=Ncan PE=1 SV=1	23.13	23.57	23.00	24.32	24.51	23.87
tr|Q8BGR3|Q8BGR3_MOUSE Calcium/calmodulin-dependent protein kinase IV OS=Mus musculus OX=10090 GN=Camk4 PE=1 SV=1;sp|P08414|KCC4_MOUSE Calcium/calmodulin-dependent protein kinase type IV OS=Mus musculus OX=10090 GN=Camk4 PE=1 SV=2	20.92	19.90	21.50	21.93	21.45	21.95
tr|Q3URS8|Q3URS8_MOUSE Uncharacterized protein OS=Mus musculus OX=10090 GN=Mlf2 PE=2 SV=1;tr|Q3UNV7|Q3UNV7_MOUSE Uncharacterized protein OS=Mus musculus OX=10090 GN=Mlf2 PE=1 SV=1;sp|Q99KX1|MLF2_MOUSE Myeloid leukemia factor 2 OS=Mus musculus OX=10090 GN=M	21.13	21.61	21.39	22.50	22.43	22.21
tr|B1AZ46|B1AZ46_MOUSE Brain-specific angiogenesis inhibitor 1-associated protein 2 OS=Mus musculus OX=10090 GN=Baiap2 PE=1 SV=1;tr|Q3UKP6|Q3UKP6_MOUSE Uncharacterized protein OS=Mus musculus OX=10090 GN=Baiap2 PE=2 SV=1;sp|Q8BKX1|BAIP2_MOUSE Brain-specifi	22.85	22.58	22.62	23.80	23.58	23.70
sp|Q80YA9|CNKR2_MOUSE Connector enhancer of kinase suppressor of ras 2 OS=Mus musculus OX=10090 GN=Cnksr2 PE=1 SV=1;tr|A2AI78|A2AI78_MOUSE Connector enhancer of kinase suppressor of ras 2 OS=Mus musculus OX=10090 GN=Cnksr2 PE=1 SV=1;tr|Q3URG0|Q3URG0_MOUSE	21.06	21.01	20.86	22.10	22.28	21.58
tr|Q3TM37|Q3TM37_MOUSE Uncharacterized protein (Fragment) OS=Mus musculus OX=10090 GN=Snw1 PE=2 SV=1;tr|A0A0B4J1E2|A0A0B4J1E2_MOUSE SNW domain-containing protein 1 OS=Mus musculus OX=10090 GN=Snw1 PE=1 SV=1;sp|Q9CSN1|SNW1_MOUSE SNW domain-containing protei	12.88	14.01	15.28	15.54	16.81	12.86

Labeling your data

- Using the table below the upload button label the data based on available metadata

Data Labels

Name of Control

Control

Name of Treatment

Treatment

	File.Name	Sample.Group	Replicate	Experimental.Group	Custom.ID
1	Reporter.intensity.corrected.0			Control	
2	Reporter.intensity.corrected.1			Treatment	
3	Reporter.intensity.corrected.2			Control	
4	Reporter.intensity.corrected.3			Treatment	
5	Reporter.intensity.corrected.4			Control	
6	Reporter.intensity.corrected.5			Treatment	

Update Data

What each column in the Data Label table represents

- Sample Group
  - The Sample Group column, orwill separate your samples into groups. Its best to label these columns based on a common identifier such as the tissue organism, or cell type.
- Replicate
  - This section lets you identify which replicate the sample represents
- Experimental Group
  - The experimental group tells the tool which samples you consider the control and which are treatment. Based on these identifications the tool will perform differential analysis. (In the Protein Upload tab, the user will need to first use the two text boxes up top to denote what they call the treatment and control, if it is labeled differently)
- Custom ID
  - Allows the user to include a custom label for individual samples. This will be included in some graphics. (If left blank, the tool will simply use a combination of the other three identifiers to fill in the blank)

Example from the PTM Upload Section:

- Sample Group
  - The data includes samples from two types of tissues.
- Replicate
  - Data was run in triplicates
- Experimental Group
  - The labels are left as "Treatment" and "Control"
- Custom ID
  - Includes a shortened version of each column to make identification in the tool easier.

	File.Name	Sample.Group	Replicate	Experimental.Group	Custom.ID
1	Tackett_062118_NA1-T.raw (F050017)	Nucleus Accumbens	1	Treatment	NA_T_1
2	Tackett_062118_NA2-T.raw (F050018)	Nucleus Accumbens	2	Treatment	NA_T_2
3	Tackett_062118_NA3-T.raw (F050019)	Nucleus Accumbens	3	Treatment	NA_T_3
4	Tackett_062118_NA4.raw (F050020)	Nucleus Accumbens	1	Control	NA_1
5	Tackett_062118_NA5.raw (F050021)	Nucleus Accumbens	2	Control	NA_2
6	Tackett_062118_NA6.raw (F050022)	Nucleus Accumbens	3	Control	NA_3
7	Tackett_062118_S1-T.raw (F050023)	Striatum	1	Treatment	S_T_1
8	Tackett_062118_S2-T.raw (F050024)	Striatum	2	Treatment	S_T_2
9	Tackett_062118_S3-T.raw (F050025)	Striatum	3	Treatment	S_T_3
10	Tackett_062118_S4.raw (F050026)	Striatum	1	Control	S_1
11	Tackett_062118_S5.raw (F050027)	Striatum	2	Control	S_2
12	Tackett_062118_S6.raw (F050028)	Striatum	3	Control	S_3

Update Data

Alternate Example from the PTM Upload Section:

	File.Name	Sample.Group	Replicate	Experimental.Group	Custom.ID
1	Tackett_062118_NA1-T.raw (F050017)	Nuc	1	T	Nuc_T_1
2	Tackett_062118_NA2-T.raw (F050018)	Nuc	2	T	Nuc_T_2
3	Tackett_062118_NA3-T.raw (F050019)	Nuc	3	T	Nuc_T_3
4	Tackett_062118_NA4.raw (F050020)	Nuc	1	C	Nuc_1
5	Tackett_062118_NA5.raw (F050021)	Nuc	2	C	Nuc_2
6	Tackett_062118_NA6.raw (F050022)	Nuc	3	C	Nuc_3
7	Tackett_062118_S1-T.raw (F050023)	S	1	T	S_T_1
8	Tackett_062118_S2-T.raw (F050024)	S	2	T	S_T_2
9	Tackett_062118_S3-T.raw (F050025)	S	3	T	S_T_3
10	Tackett_062118_S4.raw (F050026)	S	1	C	S_1
11	Tackett_062118_S5.raw (F050027)	S	2	C	S_2
12	Tackett_062118_S6.raw (F050028)	S	3	C	S_3

Update Data

Data Label Verification

- After clicking the upload data button at the bottom of the custom table, another table will appear on the right reflecting the data you just entered.

Data Labels

Name of Control

C

Name of Treatment

T

	File.Name	Sample.Group	Replicate	Experimental.Group	Custom.ID
1	Reporter.intensity.corrected.0	Nuc	1	T	NA_T_1
2	Reporter.intensity.corrected.1	Nuc	2	T	NA_T_2
3	Reporter.intensity.corrected.2	Nuc	3	T	NA_T_3
4	Reporter.intensity.corrected.3	Nuc	1	C	NA_1
5	Reporter.intensity.corrected.4	Nuc	2	C	NA_2
6	Reporter.intensity.corrected.5	Nuc	3	C	NA_3

Update Data

Show 10 entries

Search:

	File.Name	Sample.Group	Replicate	Experimental.Group	Custom.ID
1	Reporter.intensity.corrected.0	Nuc	1	T	NA_T_1
2	Reporter.intensity.corrected.1	Nuc	2	T	NA_T_2
3	Reporter.intensity.corrected.2	Nuc	3	T	NA_T_3
4	Reporter.intensity.corrected.3	Nuc	1	C	NA_1
5	Reporter.intensity.corrected.4	Nuc	2	C	NA_2
6	Reporter.intensity.corrected.5	Nuc	3	C	NA_3

Showing 1 to 6 of 6 entries

Previous 1 Next

Protein: Preliminary

- This tab contains figures that can be used for assessing the quality of your data.
- Figures
  - Histograms
  - Boxplots
  - PCA/ MDS

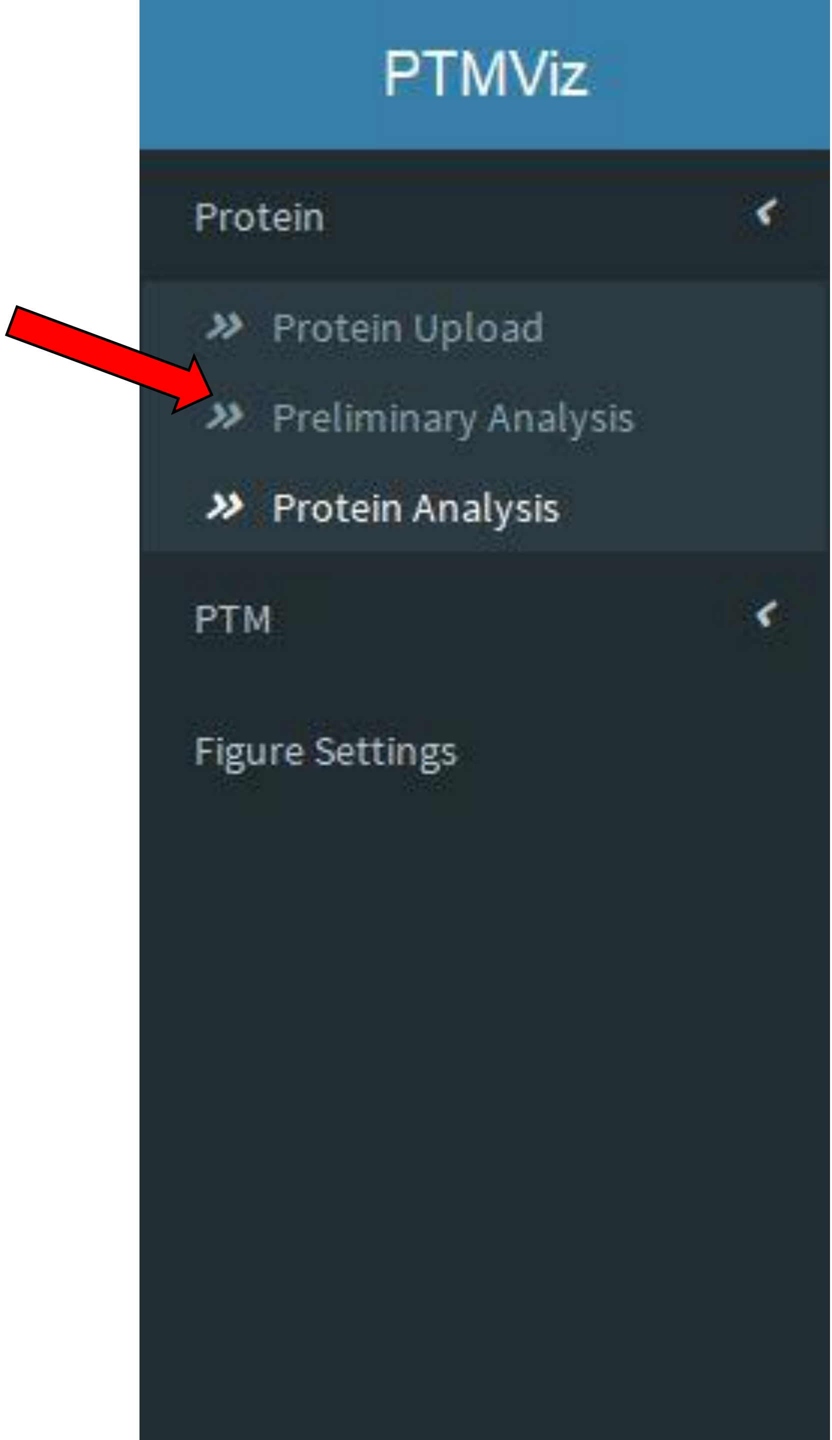

Protein: Preliminary

- The first tab on all figures will contain the actual figures
- The second tab will usually be for settings. This will include options for labels, text sizes and colors.
- The bottom of the figure will have download button for saving the figure

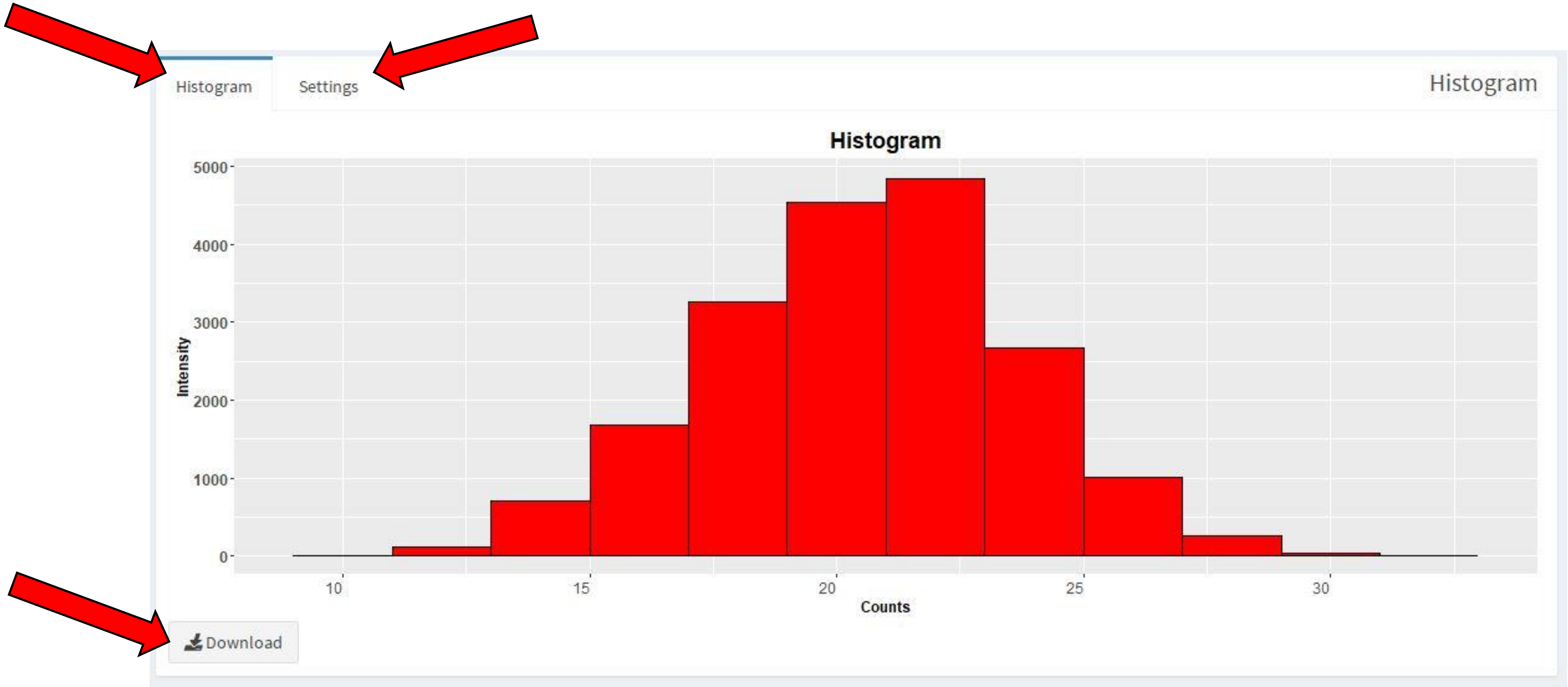

Protein: Protein Analysis

- This section will contain main differential analysis and comparisons in the data
- Figures:
  - Volcano Plot
  - Heatmap

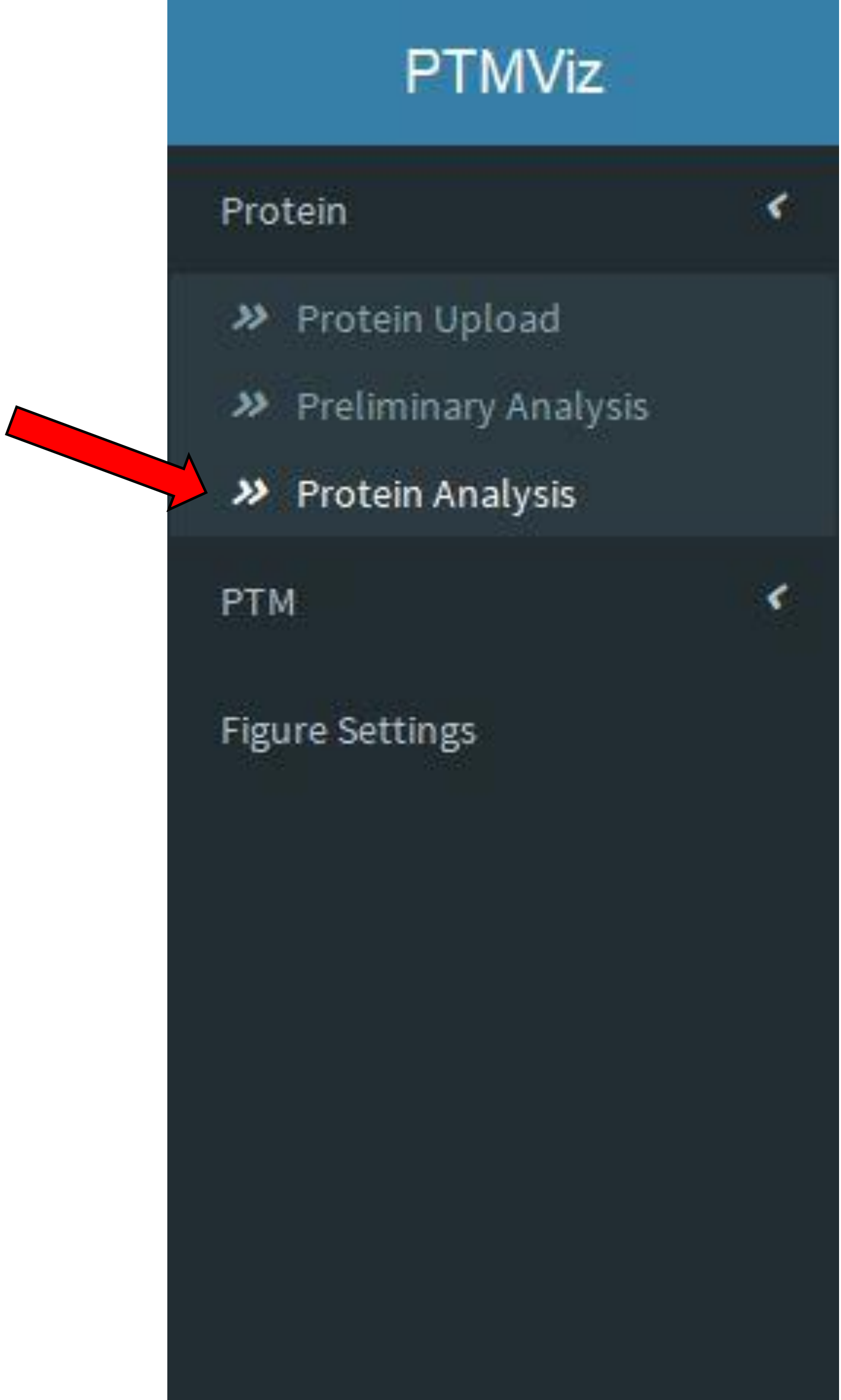

Protein Analysis: Data Table

- There are 4 data tables in the protein analysis tab
  - The All Data tab has the differential analysis of every protein in the data
  - The Significant Data tab has only statistically significant differentially expressed proteins
  - The All Intensity Data tab has all the raw data values
  - The Significant Intensity Data is raw data values based on the significant data tab
- Each table can copied to the clipboard or downloaded as a csv or pdf

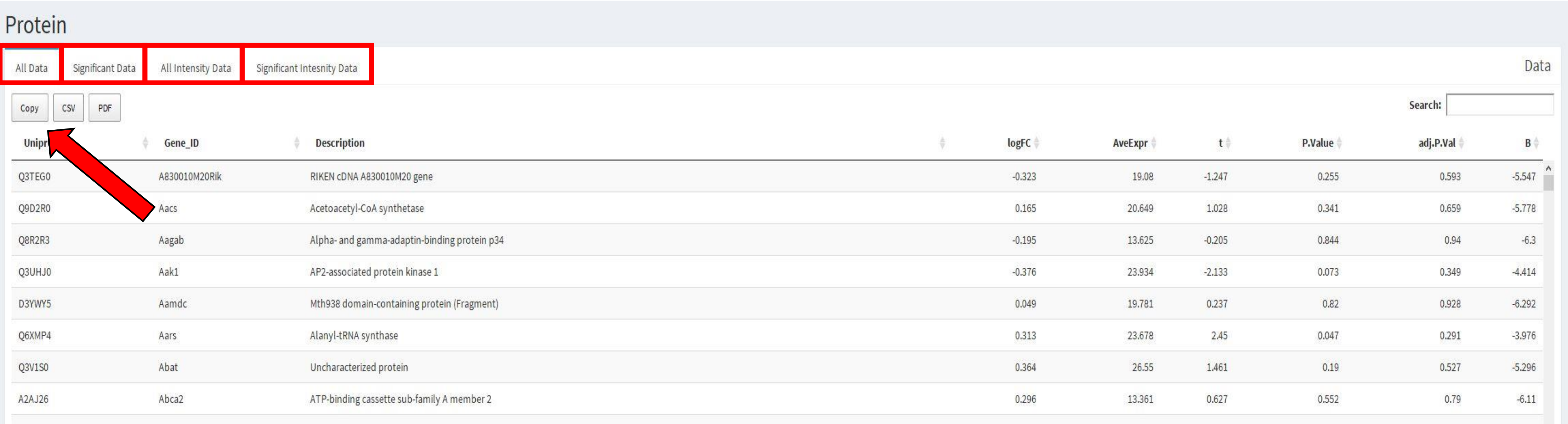

Unipr	Gene_ID	Description	logFC	AveExpr	t	P.Value	adj.P.Val	B
Q3TEG0	A830010M20Rik	RIKEN cDNA A830010M20 gene	-0.323	19.08	-1.247	0.255	0.593	-5.547
Q9D2R0	Aacs	Acetoacetyl-CoA synthetase	0.165	20.649	1.028	0.341	0.659	-5.778
Q8R2R3	Aagab	Alpha- and gamma-adaptin-binding protein p34	-0.195	13.625	-0.205	0.844	0.94	-6.3
Q3UHJ0	Aak1	AP2-associated protein kinase 1	-0.376	23.934	-2.133	0.073	0.349	-4.414
D3YWY5	Aamdc	Mth938 domain-containing protein (Fragment)	0.049	19.781	0.237	0.82	0.928	-6.292
Q6XMP4	Aars	Alanyl-tRNA synthase	0.313	23.678	2.45	0.047	0.291	-3.976
Q3V1S0	Abat	Uncharacterized protein	0.364	26.55	1.461	0.19	0.527	-5.296
A2AJ26	Abca2	ATP-binding cassette sub-family A member 2	0.296	13.361	0.627	0.552	0.79	-6.11

Protein Analysis: Volcano Plot

- The Volcano plot allows you to hover your mouse over points to reveal their ID and values
- Selecting a group of points using the rectangle or lasso select option in the top right corner will isolate the points in the table above the volcano plot

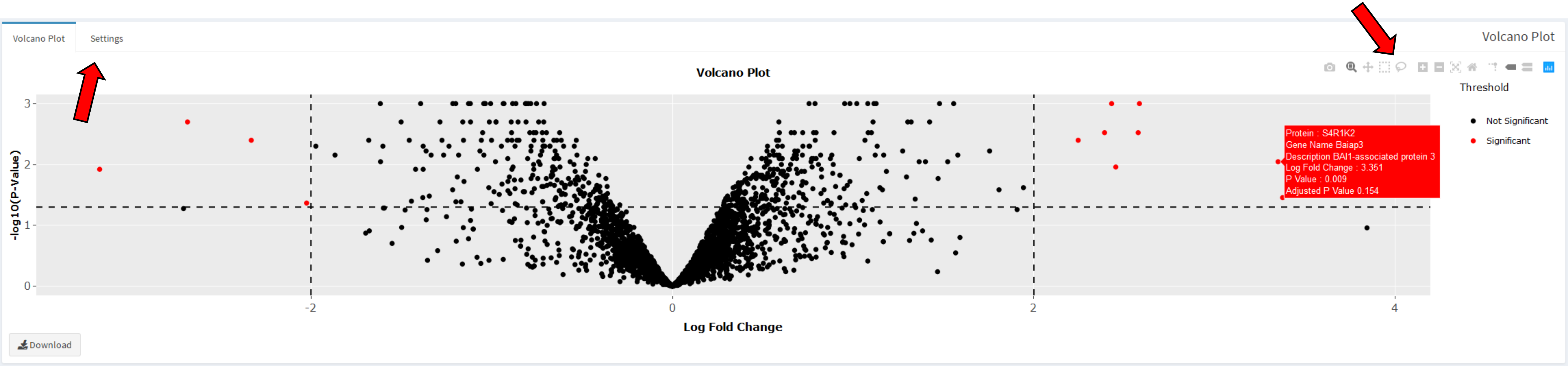

Protein Analysis: Volcano Plot Settings

- The Settings for the Volcano Plot include color and label options
- Additionally, the user can set the fold change and p value threshold for what the graph considers statistically significant (Changing these values will also affect the individuals in the data table and heatmap)
- You can also select whether the tool is using the normal p value or an adjected p value

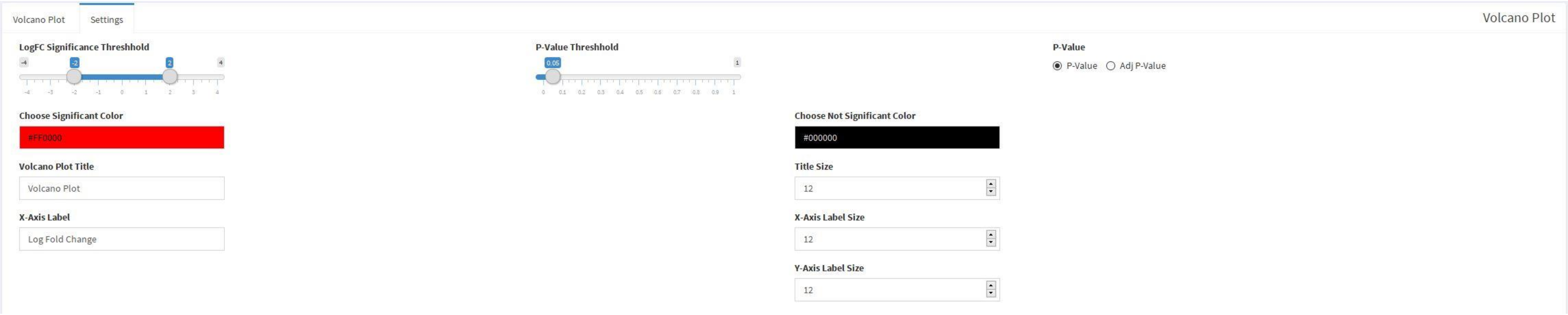

Protein Analysis: Heatmap

- The Heatmap at the bottom will display all the significant proteins in the data (as are determined in the volcano plot)
- Hovering over the tiles will reveal its exact value, similar to the volcano plot

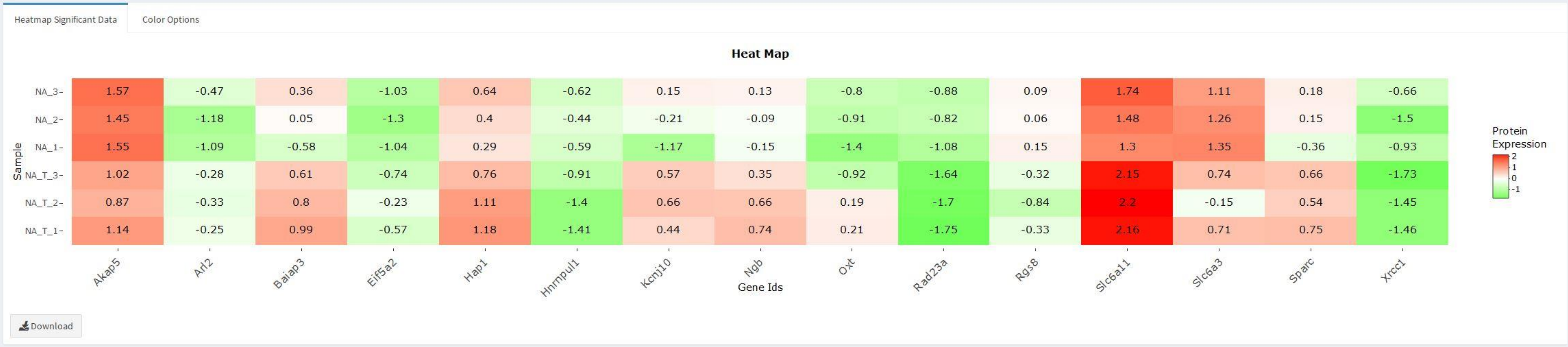

Protein Analysis: Heatmap Color Options

- Within the heatmap color options you can change what color represents the high, low and mid ranges
- Here you can also turn on or off the numeric labels within each tile and wether you want the data to be scaled or not

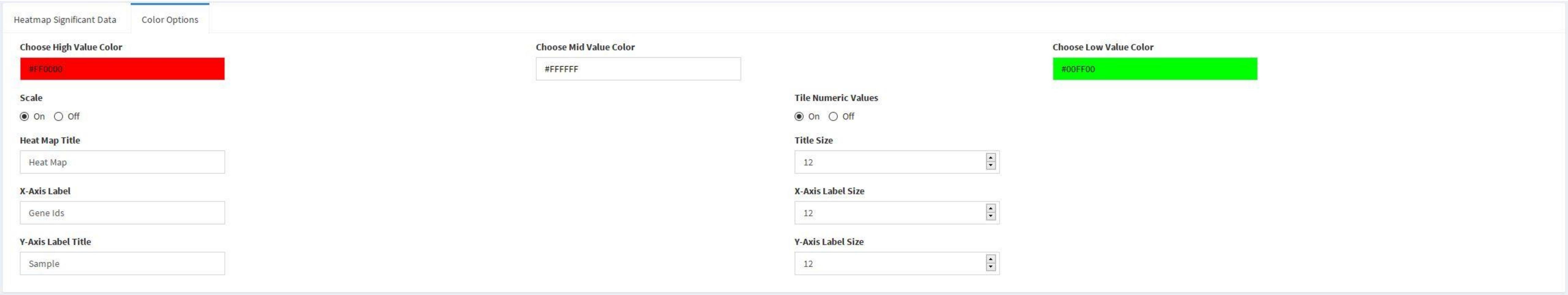

PTM: PTM Analysis

- The PTM Analysis tab will contain all of the relevant figures and analysis of the PTM data

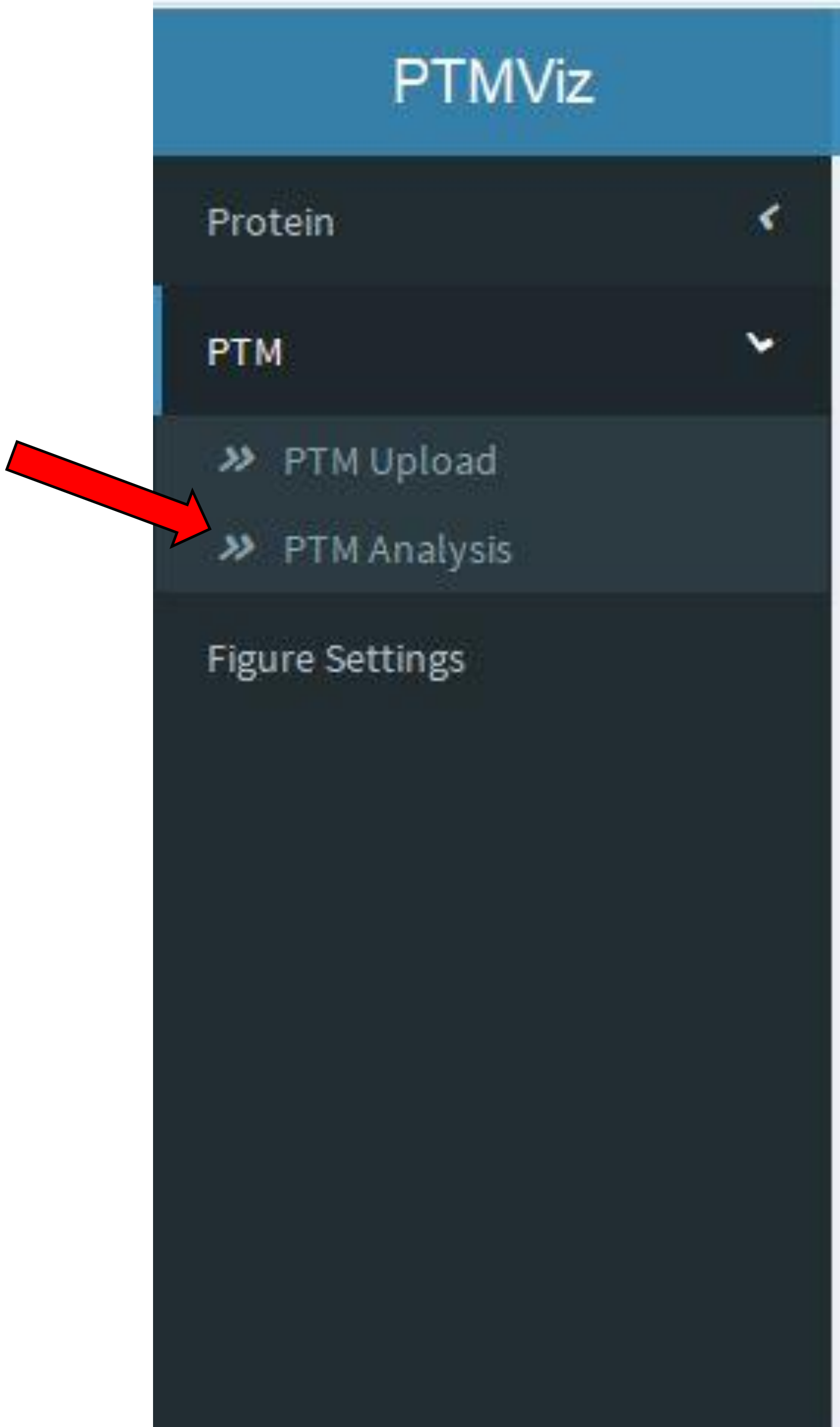

PTM Analysis: Data Table

- The data table represents a combination of the data and metadata that was uploaded
- At the top there are a set of filters that can narrow down the data based on the sample group, replicate, specific histones, and/or PTM Residue
- Below that are the same copy or download options that are available for the protein data table

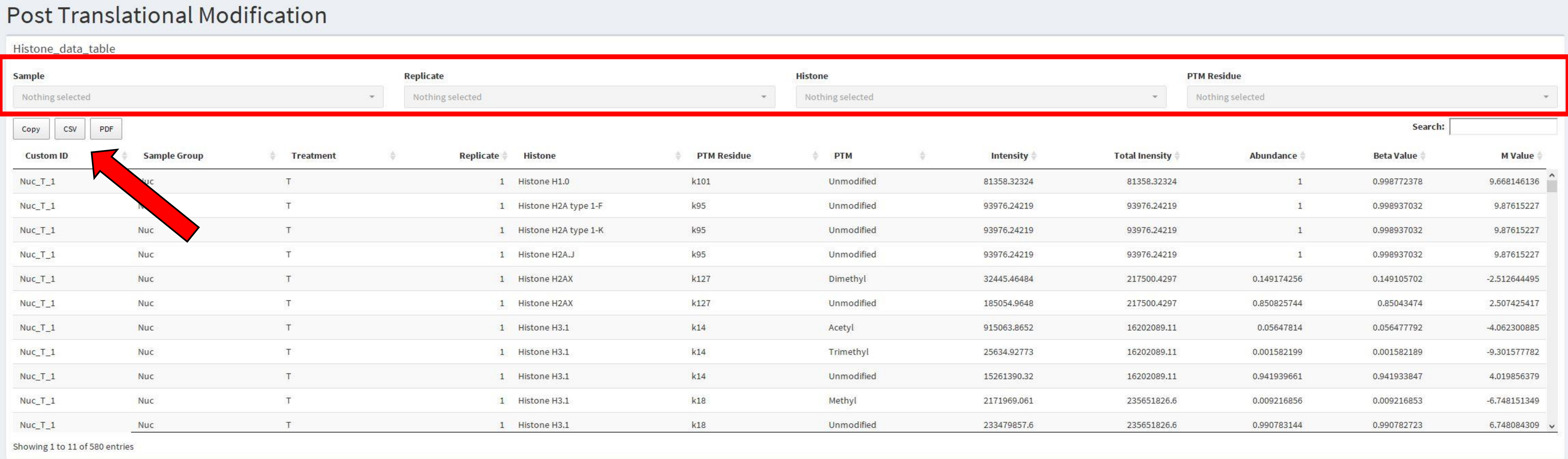

Custom ID	Sample Group	Treatment	Replicate	Histone	PTM Residue	PTM	Intensity	Total Inensity	Abundance	Beta Value	M Value
Nuc_T_1	[illegible]	T	1	Histone H1.0	k101	Unmodified	81358.32324	81358.32324	1	0.998772378	9.668146136
Nuc_T_1	[illegible]	T	1	Histone H2A type 1-F	k95	Unmodified	93976.24219	93976.24219	1	0.998937032	9.87615227
Nuc_T_1	Nuc	T	1	Histone H2A type 1-K	k95	Unmodified	93976.24219	93976.24219	1	0.998937032	9.87615227
Nuc_T_1	Nuc	T	1	Histone H2A.J	k95	Unmodified	93976.24219	93976.24219	1	0.998937032	9.87615227
Nuc_T_1	Nuc	T	1	Histone H2AX	k127	Dimethyl	32445.46484	217500.4297	0.149174256	0.149105702	-2.512644495
Nuc_T_1	Nuc	T	1	Histone H2AX	k127	Unmodified	185054.9648	217500.4297	0.850825744	0.85043474	2.507425417
Nuc_T_1	Nuc	T	1	Histone H3.1	k14	Acetyl	915063.8652	16202089.11	0.05647814	0.056477792	-4.062300885
Nuc_T_1	Nuc	T	1	Histone H3.1	k14	Trimethyl	25634.92773	16202089.11	0.001582199	0.001582189	-9.301577782
Nuc_T_1	Nuc	T	1	Histone H3.1	k14	Unmodified	15261390.32	16202089.11	0.941939661	0.941933847	4.019856379
Nuc_T_1	Nuc	T	1	Histone H3.1	k18	Methyl	2171969.061	235651826.6	0.009216856	0.009216853	-6.748151349
Nuc_T_1	Nuc	T	1	Histone H3.1	k18	Unmodified	233479857.6	235651826.6	0.990783144	0.990782723	6.748084309

Showing 1 to 11 of 580 entries

PTM Analysis: Barchart

- At the top of the stacked barchart you can choose which information is displayed. Here you can filter out certain sample groups, whether you want to see the control and treatment at the same time, the exact PTM residue and the exact histone
- Below this there is a check box for viewing individual samples. Checking this will show each sample instead of an average of the samples
- The Settings tab contains adjustments for label text and sizes

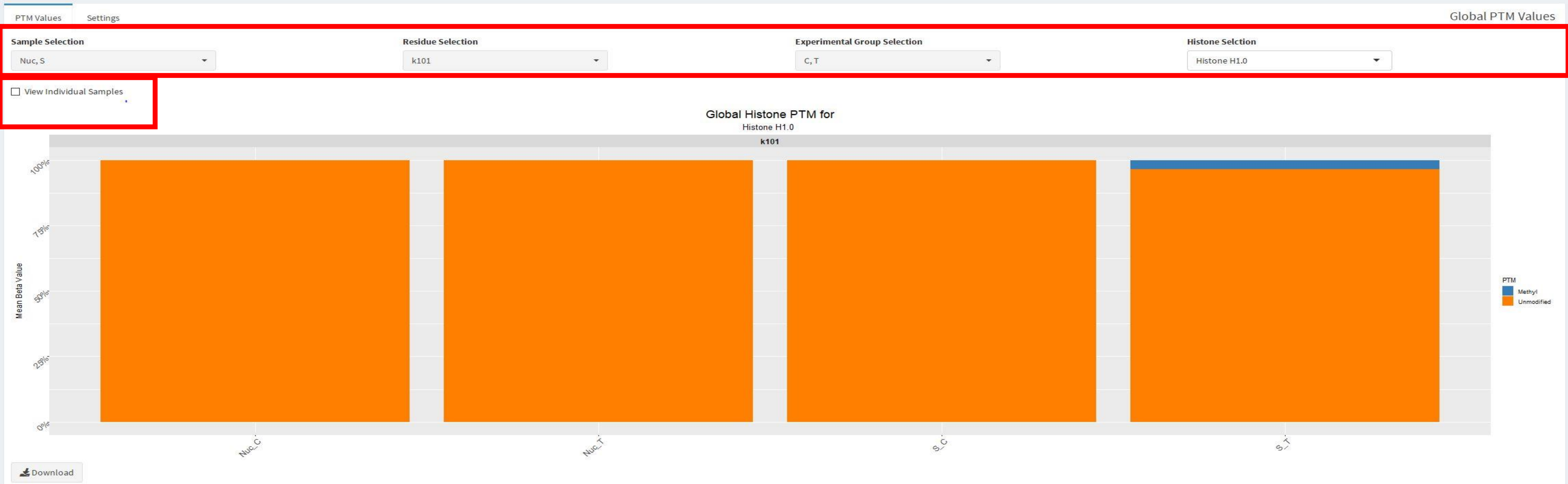

PTM Analysis: Differential Analysis

- The first tab of the differential analysis lets you choose which values to consider the treatment and which to consider the control. This will let you compare samples from two different sample groups if needed.

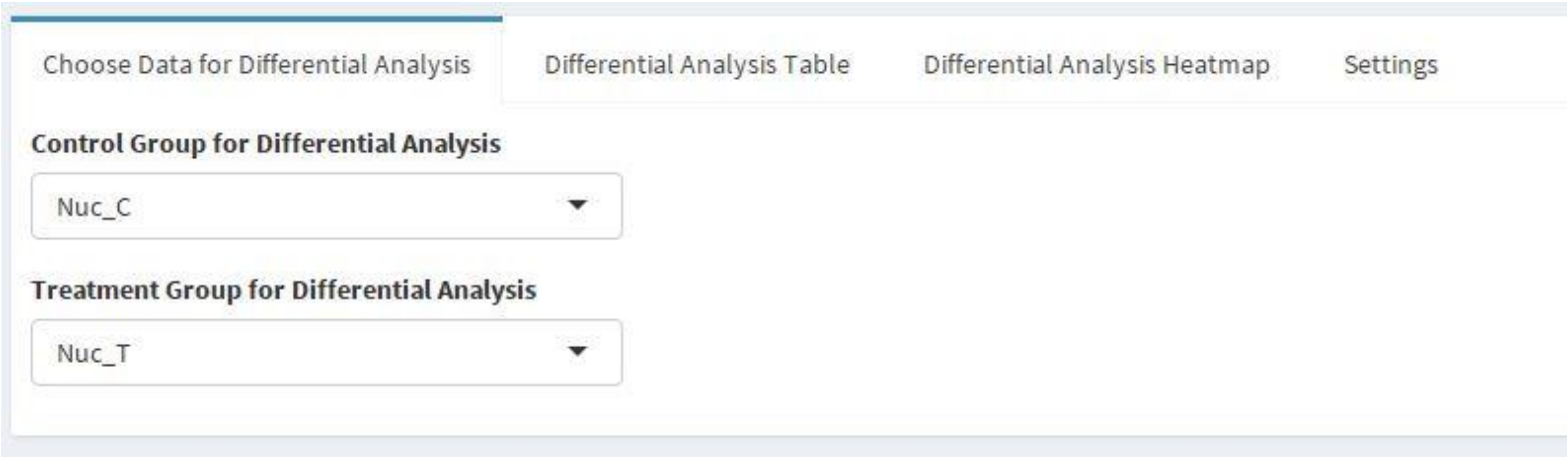

PTM Analysis: Differential Analysis

- The next tab displays the results of the differential analysis showing the fold change, false discovery rate and p value alongside the beta values of each sample
- This table can be downloaded like all the other data tables

Choose Data for Differential Analysis | Differential Analysis Table | Differential Analysis Heatmap | Settings

Differential Analysis

Copy | CSV | PDF

Search:

	Fold Change	pVal	FDR	Nuc_T_1	Nuc_T_2	Nuc_1	Nuc_2	Nuc_3
H3.1|H3.3|H3.3C k14 Acetyl	-6.815	0.006	0.043	0.056	0.037	0.906	0.934	0.534
H3.1|H3.3|H3.3C k9 Trimethyl	3.81	0.122	0.421	0.034	0.105	0.005		
H3.1|H3.3|H3.3C k9 Methyl	-1.225	0.242	0.421	0.655	0.65	0.715	0.88	0.821
H3.1|H3.3|H3.3C k9 Dimethyl	1.318	0.3	0.421	0.239	0.191	0.217	0.042	0.097
H4 k16 Acetyl	-6.685	0.301	0.421		0.05	0.428	0.211	0.999
H3.1|H3.3|H3.3C k79 Methyl	-0.449	0.775	0.904	0.105	0.138	0.158		
H3.1|H3.3 k18 Methyl	0.134	0.909	0.909	0.009	0.013	0.022	0.004	0.012
H2AX k127 Dimethyl				0.149				
H3.1 k27 Methyl				1	0.844			
H3.1 k27 Dimethyl					0.053			
H3.1 k27 Trimethyl					0.07			

Showing 1 to 11 of 13 entries

PTM Analysis: Differential Analysis

- The beta values can also be demonstrated as a heatmap for a more visual comparison of each value.
- The Settings tab will allow you to change the colors and labels similar to the heatmap in the protein analysis tab

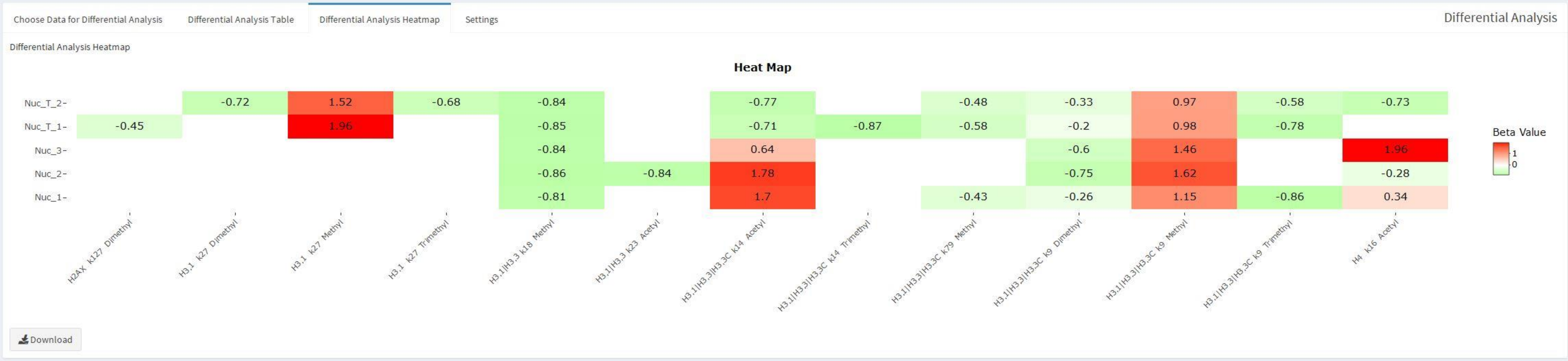

Figure Settings

- The figure settings will let you change how all the figures are saved when you hit the “Download” button beneath each figure

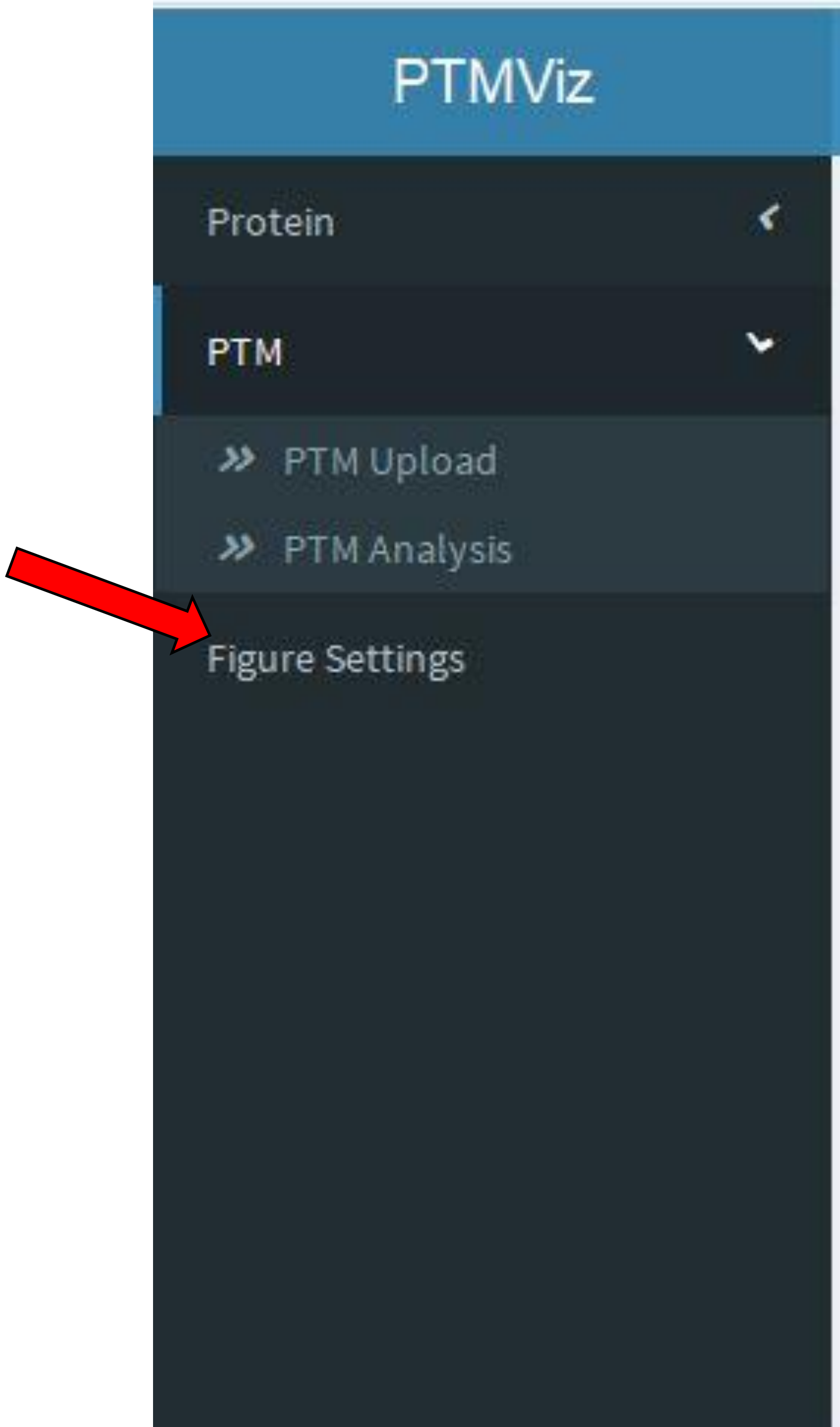

Figure Settings

- Selecting the file type will all the download buttons to save figures to what is selected
- The size of the image can also be altered to better fit wider figures
- The resolution of each figure can also be set with the dpi

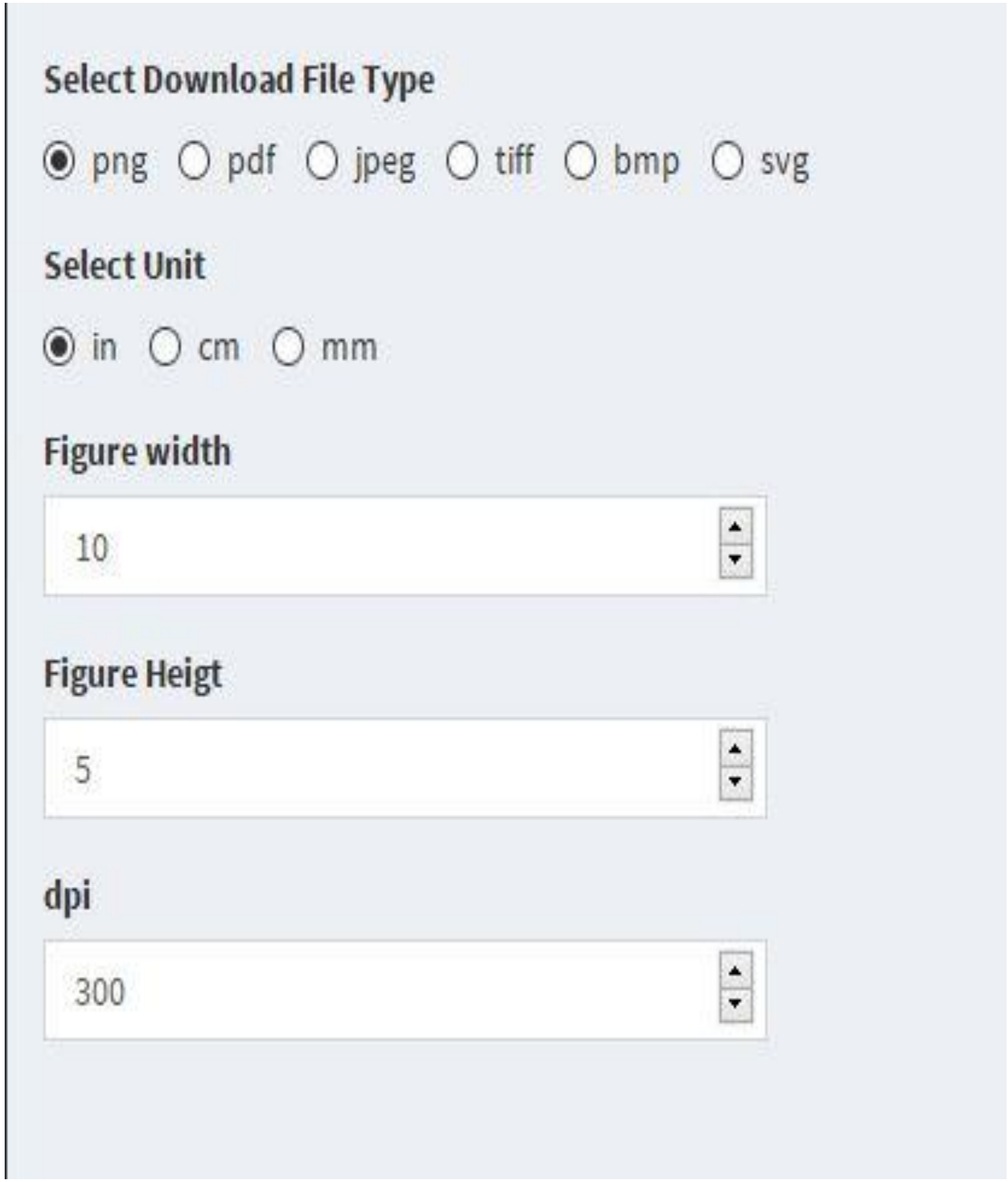

Supplement: Supplementary file 1 — Additional file 1. The data and PTMviz scripts. [file 12859_2021_4166_MOESM1_ESM.zip › Read Me.pdf]
