# Supplementary material for: PTMViz: a tool for analyzing and visualizing histone post translational modification data
Source: BMC Bioinformatics. 2021 May 26;22:275. doi: 10.1186/s12859-021-04166-9 (PMC8157737; doi:10.1186/s12859-021-04166-9)
Supplement: Supplementary file 1 — Additional file 1. The data and PTMviz scripts. [file 12859_2021_4166_MOESM1_ESM.zip › Read Me.pptx]

## Slide 1
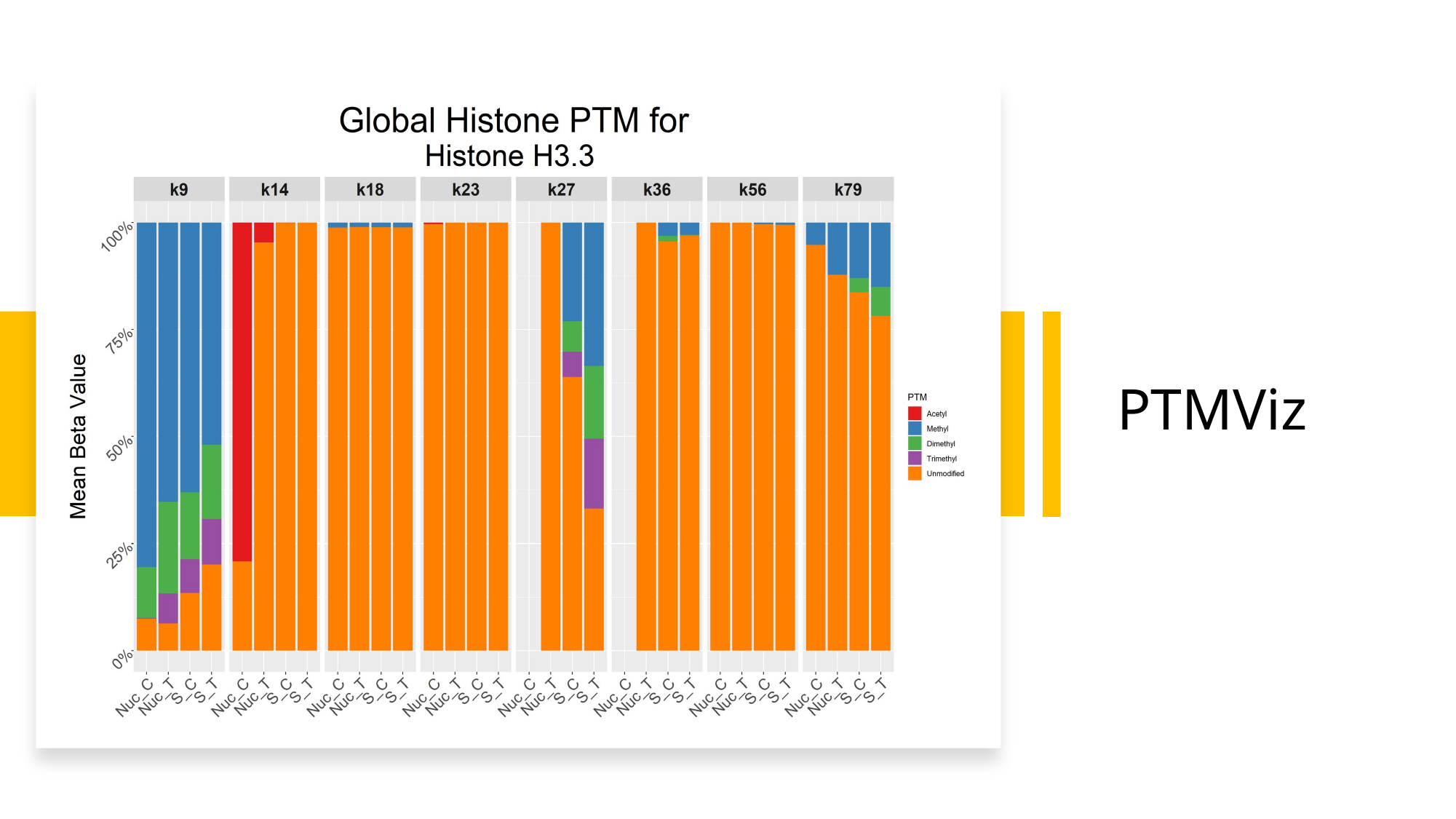

# PTMViz

## Slide 2
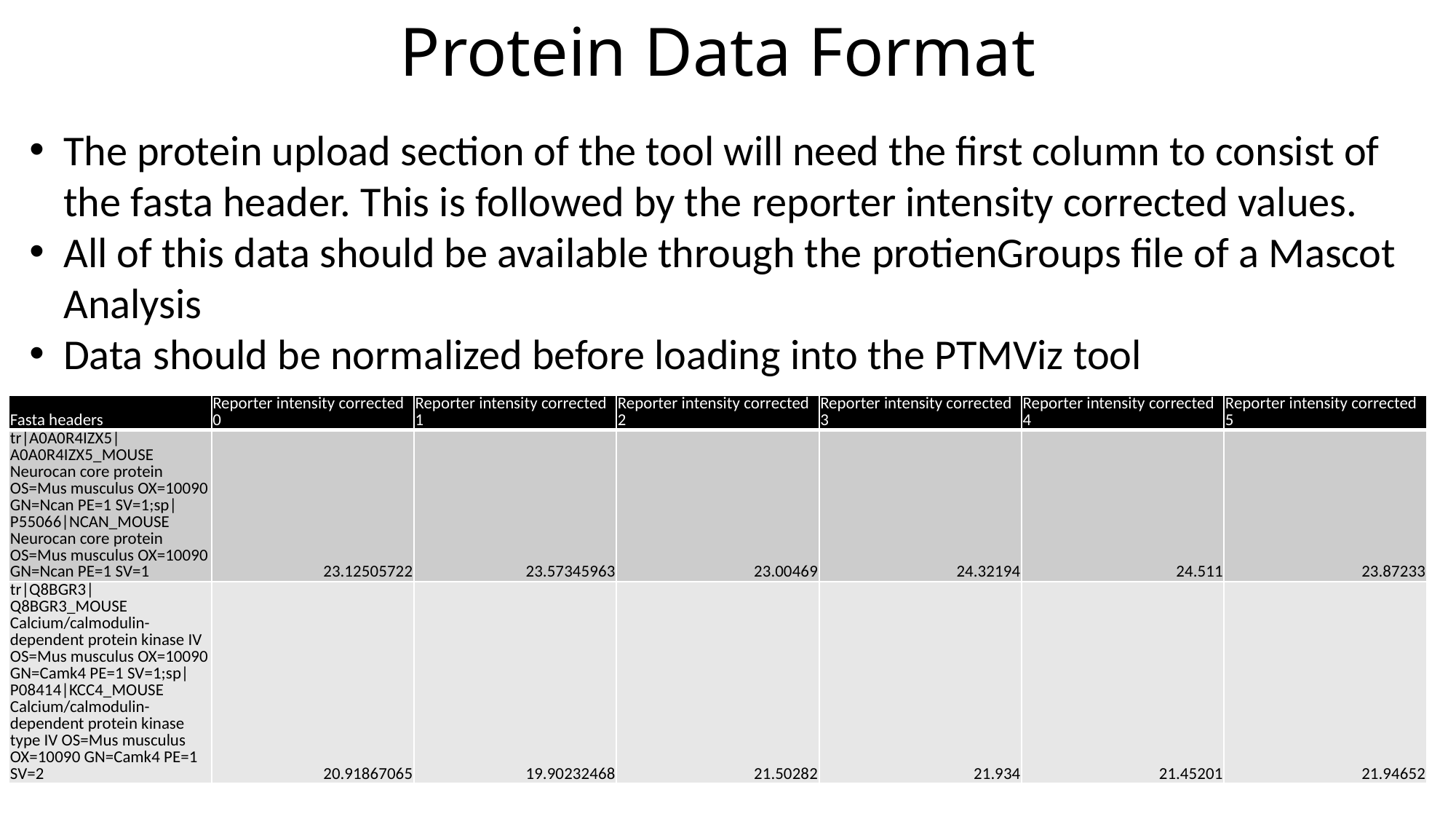

# Protein Data Format
The protein upload section of the tool will need the first column to consist of the fasta header. This is followed by the reporter intensity corrected values.
All of this data should be available through the protienGroups file of a Mascot Analysis
Data should be normalized before loading into the PTMViz tool
| Fasta headers | Reporter intensity corrected 0 | Reporter intensity corrected 1 | Reporter intensity corrected 2 | Reporter intensity corrected 3 | Reporter intensity corrected 4 | Reporter intensity corrected 5 |
| --- | --- | --- | --- | --- | --- | --- |
| tr|A0A0R4IZX5|A0A0R4IZX5\_MOUSE Neurocan core protein OS=Mus musculus OX=10090 GN=Ncan PE=1 SV=1;sp|P55066|NCAN\_MOUSE Neurocan core protein OS=Mus musculus OX=10090 GN=Ncan PE=1 SV=1 | 23.12505722 | 23.57345963 | 23.00469 | 24.32194 | 24.511 | 23.87233 |
| tr|Q8BGR3|Q8BGR3\_MOUSE Calcium/calmodulin-dependent protein kinase IV OS=Mus musculus OX=10090 GN=Camk4 PE=1 SV=1;sp|P08414|KCC4\_MOUSE Calcium/calmodulin-dependent protein kinase type IV OS=Mus musculus OX=10090 GN=Camk4 PE=1 SV=2 | 20.91867065 | 19.90232468 | 21.50282 | 21.934 | 21.45201 | 21.94652 |

## Slide 3
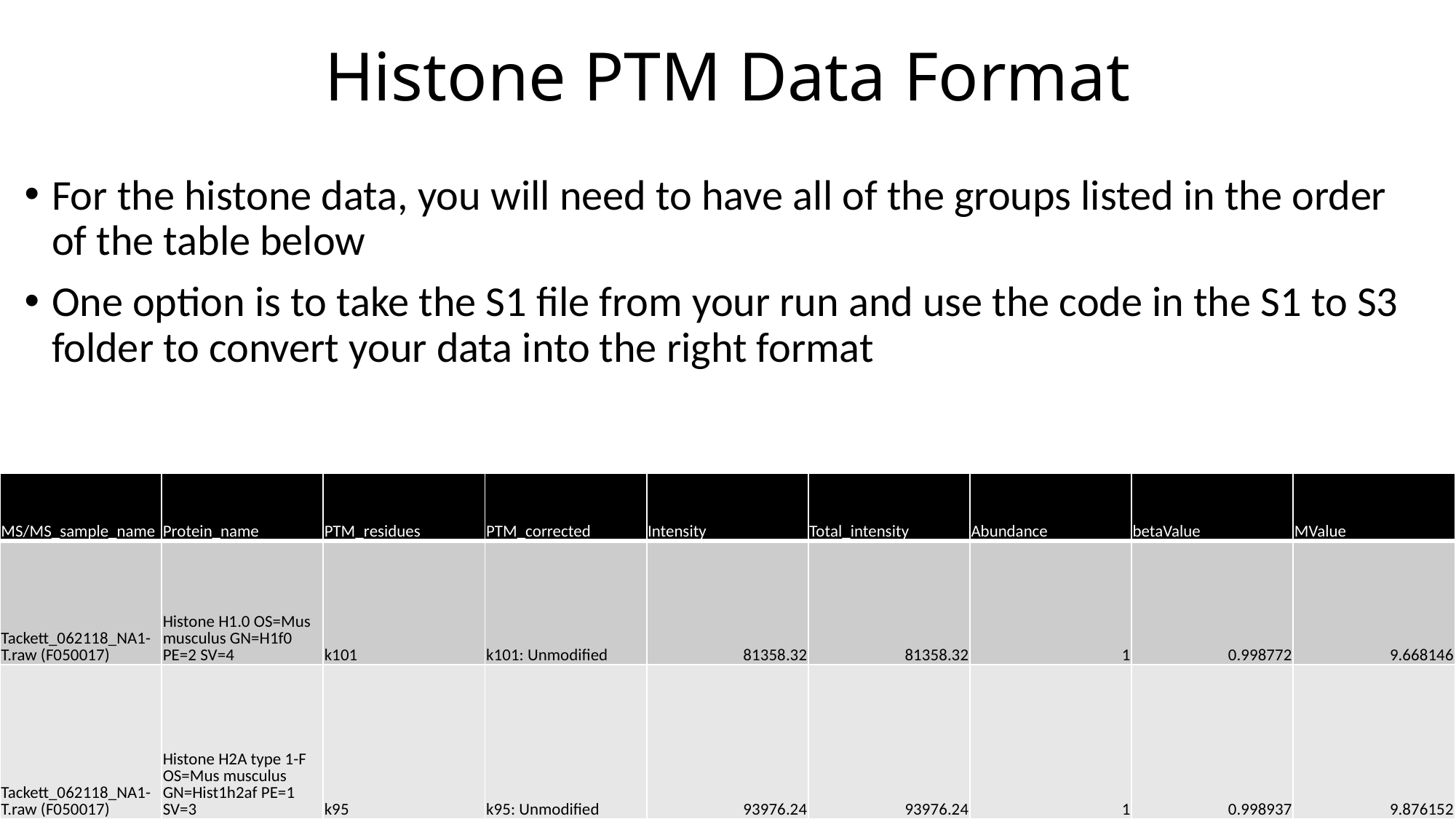

# Histone PTM Data Format
For the histone data, you will need to have all of the groups listed in the order of the table below
One option is to take the S1 file from your run and use the code in the S1 to S3 folder to convert your data into the right format
| MS/MS\_sample\_name | Protein\_name | PTM\_residues | PTM\_corrected | Intensity | Total\_intensity | Abundance | betaValue | MValue |
| --- | --- | --- | --- | --- | --- | --- | --- | --- |
| Tackett\_062118\_NA1-T.raw (F050017) | Histone H1.0 OS=Mus musculus GN=H1f0 PE=2 SV=4 | k101 | k101: Unmodified | 81358.32 | 81358.32 | 1 | 0.998772 | 9.668146 |
| Tackett\_062118\_NA1-T.raw (F050017) | Histone H2A type 1-F OS=Mus musculus GN=Hist1h2af PE=1 SV=3 | k95 | k95: Unmodified | 93976.24 | 93976.24 | 1 | 0.998937 | 9.876152 |

## Slide 4
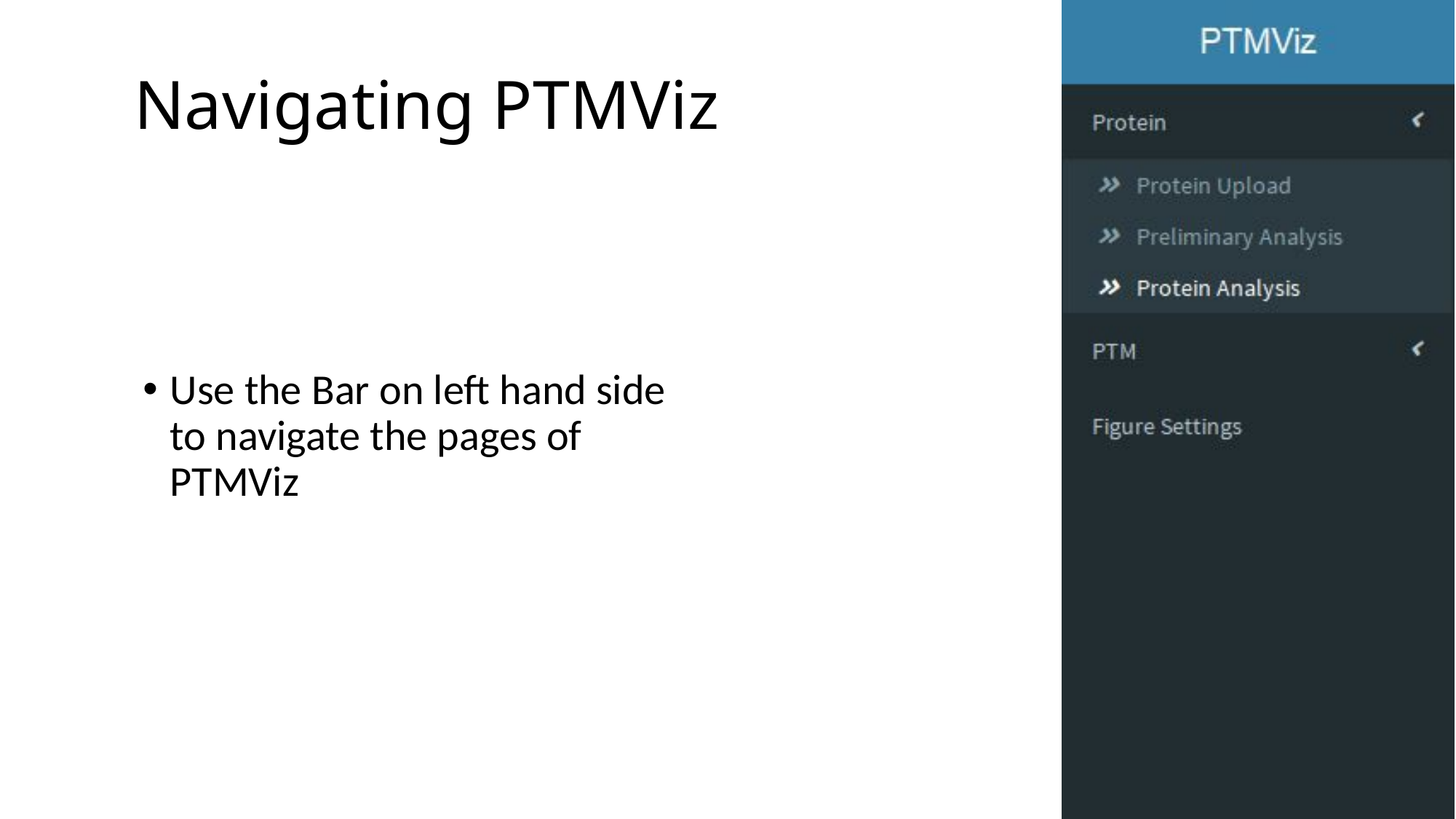

# Navigating PTMViz
Use the Bar on left hand side to navigate the pages of PTMViz

## Slide 5
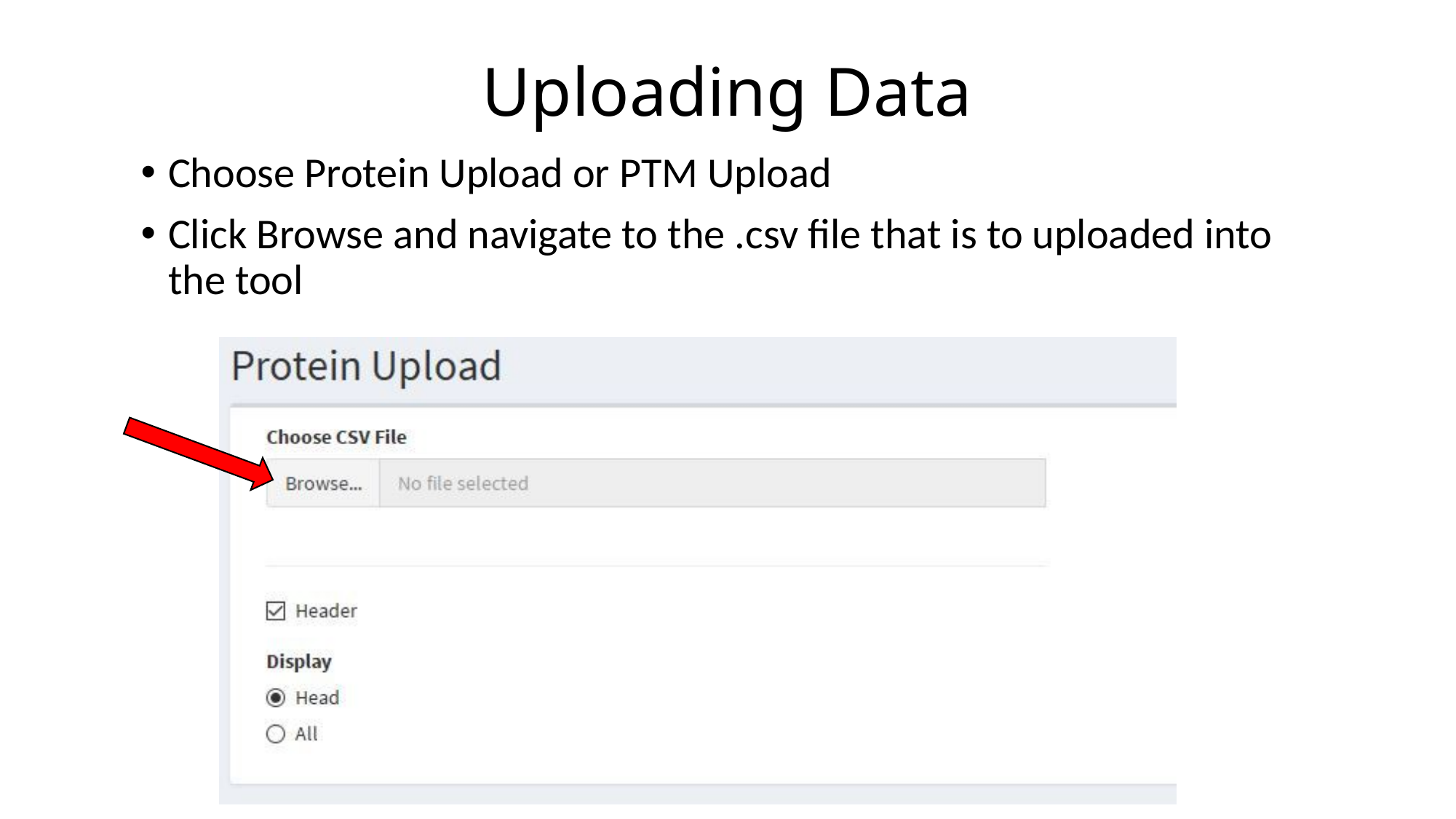

# Uploading Data
Choose Protein Upload or PTM Upload
Click Browse and navigate to the .csv file that is to uploaded into the tool

## Slide 6
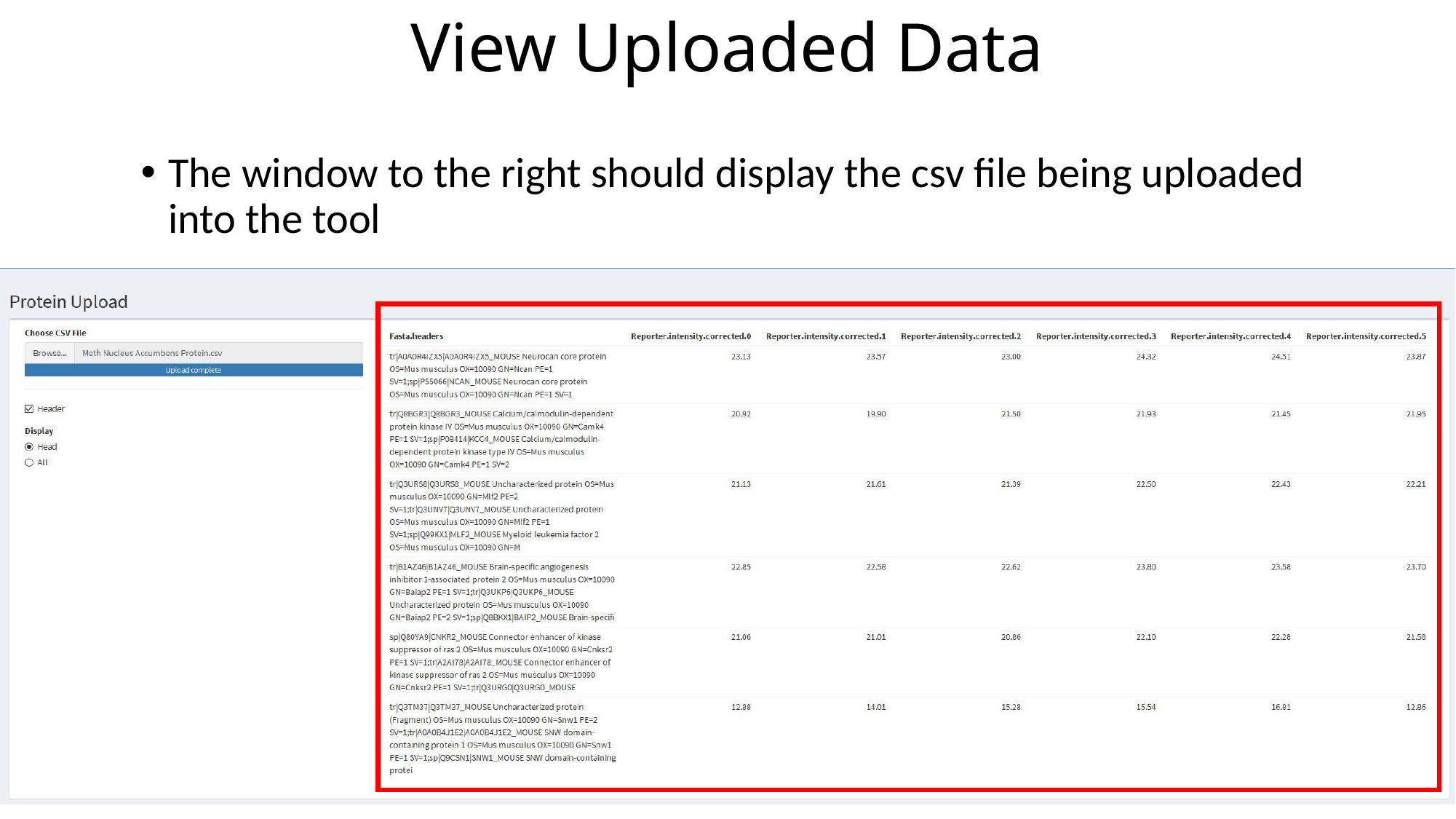

# View Uploaded Data
The window to the right should display the csv file being uploaded into the tool

## Slide 7
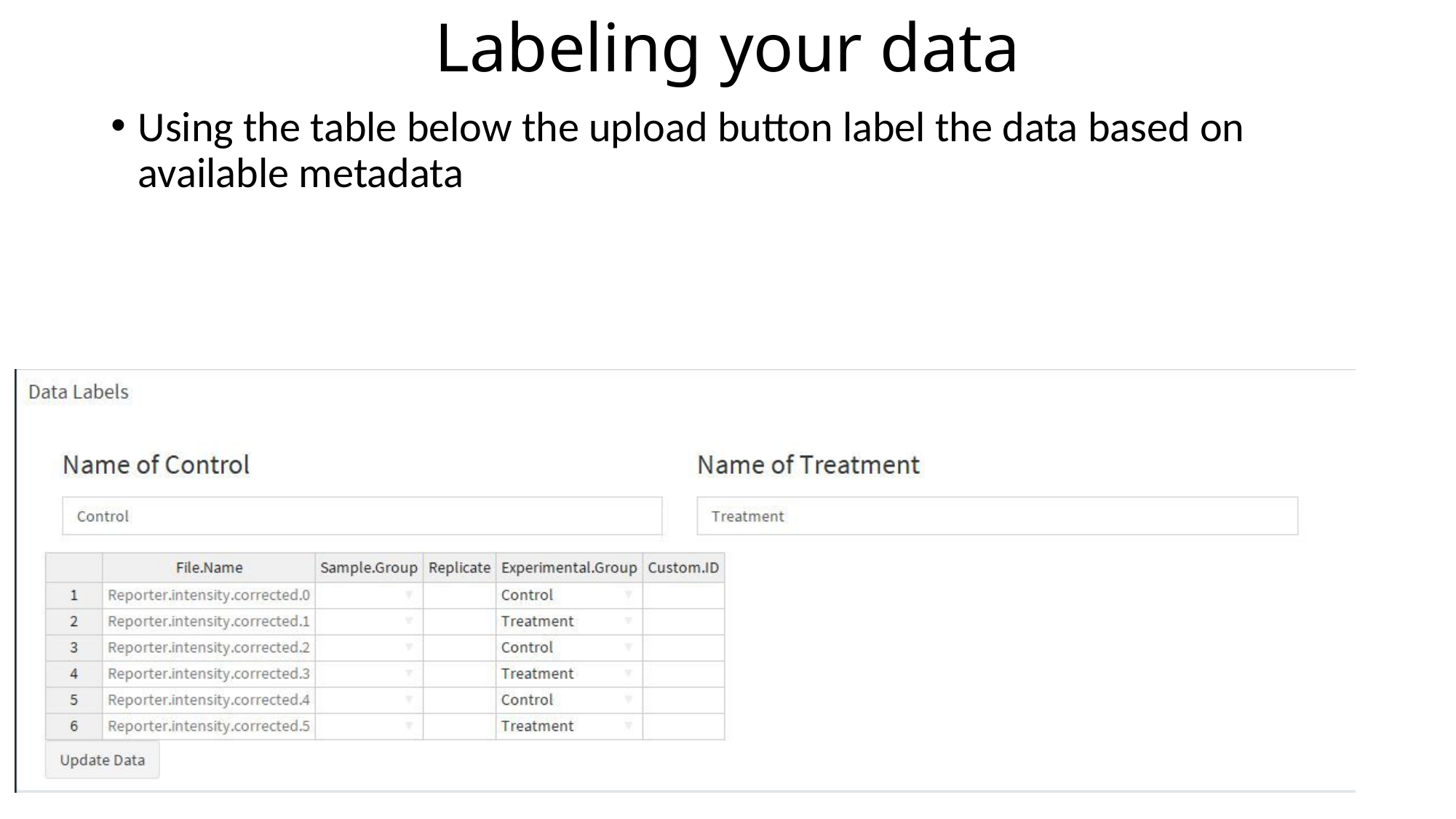

# Labeling your data
Using the table below the upload button label the data based on available metadata

## Slide 8
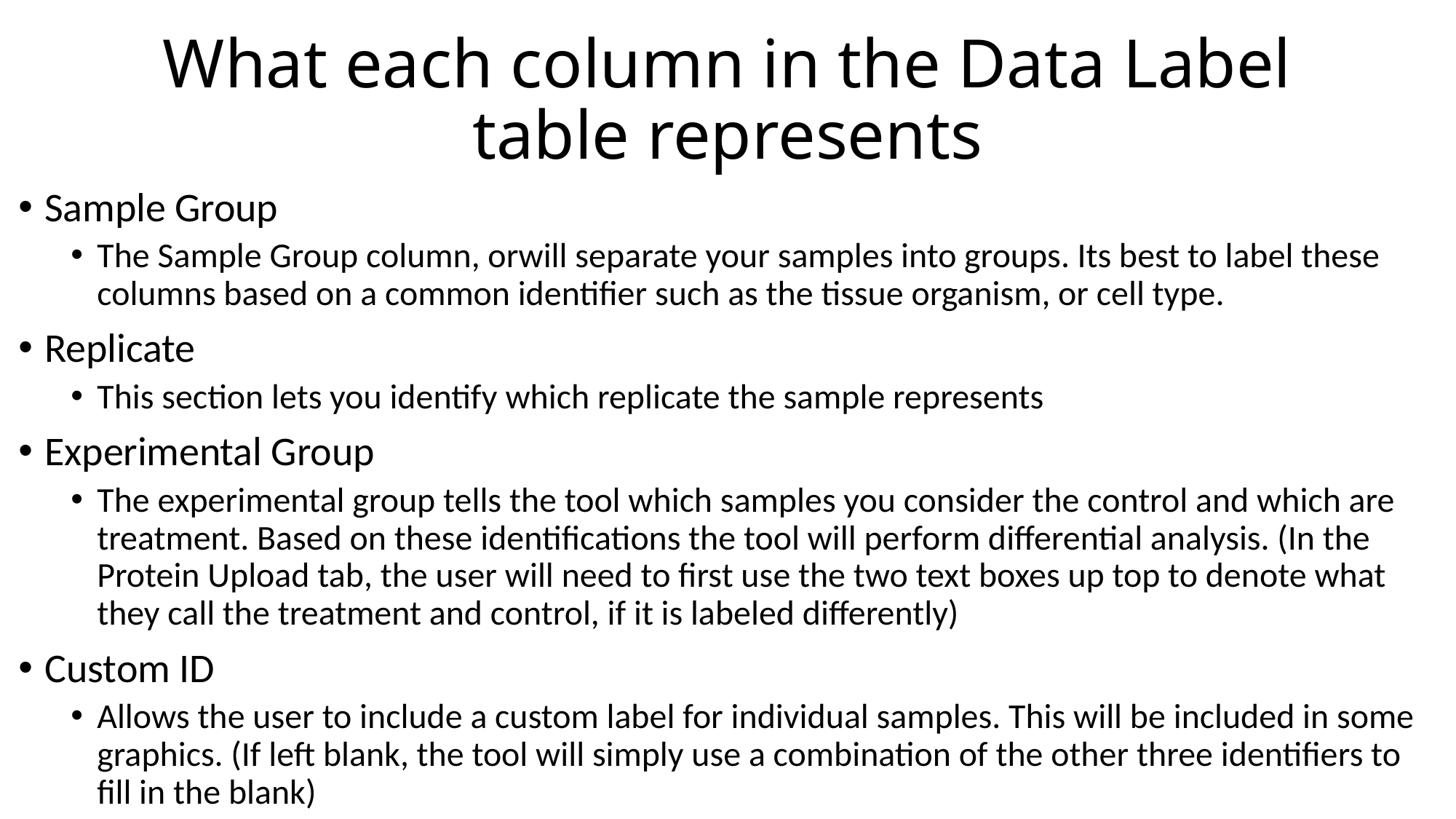

# What each column in the Data Label table represents
Sample Group
The Sample Group column, orwill separate your samples into groups. Its best to label these columns based on a common identifier such as the tissue organism, or cell type.
Replicate
This section lets you identify which replicate the sample represents
Experimental Group
The experimental group tells the tool which samples you consider the control and which are treatment. Based on these identifications the tool will perform differential analysis. (In the Protein Upload tab, the user will need to first use the two text boxes up top to denote what they call the treatment and control, if it is labeled differently)
Custom ID
Allows the user to include a custom label for individual samples. This will be included in some graphics. (If left blank, the tool will simply use a combination of the other three identifiers to fill in the blank)

## Slide 9
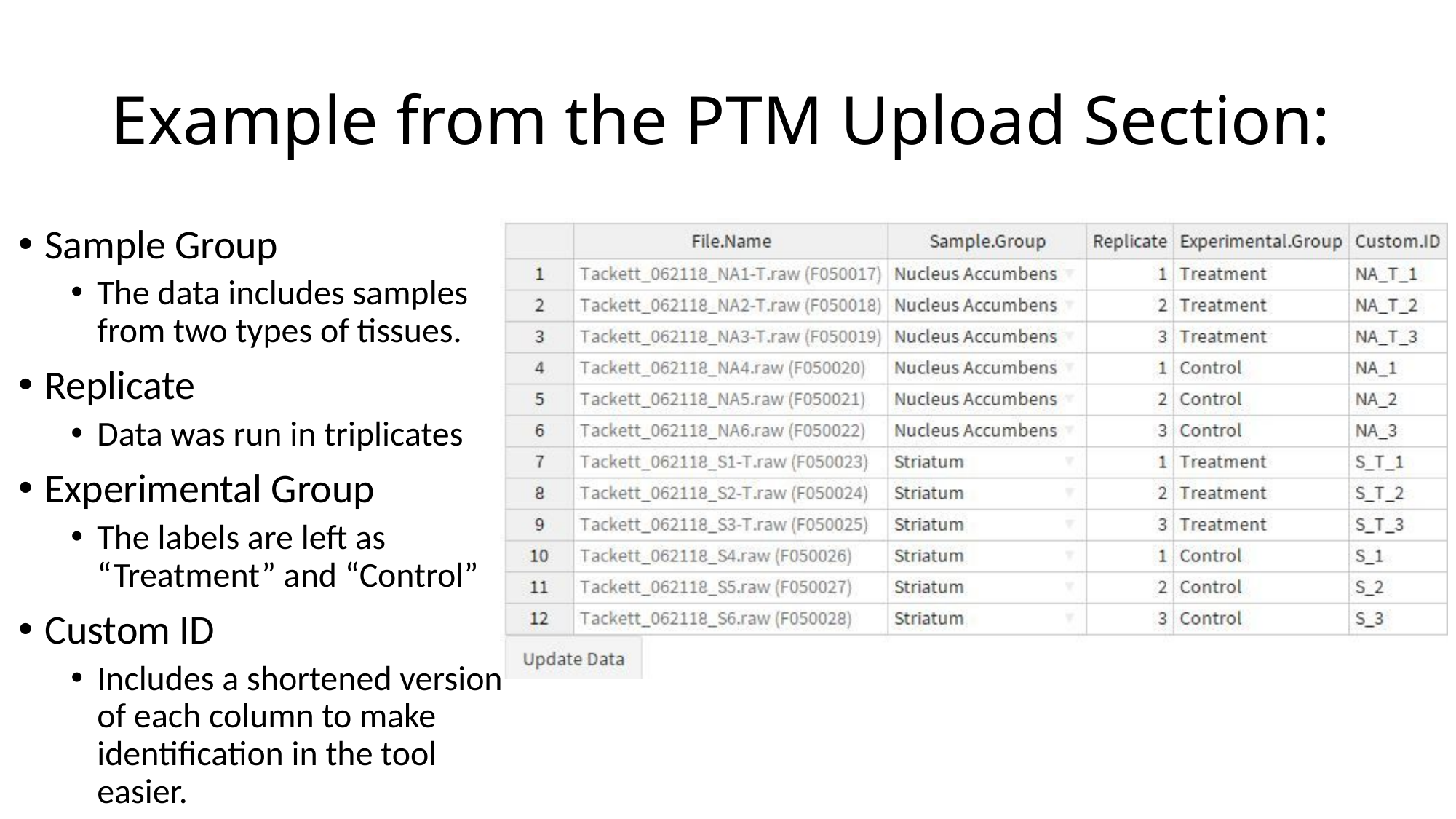

# Example from the PTM Upload Section:
Sample Group
The data includes samples from two types of tissues.
Replicate
Data was run in triplicates
Experimental Group
The labels are left as “Treatment” and “Control”
Custom ID
Includes a shortened version of each column to make identification in the tool easier.

## Slide 10
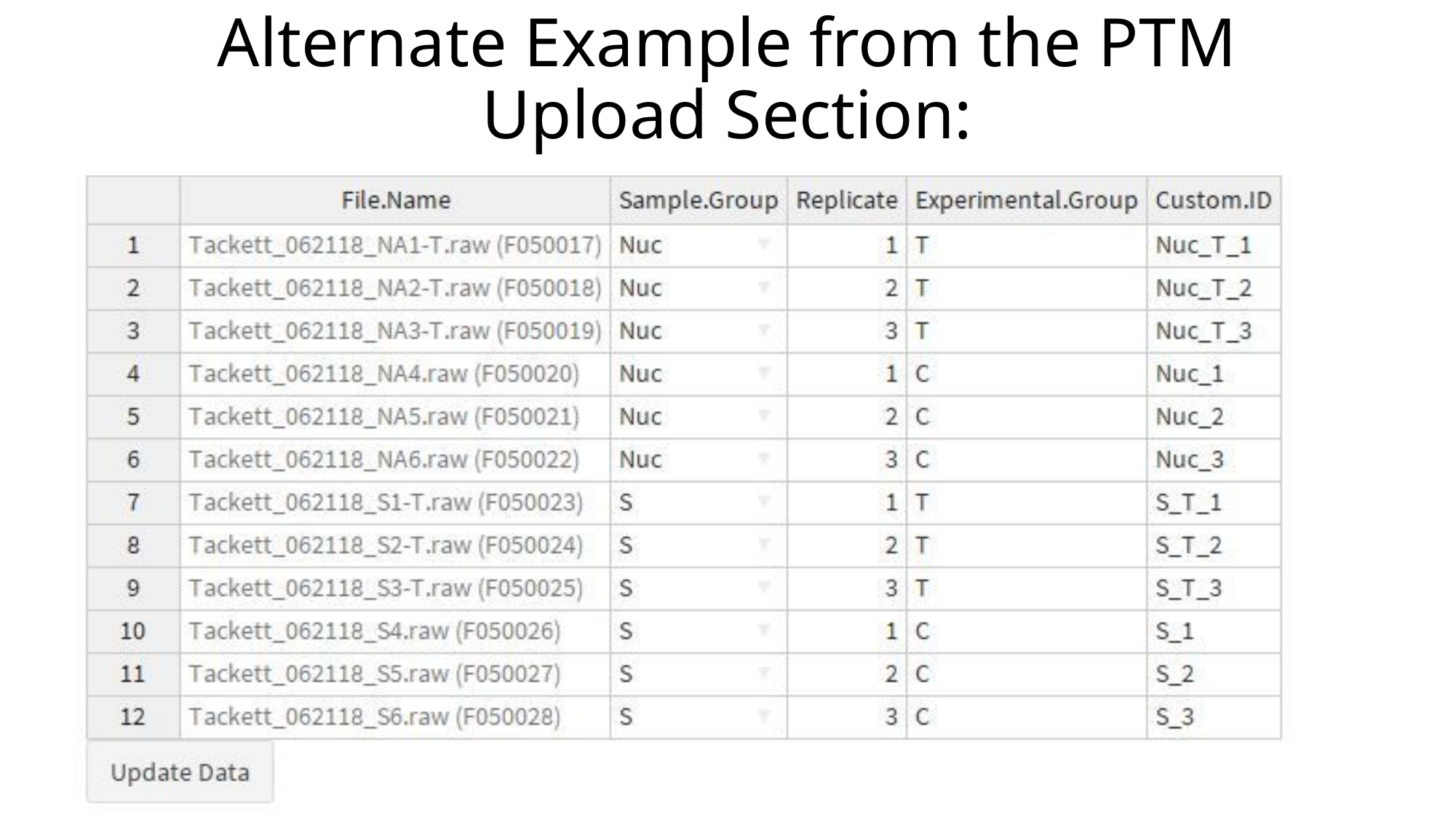

# Alternate Example from the PTM Upload Section:

## Slide 11
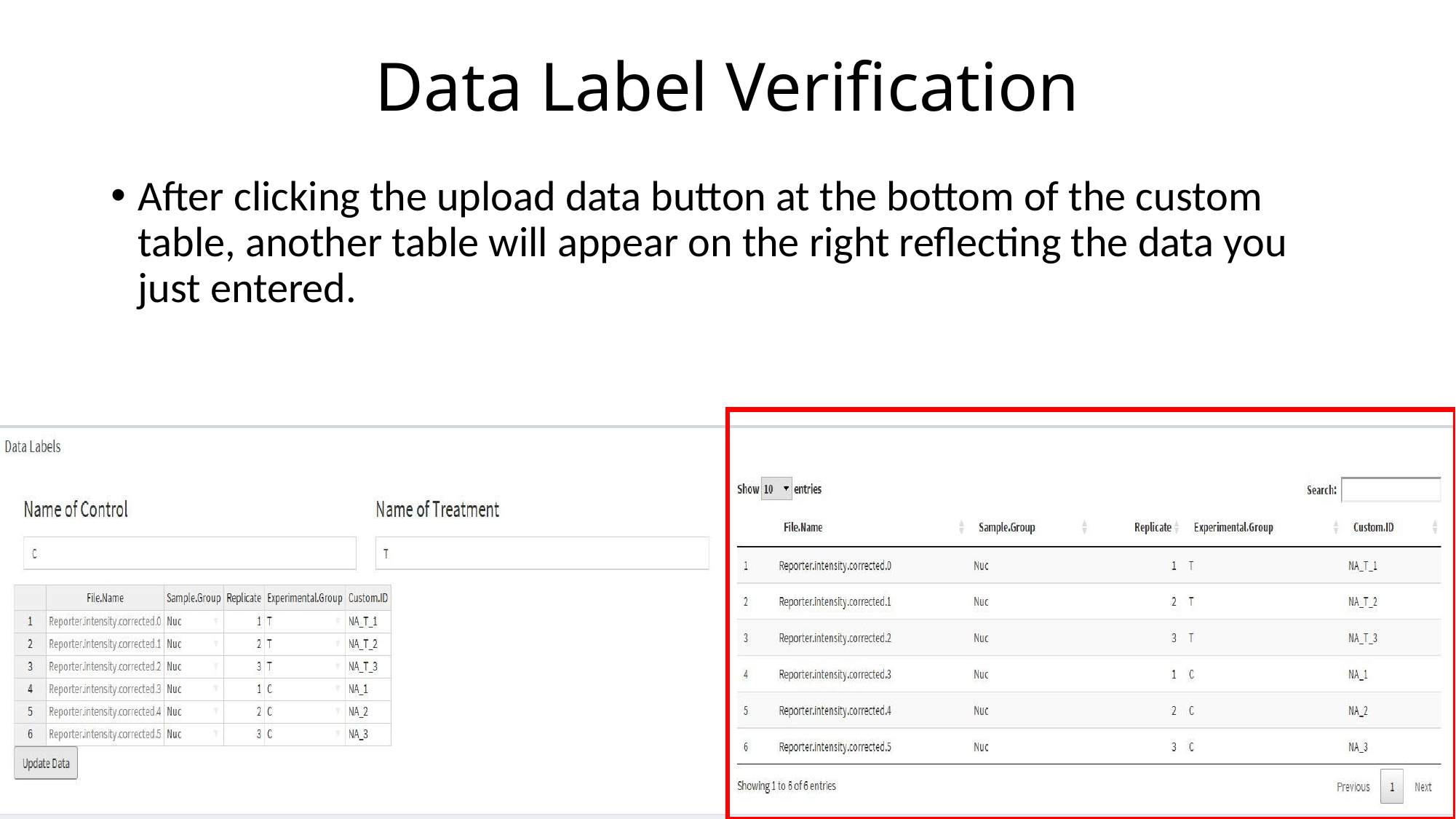

# Data Label Verification
After clicking the upload data button at the bottom of the custom table, another table will appear on the right reflecting the data you just entered.

## Slide 12
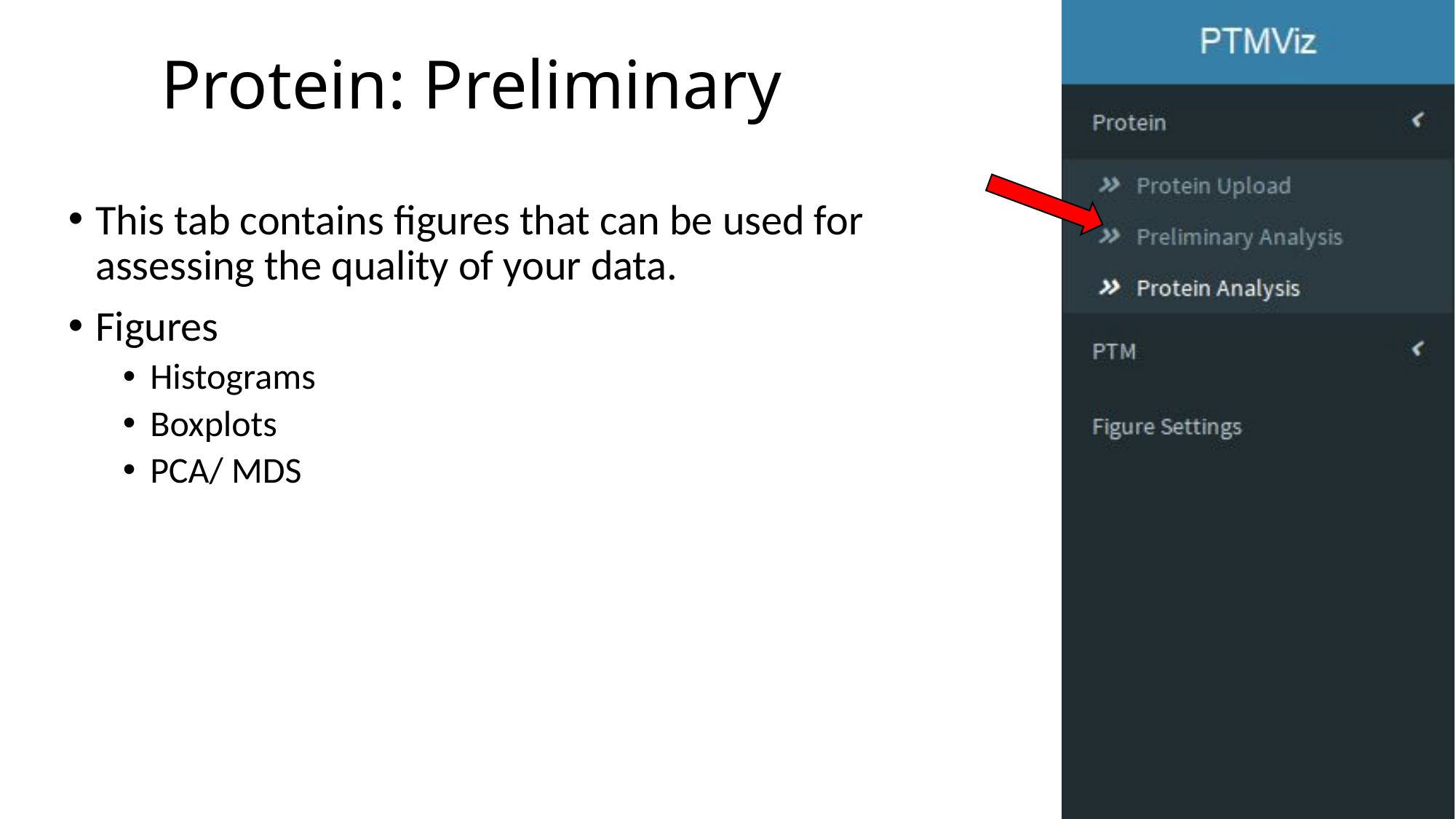

# Protein: Preliminary
This tab contains figures that can be used for assessing the quality of your data.
Figures
Histograms
Boxplots
PCA/ MDS

## Slide 13
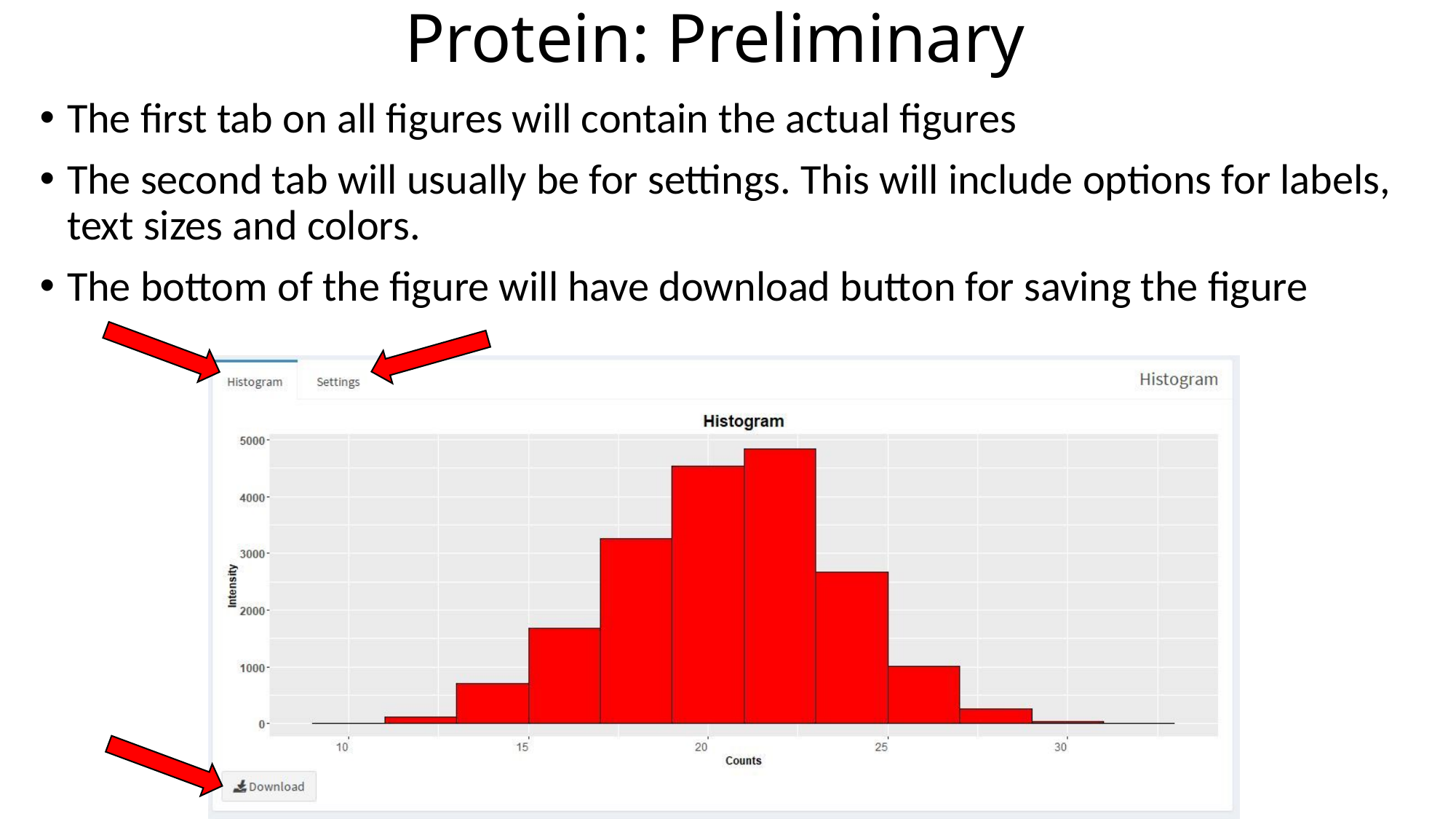

# Protein: Preliminary
The first tab on all figures will contain the actual figures
The second tab will usually be for settings. This will include options for labels, text sizes and colors.
The bottom of the figure will have download button for saving the figure

## Slide 14
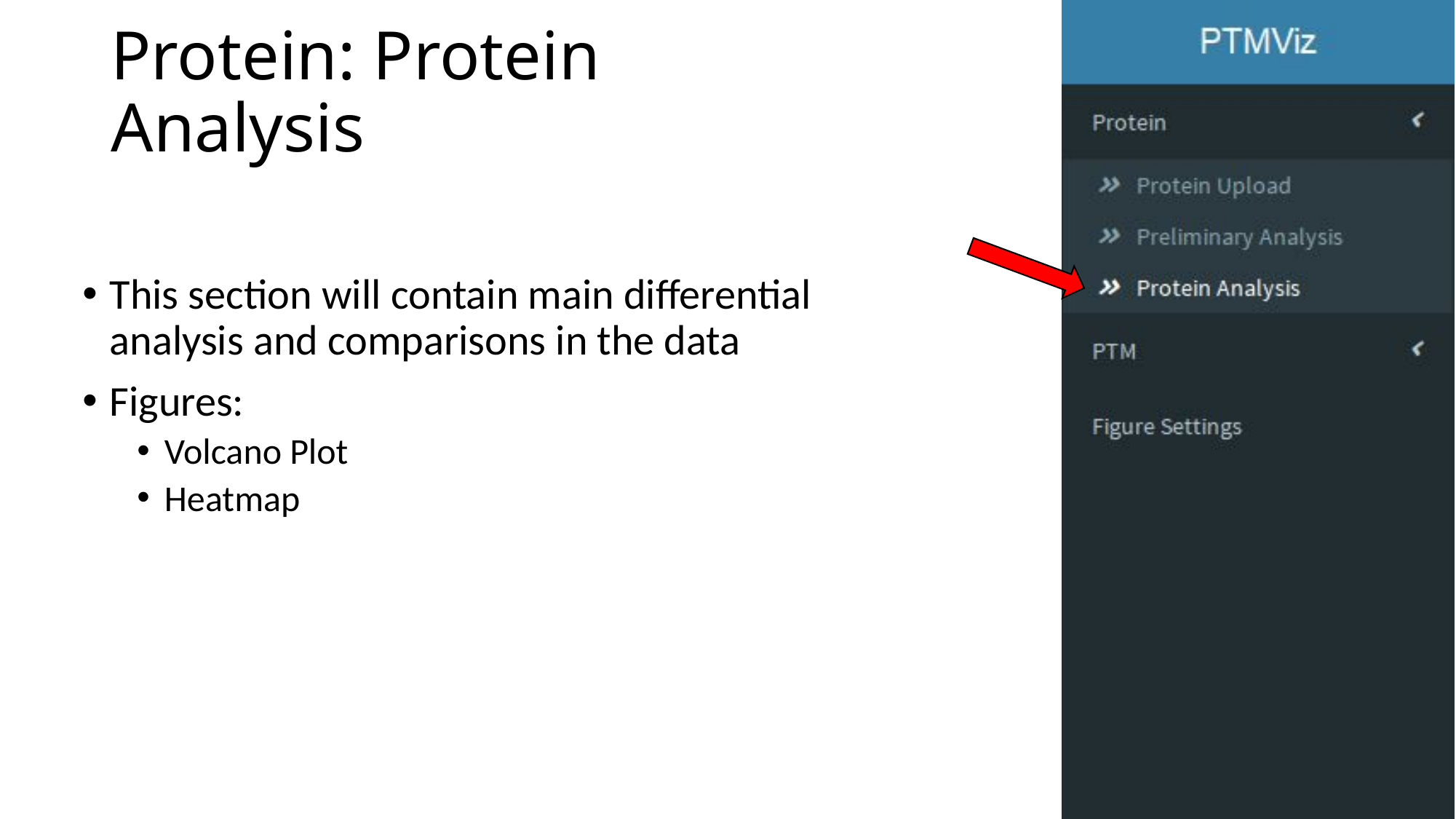

# Protein: Protein Analysis
This section will contain main differential analysis and comparisons in the data
Figures:
Volcano Plot
Heatmap

## Slide 15
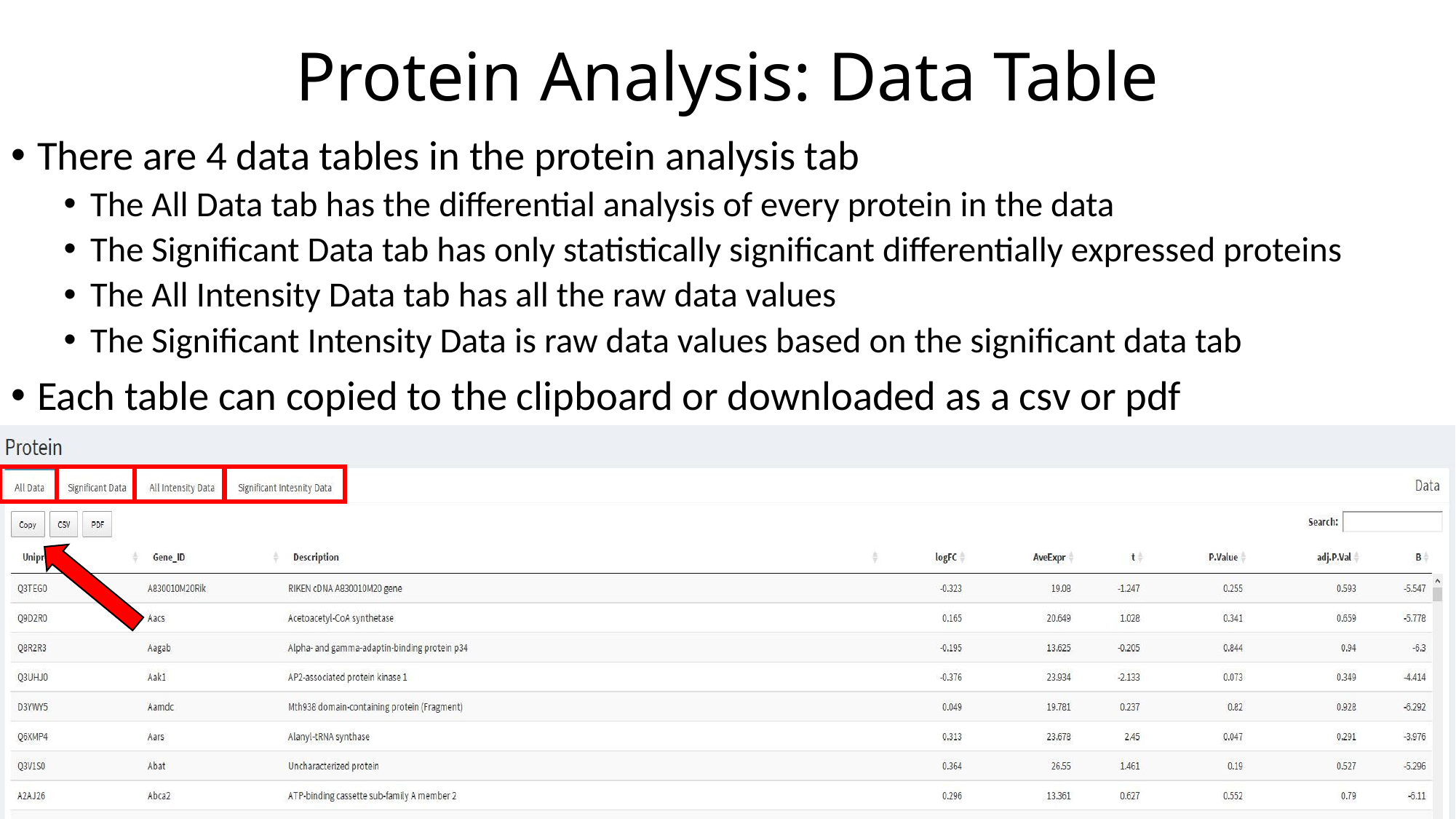

# Protein Analysis: Data Table
There are 4 data tables in the protein analysis tab
The All Data tab has the differential analysis of every protein in the data
The Significant Data tab has only statistically significant differentially expressed proteins
The All Intensity Data tab has all the raw data values
The Significant Intensity Data is raw data values based on the significant data tab
Each table can copied to the clipboard or downloaded as a csv or pdf

## Slide 16
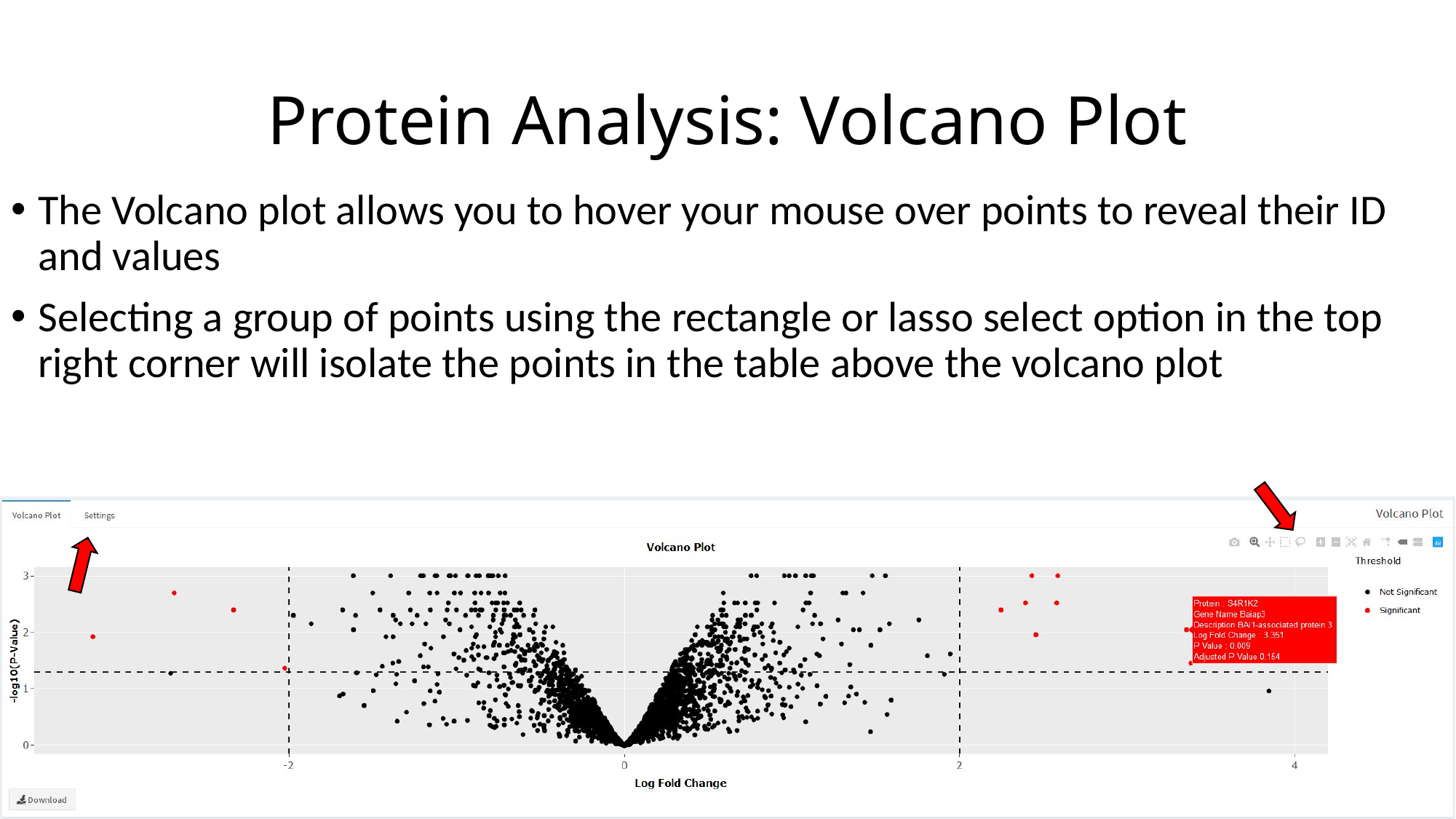

# Protein Analysis: Volcano Plot
The Volcano plot allows you to hover your mouse over points to reveal their ID and values
Selecting a group of points using the rectangle or lasso select option in the top right corner will isolate the points in the table above the volcano plot

## Slide 17
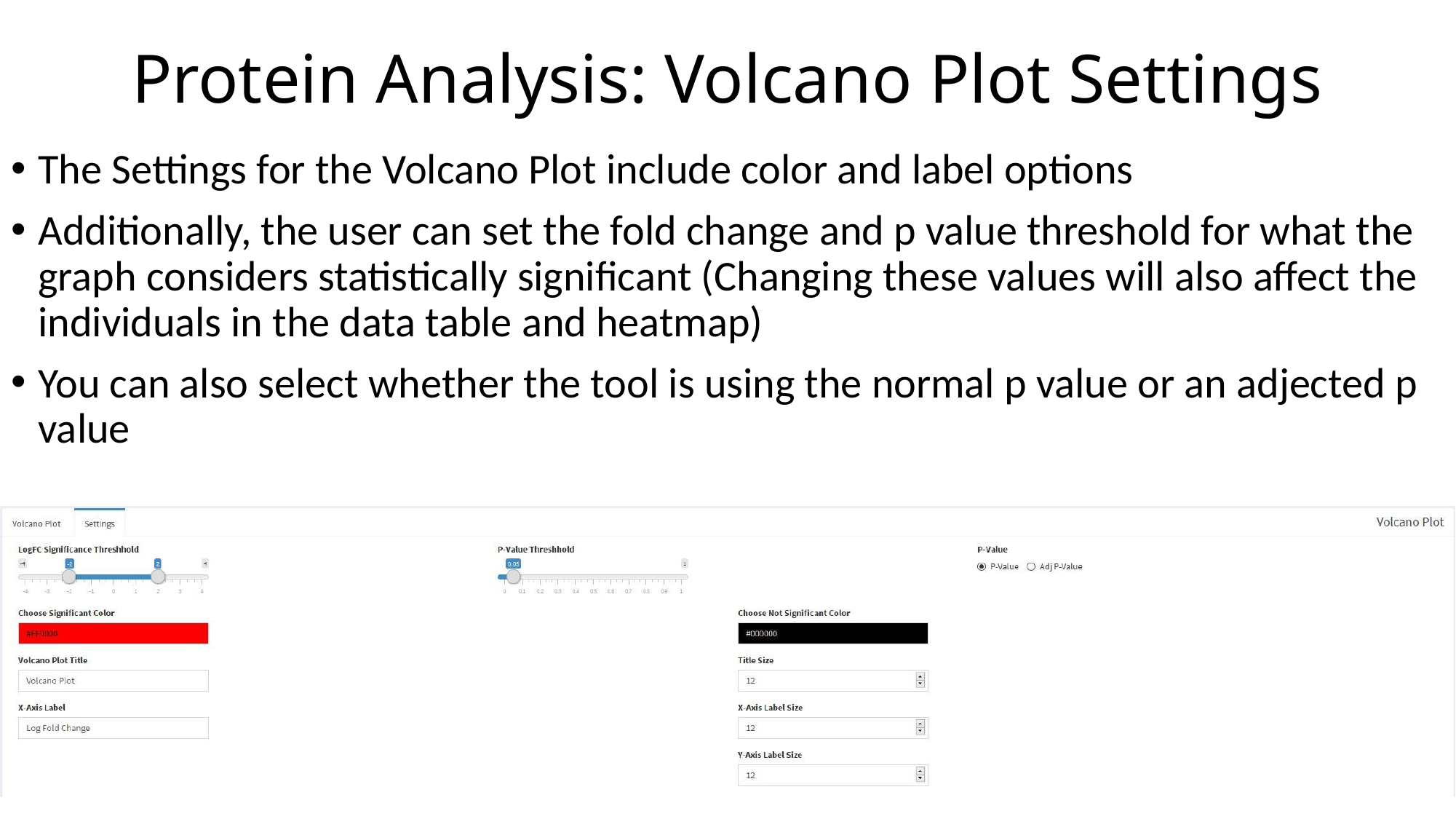

# Protein Analysis: Volcano Plot Settings
The Settings for the Volcano Plot include color and label options
Additionally, the user can set the fold change and p value threshold for what the graph considers statistically significant (Changing these values will also affect the individuals in the data table and heatmap)
You can also select whether the tool is using the normal p value or an adjected p value

## Slide 18
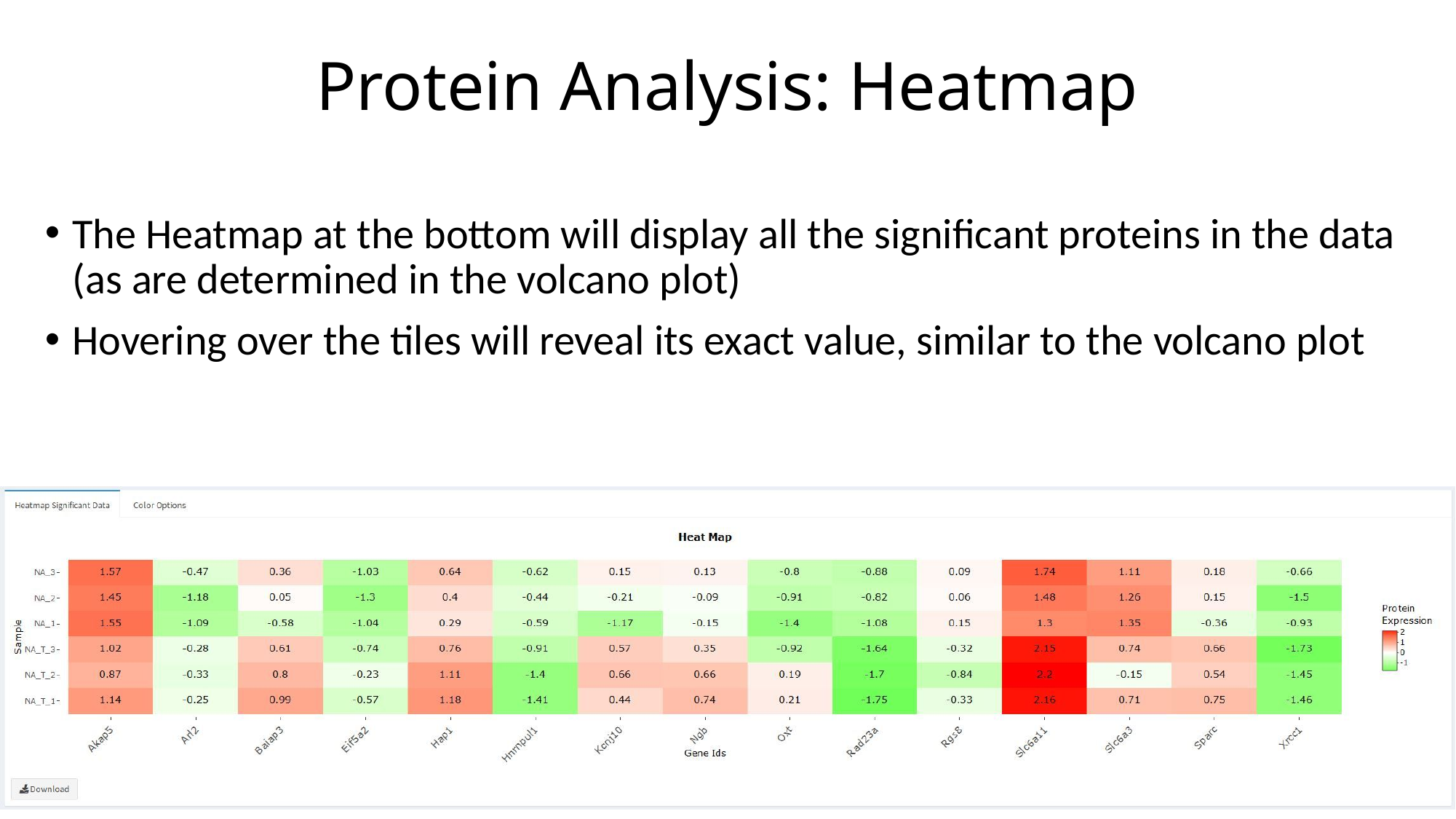

# Protein Analysis: Heatmap
The Heatmap at the bottom will display all the significant proteins in the data (as are determined in the volcano plot)
Hovering over the tiles will reveal its exact value, similar to the volcano plot

## Slide 19
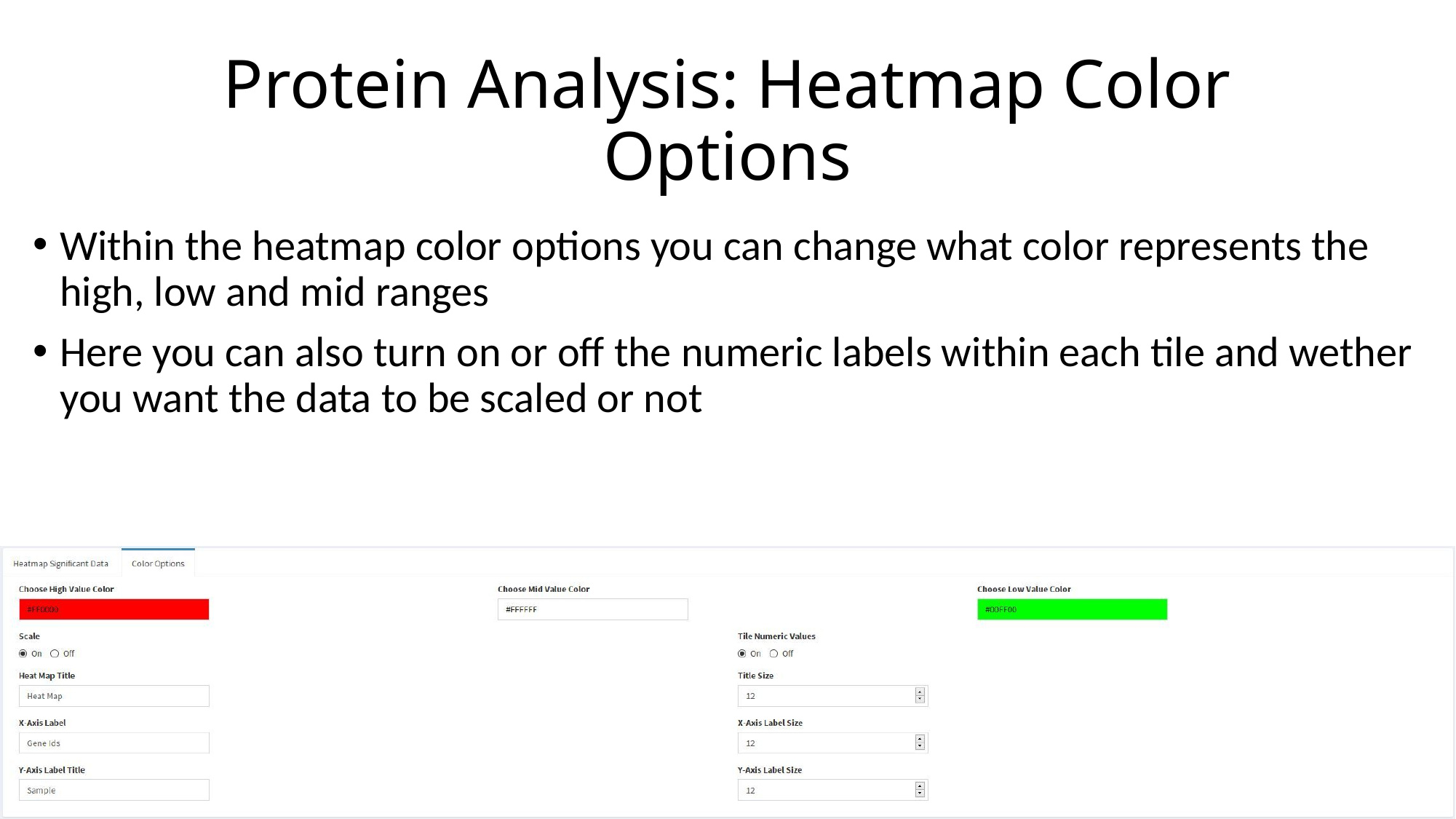

# Protein Analysis: Heatmap Color Options
Within the heatmap color options you can change what color represents the high, low and mid ranges
Here you can also turn on or off the numeric labels within each tile and wether you want the data to be scaled or not

## Slide 20
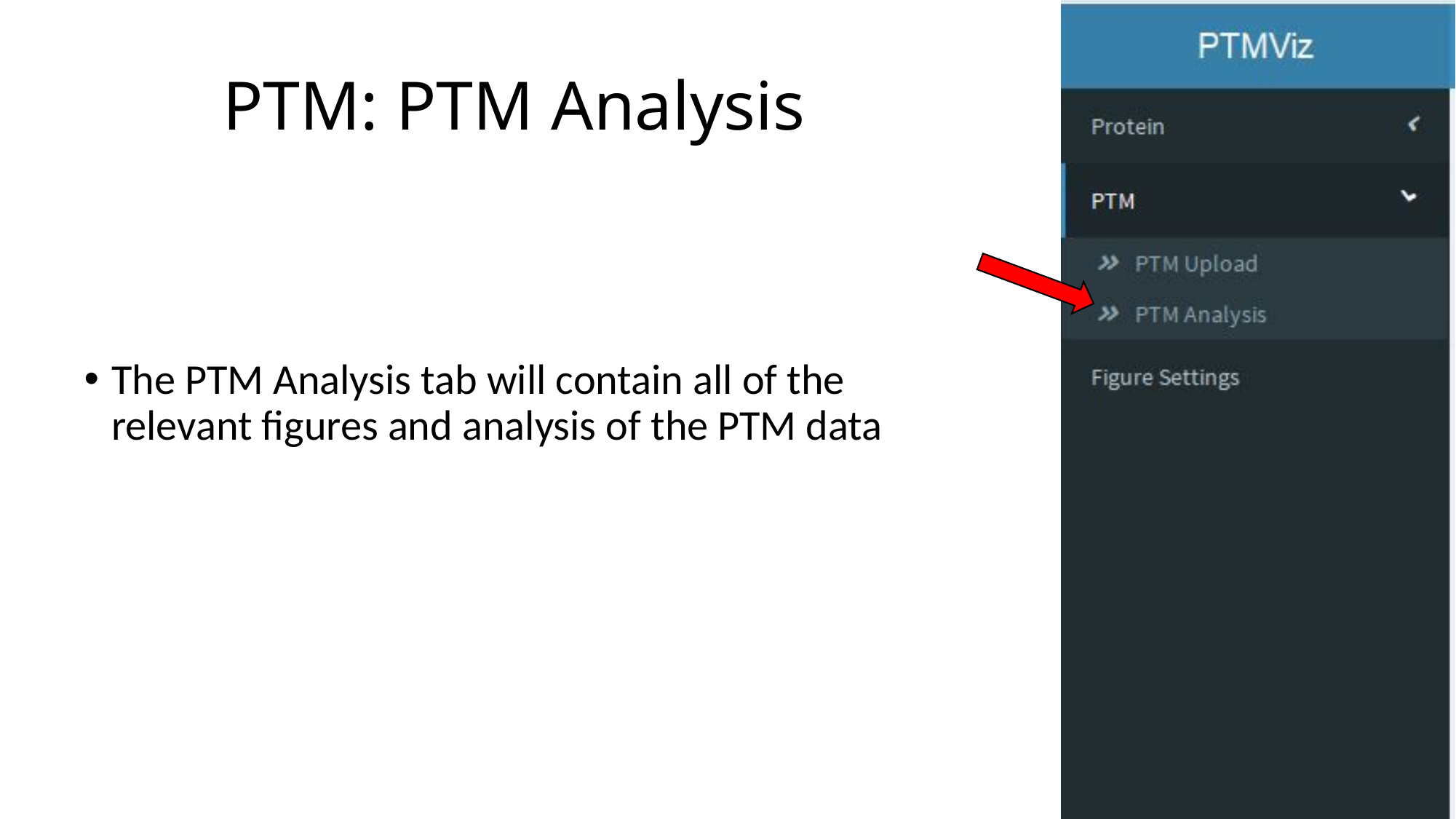

# PTM: PTM Analysis
The PTM Analysis tab will contain all of the relevant figures and analysis of the PTM data

## Slide 21
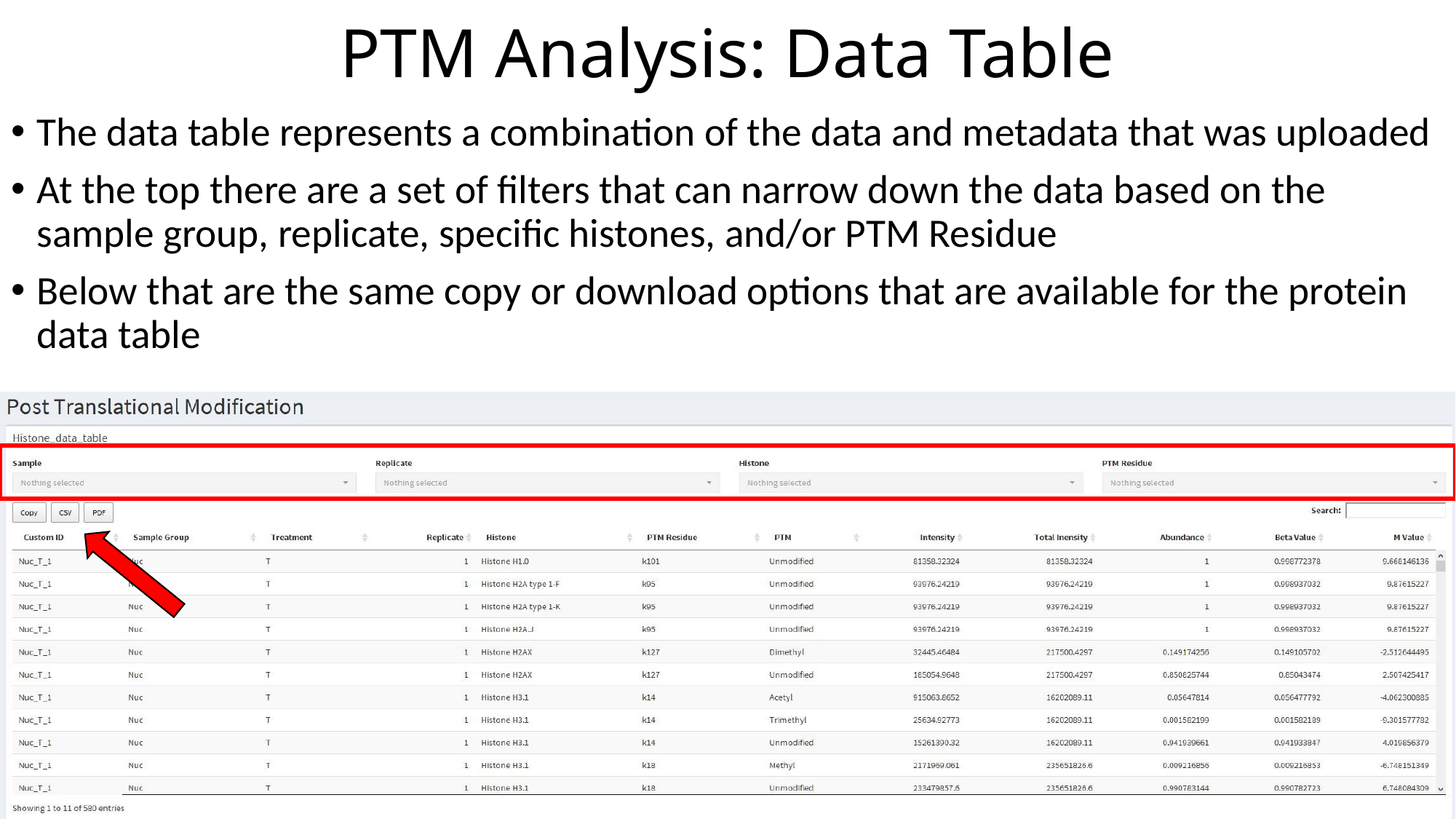

# PTM Analysis: Data Table
The data table represents a combination of the data and metadata that was uploaded
At the top there are a set of filters that can narrow down the data based on the sample group, replicate, specific histones, and/or PTM Residue
Below that are the same copy or download options that are available for the protein data table

## Slide 22
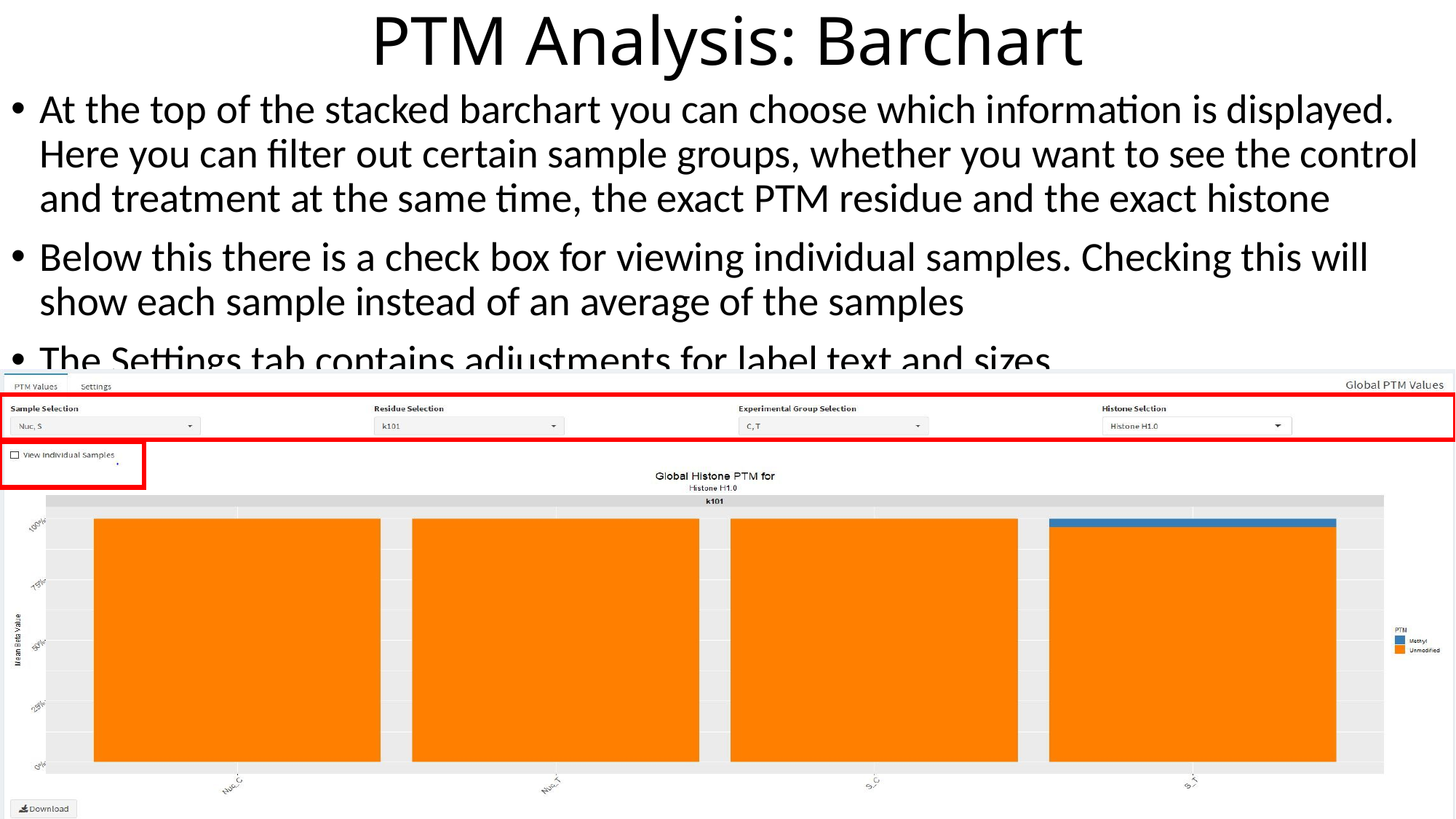

# PTM Analysis: Barchart
At the top of the stacked barchart you can choose which information is displayed. Here you can filter out certain sample groups, whether you want to see the control and treatment at the same time, the exact PTM residue and the exact histone
Below this there is a check box for viewing individual samples. Checking this will show each sample instead of an average of the samples
The Settings tab contains adjustments for label text and sizes

## Slide 23
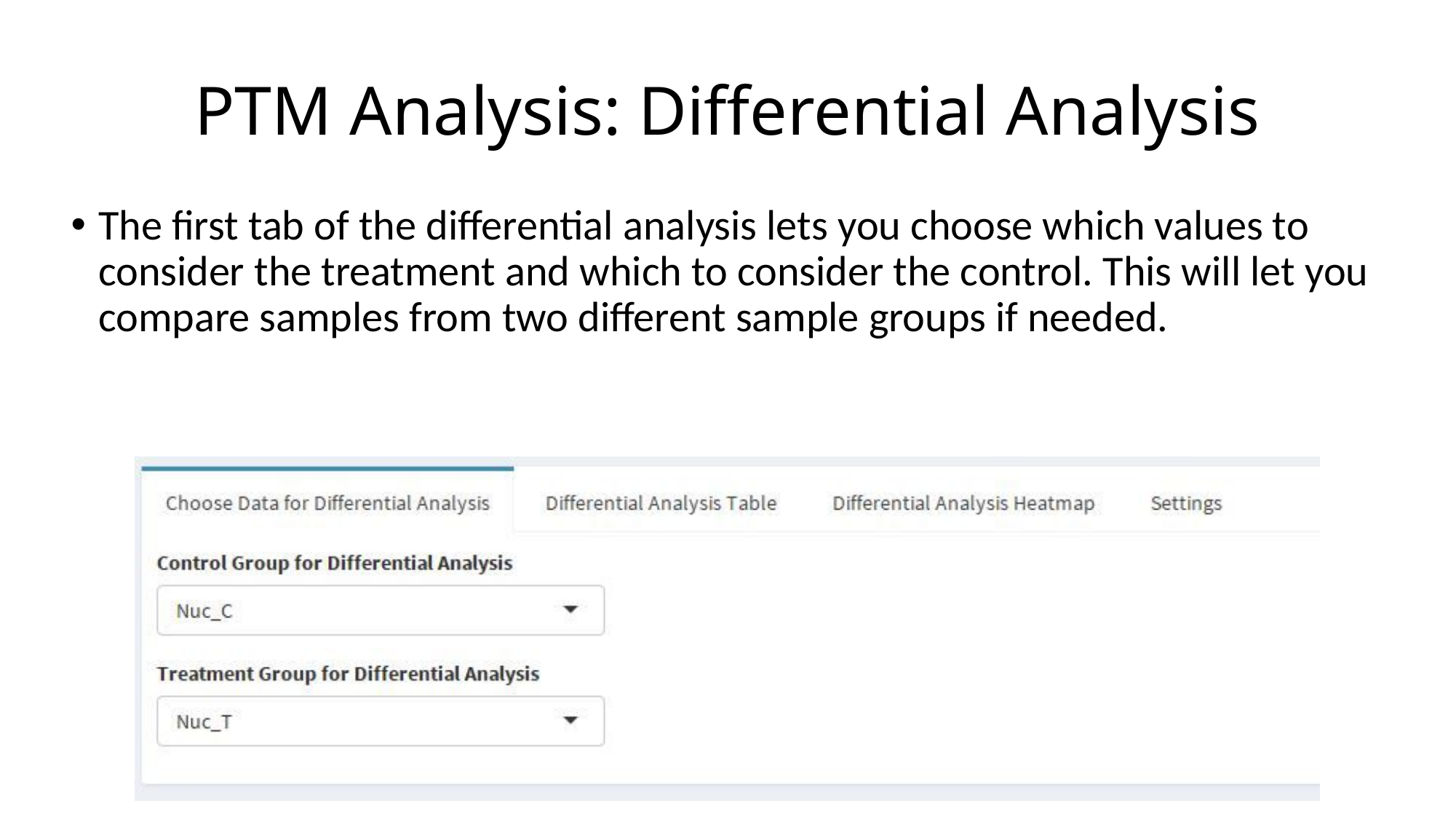

# PTM Analysis: Differential Analysis
The first tab of the differential analysis lets you choose which values to consider the treatment and which to consider the control. This will let you compare samples from two different sample groups if needed.

## Slide 24
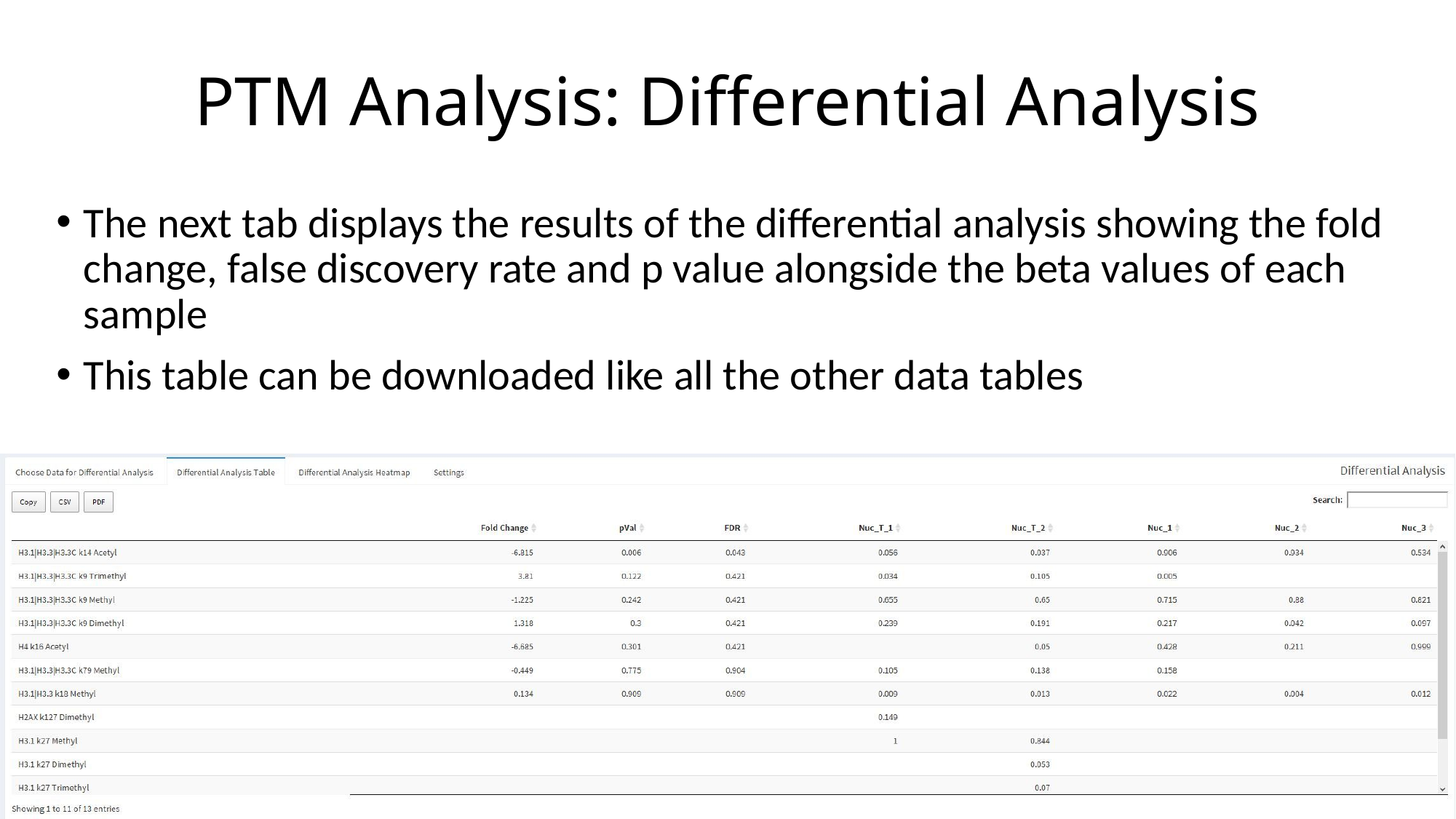

# PTM Analysis: Differential Analysis
The next tab displays the results of the differential analysis showing the fold change, false discovery rate and p value alongside the beta values of each sample
This table can be downloaded like all the other data tables

## Slide 25
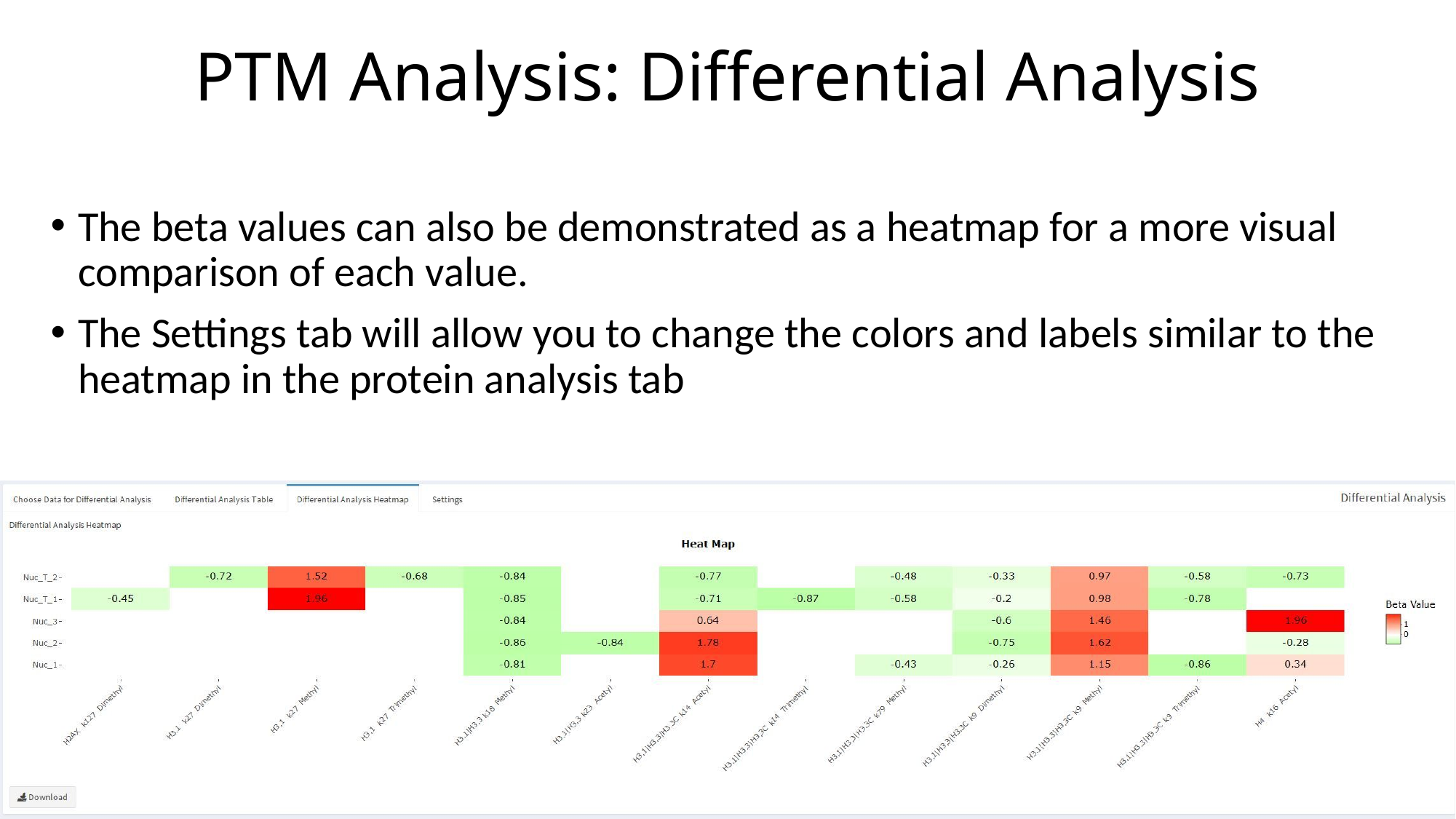

# PTM Analysis: Differential Analysis
The beta values can also be demonstrated as a heatmap for a more visual comparison of each value.
The Settings tab will allow you to change the colors and labels similar to the heatmap in the protein analysis tab

## Slide 26
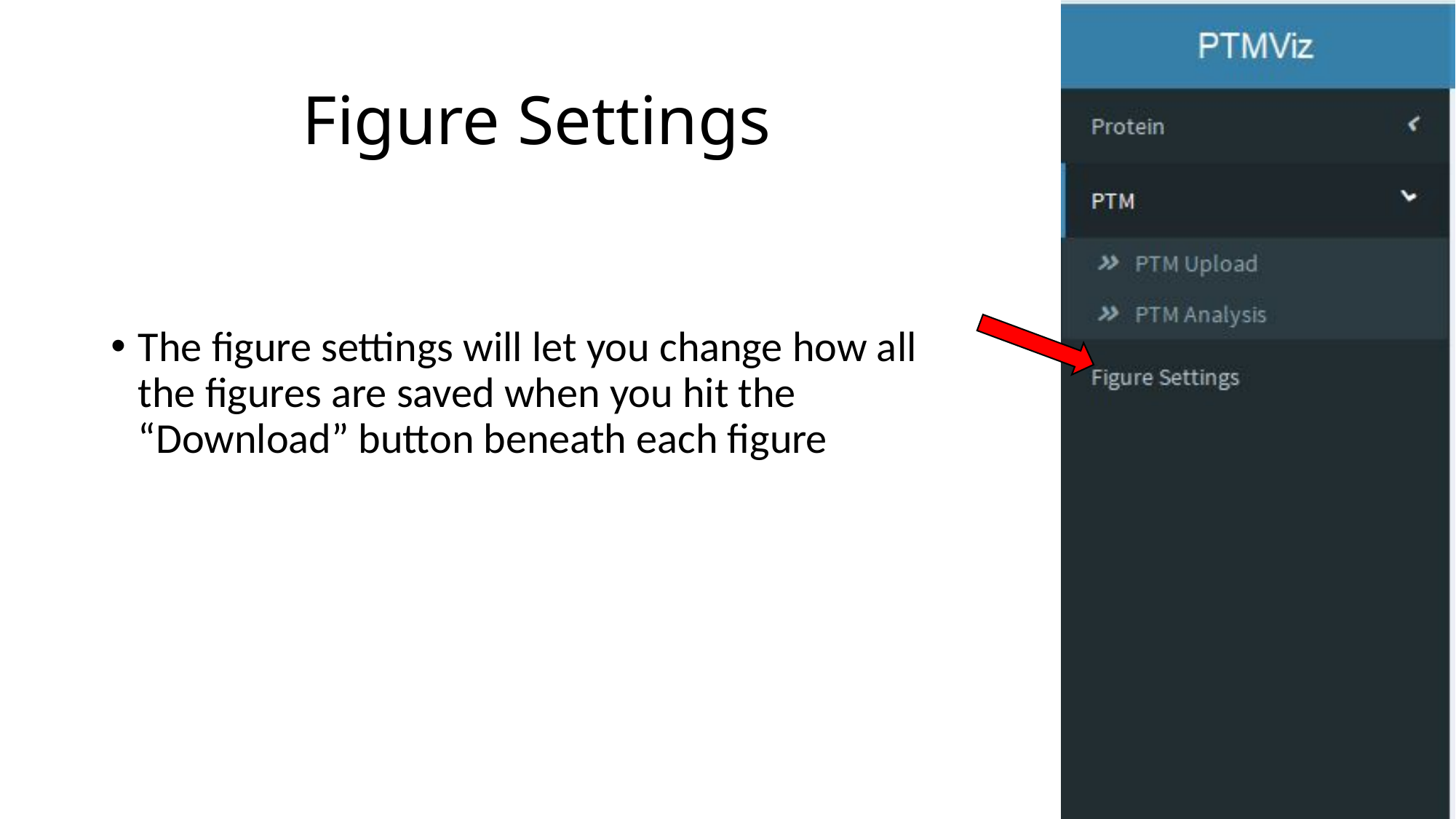

# Figure Settings
The figure settings will let you change how all the figures are saved when you hit the “Download” button beneath each figure

## Slide 27
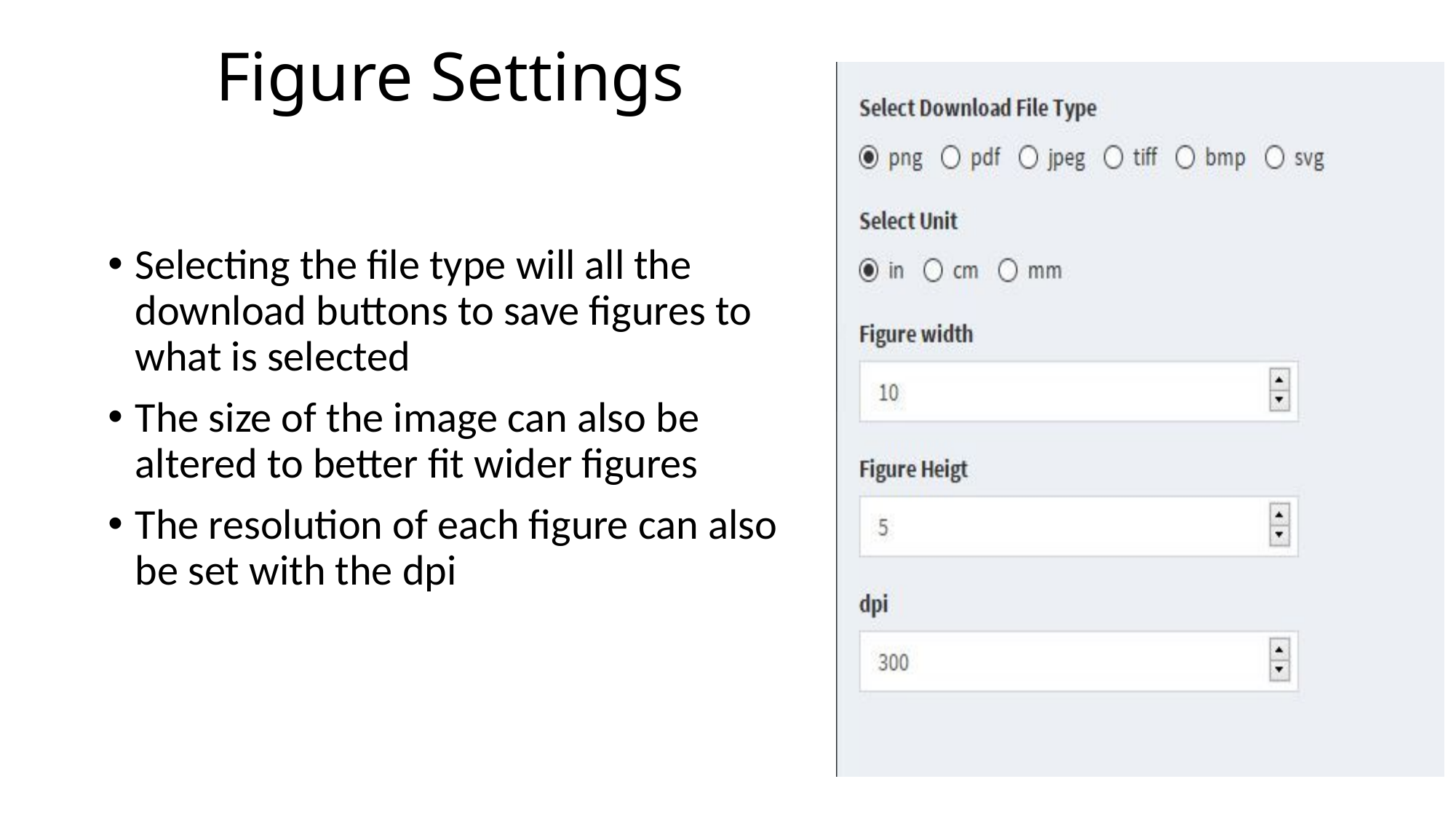

# Figure Settings
Selecting the file type will all the download buttons to save figures to what is selected
The size of the image can also be altered to better fit wider figures
The resolution of each figure can also be set with the dpi
