# Supplementary material for: PTMViz: a tool for analyzing and visualizing histone post translational modification data
Source: BMC Bioinformatics. 2021 May 26;22:275. doi: 10.1186/s12859-021-04166-9 (PMC8157737; doi:10.1186/s12859-021-04166-9)
Supplement: Supplementary file 2 — Additional file 2. Description of mass spectrometry methods. [file 12859_2021_4166_MOESM2_ESM.docx]

**Supplemental Methods**

*Animals and protein isolation*

All protocols and experiments were performed in accordance with and approved by the University of Arkansas for Medical Sciences Institutional Animal Care and Use Committee. Adult wild type male and female C57BL/6 mice were treated as described in Graw et al. Briefly, mice involved in the study were treated are as follows: saline-saline and methamphetamine (n=3/per group). All mice received intraperitoneal (ip) injections of either 1.5 mg/kg methamphetamine or saline every two hours for 4 consecutive injections. Mice were anesthetized with isoflurane and sacrificed 18 hour post the last injection [55]. The nucleus accumbens (NAc) and dorsal striatum regions of the mouse brain were extracted. The whole cell protein lysate as well as acid extracted histones were analyzed by mass spectrometry on an Orbitrap Fusion Lumos mass spectrometer (Thermo).

*Mass Spectrometry of Histones*

The washed brain regions were dounce homogenized in radioimmunoprecipitation assay buffer (RIPA) lysis buffer (10 mM Tris-Cl (pH 8.0), 1 mM Ethylenediaminetetraacetic acid (EDTA), 0.5 mM Ethylene glycol tetraacetic acid (EGTA), 1% Triton X-100, 0.1% sodium deoxycholate, 0.1% sodium dodecyl sulfate (SDS), and 140 mM NaCl) 5 times. The samples were incubated on ice for 30 min, spun at 4°C for 10 min at max speed, and the supernatant was transferred to a new tube for the whole lysate total protein analysis. The pellet was washed with RIPA and incubated with 0.4N H_2_SO_4_ at 4°C overnight. The samples were spun at 4°C for 10 min at max speed, the supernatant transferred to a new tube, added 66 µl of 100% TCA, incubated on ice for 30 min, spun at 4°C for 10 min at max speed, washed the pellet in ice cold acetone, air dried the pellet, and resuspended the histones in 50 µl of H_2_O.

Histones were resolved (20 µg of histones per lane) by SDS-PAGE using 4 –20% Novex Tris-glycine gradient gels (Life Technologies, Inc.) and stained with Thermo Fisher Scientific Pierce GelCode Blue stain reagent. The region of each gel lane containing the core histones was excised as one piece, diced into small pieces, destained, treated with d6-acetic anhydride to chemically block unmodified lysines and monomethylated lysines with an isotopically heavy acetyl, and digested in-gel with trypsin as reported previously [32]. Tryptic peptides were separated by reverse phase Jupiter Proteo resin (Phenomenex) on a 100 0.075-mm column using a nanoAcquity UPLC system (Waters). Peptides were eluted using a 40-min gradient from 97:3 to 35:65 buffer A/B ratio. (Buffer A consists of 0.1% formic acid, 0.5% acetonitrile; buffer B consists of 0.1% formic acid, 75% acetonitrile). Eluted peptides were ionized by electrospray (1.9 kV) followed by MS/MS analysis using collision induced dissociation on an Orbitrap Fusion mass spectrometer (Thermo Fisher Scientific). MS data were acquired using the Fourier Transform Mass Spectrometry analyzer in profile mode at a resolution of 60,000 over a range of 375 to 1500 m/z.

Proteins were identified by searching the UniProtKB database including only histone proteins (July 2016 database restricted to *Mus musculus,* 41 entries) using an in-house Mascot server (version 2.5.1; Matrix Science). Mascot search parameters were specified as follows: trypsin digestion with up to two missed cleavages; fixed carbamidomethyl modification of cysteine; variable modifications including methyl, dimethyl, trimethyl, acetyl, acetyl/2 H3, and methyl + acetyl/2 H3 modification of lysine; 2.0 ppm precursor ion tolerance; 0.50-Da fragment ion tolerance. A reverse sequence decoy search was also performed. Peptide and protein identifications were validated using Scaffold (version 4.8.7; Proteome Software). Peptide and protein identifications were accepted with a 1% FDR which was assigned by the Protein Prophet algorithm [36]. The spectrum report was then exported for further analysis by the PTMViz application.


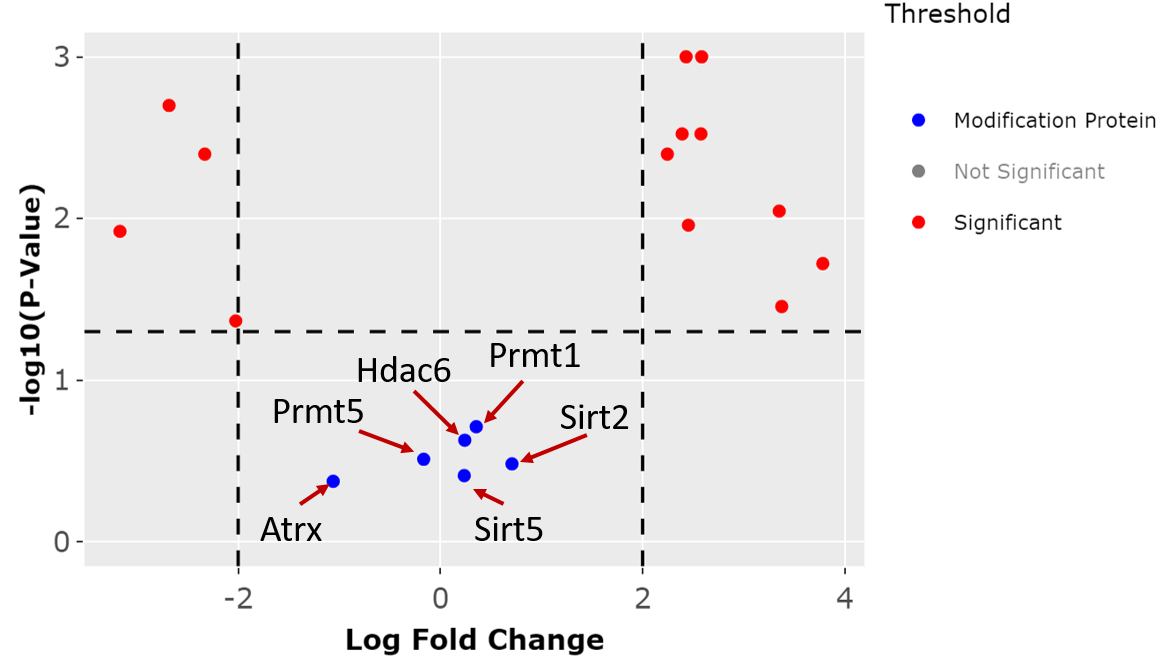


Figure S1. Protein Analysis Volcano plot. The Log2 fold change (treatment – control) is on the x-axis and the –log10 p-value is on the y-axis. The significant proteins (p-value < 0.05 and absolute fold change > 2) are highlighted in red. Proteins found in the WERAM database are highlighted in blue.


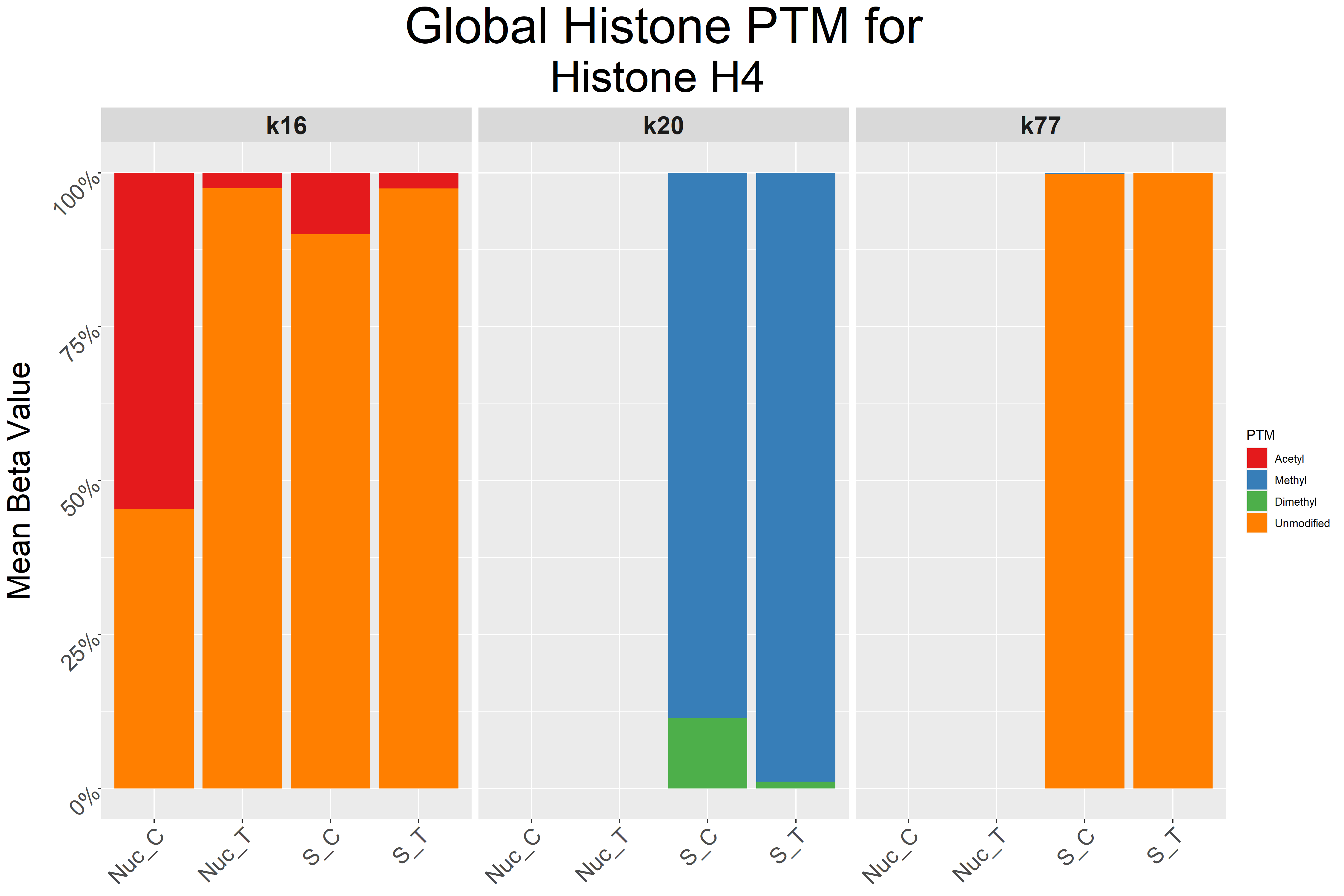


**Figure S2.** PTM analysis interactive figures. Stacked bar chart demonstrating the global PTMs identified for Histone H4. The mean of all sample replicates beta values for each group and PTM are displayed. The labels for each bar are determined by how samples are labeled in the metadata section of the tool, in this example Nucleus Accumbens and Striatum are abbreviated to Nuc and S, while the T and C denote whether it was a part of the treatment or control group.
